# Supplementary material for: Zika virus infection in pregnancy: a protocol for the joint analysis of the prospective cohort studies of the ZIKAlliance, ZikaPLAN and ZIKAction consortia
Source: BMJ Open. 2020 Dec 15;10(12):e035307. doi: 10.1136/bmjopen-2019-035307 (PMC7745317; doi:10.1136/bmjopen-2019-035307)
Supplement: Supplementary data [file bmjopen-2019-035307supp001.pdf]

SUPPLEMENTARY MATERIALS

Supplementary Table S1. Schedule of tests and examinations in pregnancy, at delivery, and during paediatric follow-up, in each consortium.

Consortium	Mother	Fetus	Newborn and neonate	Pediatric follow-up
ZIKAlliance	ZIKV serology IgG & IgM, NAAT blood and urine, at booking, every 4w, at birth, and at paediatric follow-up.<br><br>NAAT placenta and amniotic fluid at birth; NAAT breast milk and saliva at birth, if evidence of infection.<br><br>TORCH at enrolment / birth, HIV at birth according to local / national guidelines.	Laboratory, pathology examination of fetal loss and stillbirth.	Clinical examination of newborn.<br><br>Newborn testing: NAAT, IgM blood, cord blood; TORCH according to local / national guidelines.	Clinical examination, anthropometry, NAAT blood and urine; IgM, IgG blood at 4w, 4m, 12m, 24m.<br><br>Transcranial U/S at 4w. Hearing, eye, neurodevelopmental assessments.
ZIKAction	IgM, IgG serology booking, 20, 28, Delivery, and at paediatric follow-up<br><br>NAAT testing, and additional U/S investigations if evidence of infection.<br><br>PRNT for sub-set of samples.	Laboratory, pathology examination of fetal loss and stillbirth.	Clinical examination of newborn<br><br>Newborn testing: IgM, IgG (serum; CSF only if clinically indicated), urine, saliva. TORCH testing if clinically indicated<br><br>NAAT, U/S and ophthalmology investigations if evidence of infection	Paediatric testing:<br><br>IgG, IgM (serum, urine, saliva) and clinical examination at: 4w, 4m, 9m, 12m, 18m and 24m. NAAT if evidence on infection<br><br>Neurodevelopmental assessments

Ades AE, *et al. BMJ Open* 2020; 10:e035307. doi: 10.1136/bmjopen-2019-035307

ZikaPLAN	NAAT and serologic testing (PRNT, IgM, IgG3) when symptoms reported and at up to three follow-up visits.<br>TORCH, DENV, and CHIKV testing	Not routinely tested.	Clinical examination in early infancy.<br>NAAT testing in the neonatal period in some centres. TORCH testing if clinically indicated	Clinical examination of the child by specialists at 3, 6, 12, 18, 24, 36, and 48m<br>Neurodevelopmental assessments

CSF, Cerebro-spinal fluid; DENV, Dengue virus; CHIKV, Chikungunya virus; HIV, Human Immunodeficiency virus; Ig, Immunoglobulin; NAAT, Nucleic Acid Amplification Test; PRNT, Plaque Reduction Neutralisation Test; TORCH, Toxoplasmosis, Other, Rubella, Cytomegalovirus, Herpes; U/S, Ultrasound; ZIKV, Zika virus

Supplementary Table S2. Illustrative and approximate definitions of key explanatory and outcome variables. The final definitions will be determined by the Joint Diagnostics Group and Joint End-point Review Group.

Maternal Symptoms	For example: rash, fever, headache, joint pain
Maternal Infection in Pregnancy (MIP) status, established only on the basis of maternal testing	Confirmed: NAAT, Seroconversion<br>Suspected: Serological tests of recent infection, including: IgM, IgG3, avidity<br>No Evidence of MIP: Maternal testing protocol was followed, but none of the above were positive.<br>No MIP: As above but with IgG negative at or near time of delivery
Laboratory markers of Congenital Infection (CI)	Present: NAAT or IgM any time in first 7 days;<br>Absent: all other findings
Most likely trimester of onset of maternal infection	The highest available from the following hierarchy:<br>• Date of first NAAT positive test minus average duration of viremia<br>• Seroconversion: Midpoint between last serological negative and last positive<br>• Serological tests of recent infection: Date of first positive
Signs and symptoms compatible with Congenital Zika Syndrome (CZS)	A definition will be prepared by the Joint End-point Review Group, based on best evidence available. *The intention will be to produce a definition that is virtually 100% specific for congenital ZIKV infection in mothers exposed to a ZIKV outbreak, especially if other TORCH infections can be ruled out.*
Other Potentially Zika-related Outcomes (OPZRO)	A definition will be prepared by the Joint End-point Review Group, based on best evidence available.

Ig, Immunoglobulin; NAAT, Nucleic Acid Amplification Test.

Ades AE, *et al. BMJ Open* 2020; 10:e035307. doi: 10.1136/bmjopen-2019-035307

Supplementary Table S3. Prospectively ascertained Maternal Infection in Pregnancy (MIP) status and evidence on which it is based. NB: Data from the same woman can appear in more than one of the last four columns

Centre	Total women	Prospectively ascertained MIP status				Evidence for Confirmed or Suspected MIP			
		Confirmed MIP	Suspected MIP	No Evidence of MIP	No MIP	NAAT	Sero-conversion	IgM/ IgG3	PRNT
1									
2									
…									
etc									

Ig, Immunoglobulin; NAAT, Nucleic Acid Amplification Test; PRNT, Plaque Reduction Neutralisation Test.

Supplementary Table S4. Summary of evidence on laboratory and clinical markers of congenital infection (CI) and evidence on which it is based. NB: Data from the same fetus/newborn can appear in more than one column

		Markers of congenital infection						Other evidence of congenital infection			
Centre	Total number fetus/newborn	Laboratory or clinical markers of congenital infection	Laboratory markers of congenital infection only	Signs and symptoms compatible with CZS	NAAT	IgM	No markers of congenital infection	Microcephaly	Neurological abnormalities	Ocular abnormalities	Other, to be defined
1											
2											
…											
etc											

CZS, Symptoms compatible with Congenital Zika Syndrome; IgM/G, Immunoglobulin M/G; NAAT, Nucleic Acid Amplification Test; OPZRO, Other potentially Zika-related symptoms; PRNT, Plaque Reduction Neutralisation Test.

Ades AE, *et al. BMJ Open* 2020; 10:e035307. doi: 10.1136/bmjopen-2019-035307

Supplementary Table S5. Distributions of signs and symptoms by laboratory markers of congenital infection and prospectively ascertained maternal infection in pregnancy (MIP) status**.** The breakdown shown is an example. Other examples might be "Termination of pregnancy, Pregnancy loss, Stillbirth, Livebirth"; or "Sensorineural hearing loss (SNHL), No SNHL "

**CENTRE**			
**Sign or symptom in fetus, newborn, or infant**	**MIP with laboratory markers of congenital infection**	**MIP with no laboratory markers of congenital infection**	**No MIP**
Signs and symptoms compatible with Congenital Zika Syndrome (CZS)			
Other potentially Zika-related outcomes (OPZRO)			
Asymptomatic			
**TOTAL = 100%**			
