# Supplementary material for: Zika virus infection in pregnancy: a protocol for the joint analysis of the prospective cohort studies of the ZIKAlliance, ZikaPLAN and ZIKAction consortia
Source: BMJ Open. 2020 Dec 15;10(12):e035307. doi: 10.1136/bmjopen-2019-035307 (PMC7745317; doi:10.1136/bmjopen-2019-035307)
Supplement: Author's manuscript [file bmjopen-2019-035307.draft_revisions.pdf]

BMJ Open

BMJ Open is committed to open peer review. As part of this commitment we make the peer review history of every article we publish publicly available.

When an article is published we post the peer reviewers' comments and the authors' responses online. We also post the versions of the paper that were used during peer review. These are the versions that the peer review comments apply to.

The versions of the paper that follow are the versions that were submitted during the peer review process. They are not the versions of record or the final published versions. They should not be cited or distributed as the published version of this manuscript.

BMJ Open is an open access journal and the full, final, typeset and author-corrected version of record of the manuscript is available on our site with no access controls, subscription charges or pay-per-view fees (http://bmjopen.bmj.com).

If you have any questions on BMJ Open's open peer review process please email info.bmjopen@bmj.com

BMJ Open

Zika virus infection in pregnancy: A protocol for the joint analysis of the prospective cohort studies of the ZIKAlliance, ZikaPLAN and ZIKAction consortia

Journal:	*BMJ Open*
Manuscript ID	bmjopen-2019-035307
Article Type:	Protocol
Date Submitted by the Author:	27-Oct-2019
Complete List of Authors:	Ades, A E; University of Bristol<br>Brickley, Elizabeth B.; London School of Hygiene and Tropical Medicine, Department of Infectious Disease Epidemiology<br>Alexander, Neal; London School of Hygiene and Tropical Medicine Faculty of Epidemiology and Population Health<br>Brown, David; 4. Fundacão Oswaldo Cruz, Flavivirus Reference Laboratory<br>Jaenisch, Thomas ; UniversitatsKlinikum Heidelberg, Department of Infectious Diseases, Section Clinical Tropical Medicine;<br>Miranda-Filho, Demócrito de Barros; University of Pernambuco, Faculty of Medical Sciences<br>Pohl, Moritz; 7. Institute of Medical Biometry and Informatics, University of Heidelberg<br>Rosenberger, Kerstin Daniela; UniversitatsKlinikum Heidelberg, Department of Infectious Diseases, Section Clinical Tropical Medicine<br>Soriano-Arandes, Antoni; University Hospital Vall d'Hebron, Paediatric Infectious Diseases and Immunodeficiencies Unit, Department of Pediatrics<br>Thorne, Claire; University College London, Great Ormond Street Institute of Child Health<br>Arraes de Alencar Ximenes, Ricardo; Federal University of Pernambuco, Department of Tropical Medicine<br>Velho Barreto de Araújo, Thalia; Universidade Federal de Pernambuco, Department of Social Medicine<br>Avelino-Silva, Vivian ; Faculdade de Medicina da Universidade de Sao Paulo, Department of Infectious and Parasitic Diseases<br>Bethencourt, Sarah ; Universidad de Carabobo, Facultad de Ciencias de la Salud<br>Borja-Aburto, Victor; Mexican Institute of Social Security<br>Brasil, Patrícia ; Fundacao Oswaldo Cruz, Instituto de pesquisa Clínica Evandro Chagas<br>Christie, C. D. C.; University of the West Indies at Mona, Department of Child and Adolescent Health<br>DE SOUZA, Wayner; Instituto Aggeu Magalhães, Fundação Oswaldo Cruz<br>GOTUZZO H., Jose Eduardo ; Universidad Peruana Cayetano Heredia Instituto de Medicina Tropical Alexander von Humboldt<br>Hoen, Bruno; INSERM Centre d'Investigation Clinique 1424, Centre Hospitalier Universitaire de Pointe-à-Pitre; Centre Hospitalier Universitaire de Pointe-à-Pitre

	Koopmans, M; Erasmus Universiteit Rotterdam, Department of Viroscience<br>Turchi Martelli, Celina Maria; Oswaldo Cruz Foundation , Department of Collective Health, Institute Aggeu Magalhães (CPqAM)<br>Teixeira, Mauro; Universidade Federal de Minas Gerais<br>Marques, Ernesto; Instituto Aggeu Magalhães, Fundação Oswaldo Cruz; University of Pittsburgh, Department of Infectious Diseases and Microbiology<br>Miranda Montoya, Maria Consuelo ; Universidad Industrial de Santander, Facultad de Salud ; Universidad Industrial de Santander, Centro de >Investigaciones Epidemiológicas<br>Ramos Montarroyos, Ulisses; 26. Instituto de Ciências Biológicas, Universidade de Pernambuco<br>Moreira, Maria ; Figueira National Institute for Women's, Children's and Adolescents Health, Oswaldo Cruz Foundation<br>Morris, J; University of Florida, Emerging Pathogens Institute<br>Rockx, Barry; Erasmus Universiteit Rotterdam, Department of Viroscience<br>SABA VILLARROEL, Paola Mariela ; Centro Nacional de Enfermedades Tropicales, Santa Cruz de la Sierra<br>SORIA SEGARRA, Carmen ; Universidad Católica de Santiago de Guayaquil; SOSECALI C., Ltda<br>Tami, Adriana; University Medical Center Groningen, Department of Medical Microbiology and Infection Prevention<br>Turchi, Marília Dalva; Federal University of Goias, Institute of Tropical Pathology and Public Health<br>Giaquinto, Carlo; University of Padova Faculty of Medicine and Surgery, Department of Woman's and Child's Health<br>de-Lamballerie, Xavier; Aix-Marseille Université Institut Universitaire de Technologie d'Aix-en-Provence<br>Wilder-Smith, Annelies; Umeå University

Keywords:	EPIDEMIOLOGY, Public health < INFECTIOUS DISEASES, Paediatric infectious disease & immunisation < PAEDIATRICS

SCHOLARONE™
Manuscripts

I, the Submitting Author has the right to grant and does grant on behalf of all authors of the Work (as defined in the below author licence), an exclusive licence and/or a non-exclusive licence for contributions from authors who are: i) UK Crown employees; ii) where BMJ has agreed a CC-BY licence shall apply, and/or iii) in accordance with the terms applicable for US Federal Government officers or employees acting as part of their official duties; on a worldwide, perpetual, irrevocable, royalty-free basis to BMJ Publishing Group Ltd ("BMJ") its licensees and where the relevant Journal is co-owned by BMJ to the co-owners of the Journal, to publish the Work in this journal and any other BMJ products and to exploit all rights, as set out in our licence.

The Submitting Author accepts and understands that any supply made under these terms is made by BMJ to the Submitting Author unless you are acting as an employee on behalf of your employer or a postgraduate student of an affiliated institution which is paying any applicable article publishing charge ("APC") for Open Access articles. Where the Submitting Author wishes to make the Work available on an Open Access basis (and intends to pay the relevant APC), the terms of reuse of such Open Access shall be governed by a Creative Commons licence – details of these licences and which Creative Commons licence will apply to this Work are set out in our licence referred to above.

Other than as permitted in any relevant BMJ Author's Self Archiving Policies, I confirm this Work has not been accepted for publication elsewhere, is not being considered for publication elsewhere and does not duplicate material already published. I confirm all authors consent to publication of this Work and authorise the granting of this licence.

For peer review only

Zika virus infection in pregnancy: A protocol for the joint analysis of the prospective cohort studies of the ZIKAlliance, ZikaPLAN and ZIKAction consortia

A. E. ADES[1], Elizabeth B. BRICKLEY[2], Neal ALEXANDER[3], David BROWN[4], Thomas JAENISCH[5], Demócrito de Barros MIRANDA-FILHO[6], Moritz POHL[7], Kerstin D. ROSENBERGER[5], Antoni SORIANO-ARANDES[8], Claire THORNE[9], Ricardo Arraes de Alencar XIMENES[6,10], Thália Velho Barreto de ARAÚJO[11], Vivian I. AVELINO-SILVA[12], Sarah Esperanza BETHENCOURT CASTILLO[13], Victor Hugo BORJA ABURTO[14], Patricia BRASIL[15], Celia D. C. CHRISTIE[16], Wayner Vieira DE SOUZA[17], Jose Eduardo GOTUZZO H.[18], Bruno HOEN[19,20,21], Marion KOOPMANS[22], Celina Maria Turchi MARTELLI[17], Mauro MARTINS TEIXEIRA[23], Ernesto T. A. MARQUES[17,24], Maria Consuelo MIRANDA[25], Ulisses Ramos MONTARROYOS[26], Maria Elisabeth MOREIRA[27], J Glenn MORRIS[28], Barry ROCKX[22], Paola Mariela SABA VILLARROEL[29], Carmen SORIA SEGARRA[30,31], Adriana TAMI[13,32], Marília Dalva TURCHI[33], Carlo GIAQUINTO[34] Xavier de LAMBALLERIE[35], Annelies WILDER-SMITH[36], on behalf of the EC Zika Consortia Vertical Transmission Study Group

1. Department of Population Health Sciences, Bristol Medical School, University of Bristol, Bristol, United Kingdom
2. Department of Infectious Disease Epidemiology, Faculty of Epidemiology and Population Health, London School of Hygiene & Tropical Medicine, London, United Kingdom
3. MRC Tropical Epidemiology Group, Faculty of Epidemiology and Population Health, London School of Hygiene & Tropical Medicine, London, United Kingdom
4. Fundacão Oswaldo Cruz, Flavivirus Reference Laboratory, Rio de Janeiro, Rio de Janeiro, Brazil
5. Section Clinical Tropical Medicine, Department of Infectious Diseases, Heidelberg University Hospital, Heidelberg, Germany
6. Departamento de Medicina Interna, Universidade de Pernambuco, Recife, Pernambuco, Brazil
7. Institute of Medical Biometry and Informatics, University of Heidelberg, Heidelberg, Germany
8. Pediatric Infectious Diseases and Immunodeficiencies Unit, Hospital Universitari Vall d'Hebron, Vall d'Hebron Research Institute, Barcelona, Spain
9. Great Ormond Street Institute of Child Health, University College London, United Kingdom
10. Departamento de Medicina Tropical, Universidade Federal de Pernambuco, Recife, Pernambuco, Brazil
11. Departamento de Medicina Social, Universidade Federal de Pernambuco, Recife, Pernambuco, Brazil
12. Department of Infectious and Parasitic Diseases and Instituto de Medicina Tropical, Faculdade de Medicina da Universidade de Sao Paulo, Sao Paulo, Brazil
13. Facultad de Ciencias de la Salud, Universidad de Carabobo, Valencia, Venezuela
14. Mexican Institute of Social Security, Mexico City, Mexico
15. Evandro Chagas National Institute of Infectious Diseases, Fundação Oswaldo Cruz, Rio de Janeiro, Rio de Janeiro, Brazil
16. Department of Child and Adolescent Health, University of the West Indies, Mona Campus, Kingston, Jamaica
17. Instituto Aggeu Magalhães, Fundação Oswaldo Cruz, Recife, Pernambuco, Brazil
18. Instituto de Medicina Tropical Alexander von Humboldt, Universidad Peruana Cayetano Heredia, Lima, Peru
19. INSERM Centre d'Investigation Clinique 1424, Centre Hospitalier Universitaire de Pointe-à-Pitre, Guadeloupe, France

For peer review only - http://bmjopen.bmj.com/site/about/guidelines.xhtml

20. Service de Maladies Infectieuses et Tropicales, Dermatologie, Médecine Interne, Centre Hospitalier Universitaire de Pointe-à-Pitre, Guadeloupe, France
21. Université des Antilles et de la Guyane, Faculté de Médecine Hyacinthe Bastaraud, EA 4537, Pointe-à-Pitre, Guadeloupe, France.
22. Department of Viroscience, Erasmus Medical Center, Rotterdam, The Netherlands
23. Universidade Federal de Minas Gerais, Belo Horizonte, Minas Gerais, Brazil
24. Department of Infectious Diseases and Microbiology, University of Pittsburgh, Pittsburgh, Pennsylvania, United States of America
25. Universidad Industrial de Santander, Bucaramanga, Colombia
26. Instituto de Ciências Biológicas, Universidade de Pernambuco, Recife, Pernambuco, Brazil
27. Instituto Fernandes Figueira – Fundação Oswaldo Cruz, Rio de Janeiro, Rio de Janeiro, Brazil
28. Emerging Pathogens Institute, University of Florida, Gainesville, Florida, United States of America
29. Centro Nacional de Enfermedades Tropicales, Santa Cruz de la Sierra, Bolivia
30. Universidad Católica de Santiago de Guayaquil, Ecuador
31. SOSECALI C., Ltda, Guayaquil, Ecuador
32. University of Groningen, University Medical Center Groningen, Department of Medical Microbiology and Infection Prevention, The Netherlands
33. Instituto de Patologia Tropical e Saúde Publica, Universidade Federal de Goiás, Goiânia, Goiás, Brazil.
34. Dipartimento di Salute della Donna e del Bambino, Università degli Studi di Padova, Italy
35. Université *d'*Aix-Marseille, Marseille, France
36. Department of Epidemiology and Global Health, Umeå Universite, Umeå, Sweden

Corresponding author:

Claire Thorne, Population Policy and Practice Programme, University College London Great Ormond Street Institute of Child Health, 30 Guilford Street, London WC1N 1EH, United Kingdom

Tel: +44 (0) 2079052105

claire.thorne@ucl.ac.uk

Word count 3901

For peer review only

ABSTRACT

Introduction

Zika virus (ZIKV) infection in pregnancy has been associated with microcephaly and severe neurological damage to the fetus. Our aim is to document the risks of adverse pregnancy and birth outcomes and the prevalence of laboratory markers of congenital infection in deliveries to women experiencing ZIKV infection during pregnancy, using data from European Commission-funded prospective cohort studies in 20 centres in 11 countries across Latin America and the Caribbean.

Methods and analysis

We will carry out a centre-by-centre analysis of the risks of adverse pregnancy and birth outcomes, comparing women with confirmed and suspected ZIKV infection in pregnancy to those with no evidence of infection in pregnancy. We will document the proportion of deliveries in which laboratory markers of congenital infection were present. Finally, we will investigate the associations of trimester of maternal infection in pregnancy, presence or absence of maternal symptoms of acute ZIKV infection, and previous flavivirus infections with adverse outcomes and with markers of congenital infection. Centre-specific estimates will be pooled using a two-stage approach.

Ethics and dissemination

Ethical approval was obtained at each centre. Findings will be presented at international conferences and published in peer-reviewed open access journals, and discussed with local public health officials and representatives of the national Ministries of Health, Pan American Health Organization, and World Health Organization involved with ZIKV prevention and control activities.

Keywords

Zika virus; pregnancy; vertical transmission; Congenital Zika Syndrome; microcephaly; meta-analysis

Article summary

Strengths and limitations of this study

- This will be a pooled analysis of data from 3 international consortia conducting prospective cohort studies of outcomes following ZIKV infection in pregnancy in 20 centres in 11 countries.

For peer review only - http://bmjopen.bmj.com/site/about/guidelines.xhtml

- Standardised definitions of outcomes will provide clarity about the absolute risks of adverse outcomes, which have not been reported consistently in prospective studies so far.
- These studies include a control group of women with no evidence of ZIKV infection in pregnancy, allowing improved estimation of the proportion of adverse events attributable to ZIKV in pregnancy.
- Inferences will be limited by: difficulties in distinguishing between women who did and who did not experience a ZIKV infection in pregnancy, due to the high frequency of mild and asymptomatic infections and the low sensitivity and specificity of diagnostic tools; low diagnostic sensitivity of markers of congenital infection; and outcome data that are not missing at random.

For peer review only

For peer review only - http://bmjopen.bmj.com/site/about/guidelines.xhtml

INTRODUCTION

Following the emergence of Zika virus (ZIKV) in Asia and the Pacific in 2013 (1) and the subsequent introduction to Brazil (2), clusters of neonates with severe neurological complications and microcephaly were observed across Latin America. Following recent experiences with the H1N1 influenza pandemic and Ebola outbreak in Western Africa, the need for coordinated international research on ZIKV was quickly recognized. In January 2016, before the World Health Organization (WHO) declared a Public Health Emergency of International Concern (3), the European Commission (EC) issued a funding call to set up a network in Latin America and the Caribbean with the aim of implementing and coordinating urgently required research, while simultaneously contributing to research capacity and preparedness for other emerging infectious diseases. Three consortia were funded: ZikaPLAN (4) (https://zikaplan.tghn.org/), ZIKAlliance (https://zikalliance.tghn.org/), and ZIKAction (http://zikaction.org/). All are multidisciplinary international collaborations with active investigations in epidemiology, virology, immunology, diagnostics, mathematical modelling, social science, and animal studies. Each consortium includes its own prospective cohort study of ZIKV in pregnancy and a shared work package that aims to ensure the harmonization of protocols and data sets in order to facilitate a pooled analysis of cohort data. The primary aim of the pooled analysis is to investigate the incidence of adverse outcomes of ZIKV infection in pregnancy, including "congenital infection, microcephaly, Zika congenital syndrome, and other sequelae of ZIKV infection".

The aim of this paper is to present a protocol for this pooled analysis. Data has been or is still being collected in multiple sites in 20 regional coordinating centres spread over 11 countries and regions across Latin America and the Caribbean. There are 15 ZIKAlliance centres: Sao Paulo, Rio de Janeiro, Recife and Belo Horizonte (Brazil); Valencia (Venezuela); Bucaramanga (Colombia); Guayaquil (Ecuador); Lima (Peru); Jalisco, Nayarit, Veracruz, Yucatan (Mexico); Santa Cruz de la Sierra (Bolivia); Havana (Cuba); Guadeloupe (French Territory of the Americas); 3 ZikaPLAN centres: Goiânia, Rio de Janeiro, Recife (Brazil); and 2 ZIKAction centres: Kingston (Jamaica) and Port-au-Prince (Haiti).

Several studies of ZIKV in pregnancy have recently been published. In registry-based studies (5–7), fetuses and newborns of women with confirmed infection in pregnancy have been reported to have "potentially Zika-related" adverse outcomes at rates of up to 15%, with higher rates in the first trimester. Registry-based studies are likely to over-estimate the risk of severe clinical manifestations and underestimate the risk of more mild clinical presentations because they recruit both prospectively ascertained ZIKV-infected pregnant women and women whose infection was recognized retrospectively

For peer review only - http://bmjopen.bmj.com/site/about/guidelines.xhtml

following the birth of an infant with congenital abnormalities. Prospective studies of congenital infection have variously reported 25% “severe” and 21% “mild to moderate” outcomes in French Guiana (8), and 27% adverse outcomes in Brazil (9). The specificity of these outcome definitions for ZIKV in pregnancy is not known as these studies did not include a control group of women with no ZIKV infection in pregnancy. In another Brazilian study, the risk of adverse outcomes was reported to be 46% in births to women with NAAT (Nucleic Acid Amplification Test)-confirmed ZIKV infection in pregnancy compared to 11.5% in NAAT-negative women (10). In a large prospective study based in the French Territories of the Americas, among infants born to women with NAAT-confirmed ZIKV infection, 7.0% presented with neurologic or ocular birth defects and 3.1% met the study’s criteria for Congenital Zika Syndrome (CZS) (14), which is characterized by several unique features (11). An important limitation in the comparison of the results of these different studies is the lack of a standard definition of CZS and of the clinical and diagnostic procedures used to evaluate these children, leading to possible misclassification of the outcomes studied.

The vertical transmission rate is the probability of congenital infection in births to women with infection in pregnancy. The rates reported so far, 26% (9) and 35% (8), are based on laboratory markers of congenital infection such as NAAT or IgM in the fetus or newborn. However, a prospective cohort retrospectively reconstructed from a register study estimated the vertical transmission rate to be only 9% (12). Comparison of these rates is difficult as different markers and different biological samples were used. In addition, although these tests are analytically sensitive and specific they have poor diagnostic sensitivity as markers of congenital infection. These markers were absent from serum in a high proportion of CZS cases (13) (14) and in newborns with other potentially ZIKV-related adverse outcomes born to women with confirmed ZIKV during pregnancy (8,9). Clearance of virus from amniotic fluid and fetal blood has been reported in cases of CZS, even when ZIKV is found in brain tissue post-mortem (15,16). It therefore appears that fetal infection may occur, causing profound damage, but clearing before delivery and leaving no discernable immunological trace in serum. Consequently, in this study we will document the prevalence of markers of congenital infection using uniform criteria, recognizing that this is an underestimate of the true vertical transmission rate.

Regarding effect modifiers, a number of studies have reported a higher incidence of congenital abnormalities following maternal infections in the first trimester (7,10–11,14). Maternal symptoms during acute ZIKV infection do not appear to be a risk factor for adverse outcomes (18). There is evidence of antibody dependent enhancement of ZIKV by dengue virus (DENV) antibody in animal

For peer review only - http://bmjopen.bmj.com/site/about/guidelines.xhtml

models (19), but it is unclear whether previous DENV infection or exposure to other flaviviruses has a protective, risk-enhancing, or null effect, in maternal or congenital infection in humans (20). It also remains to be established whether a previous ZIKV infection confers protective immunity. Little is currently known about risk factors for trans-placental transmission of ZIKV.

The analysis plan described here complements the recently published protocol of the Zika Virus Individual Participant Data Consortium (21), which will eventually include data from the three EC consortia as well as data from many other sources. Although the objectives of the protocols are similar, different methods are proposed, especially in relation to standardizing diagnostic results and outcomes.

In light of the unexplained heterogeneity in reported rates of adverse outcomes, and the variation in prevalence of markers of congenital infection, a pooled analysis of data from 20 centers following similar protocols with harmonized definitions of clinical and laboratory outcomes will provide important new information on outcomes of ZIKV in pregnancy.

OBJECTIVES OF THE JOINT ANALYSIS

1. To estimate the risk of adverse outcomes in the fetus, newborn, and child following maternal ZIKV infection in pregnancy, compared to outcomes in controls with no evidence of maternal infection in pregnancy.
2. To estimate the prevalence of markers of congenital infection in deliveries to women with ZIKV infection during pregnancy.
3. To assess the associations between trimester of maternal infection, presence or absence of maternal symptoms, and previous flavivirus infections with adverse outcomes and markers of congenital infection.

METHODS

Participants

Pregnant women were eligible only if their infection status during pregnancy (infected or not infected) was ascertained prior to the detection of adverse outcomes, or was not influenced by fetal examination or outcome on delivery. This definition is compatible with retrospective testing of previously collected maternal samples, after delivery. Although the unit of recruitment is the mother, the unit of analysis is the fetus, newborn, and infant; multiple births are sufficiently rare to be treated as independent observations (22).

For peer review only

Study design

Consenting women were screened in pregnancy for markers of ZIKV infection. Those in whom maternal infection in pregnancy (MIP) was suspected were followed with enhanced investigations. In ZIKAction and ZIKAlliance, all deliveries to these women, including fetal losses, stillbirths, and newborns were examined clinically and tested for markers of congenital infection. This testing was not routinely performed in ZikaPLAN. In all three cohorts, newborns were prospectively followed to identify any adverse outcomes that may develop later. A sample of newborns delivered to women with no evidence of infection in pregnancy served as an unexposed control group.

There were some differences between the protocols adopted by the three consortia in terms of how women were recruited into the study, and the choice and scheduling of tests and investigations (Supplementary Table S1). In ZIKAction and ZIKAlliance, women were recruited regardless of symptoms during pregnancy, although report of symptoms was recorded. In ZikaPLAN, only women with rash, a common sign of ZIKV infection, were recruited. Statistical analyses will therefore be stratified by whether the mother reported symptoms in pregnancy. Patients or the public were not involved in the protocol design.

Target parameters and terminology of vertical transmission studies

The logic of an idealised prospective study is shown in Table 1. The usual target parameters are the *vertical transmission rate*, which is the probability of congenital infection following MIP, (A+B) / (A+B+C+D); and the *rate of adverse outcomes in those with congenital infection,* A/(A+B). The definitions of "adverse outcomes", congenital infection, and MIP will be determined by a Joint Diagnostics Group and a Joint Endpoint Review Group, after the data have been assembled. Estimates of these parameters are standard in studies of vertical transmission of human immune-deficiency virus (HIV) (23,24), toxoplasmosis (25–27) and cytomegalovirus (CMV) (28). In studies of less specific outcomes, the event rate C/(C+D) in fetuses and newborns of women with MIP but in whom no congenital infection occurred (Control Group 1 in Table 1) forms a comparison group (29,30) representing the adverse event rate that is due to MIP in the absence of congenital infection.

However, because cases of congenital infection cannot be reliably identified by diagnostic tests, we can only estimate the prevalence of laboratory *markers of vertical infection* (Objective 2). Similarly, the "overall" (unconditional) adverse event rate is taken as the primary outcome for Objective 1; this includes all births to women with MIP, (A+C)/(A+B+C+D) (Table 1). All three consortia included a further control

For peer review only - http://bmjopen.bmj.com/site/about/guidelines.xhtml

group of births to unexposed women, those with no infection in pregnancy (Control Group 2 in Table 1). The adverse event rates in this group, E/(E+F), represents a baseline for comparison with the overall event rates in exposed women (31).

Although estimates of the vertical transmission rate are compromised, it may still be of interest to compare adverse outcome rates in the MIP with congenital infection group (i.e., A/(A+B)), the MIP without congenital infection group (i.e., C/(C+D)), and the No MIP group (i.e., E/(E+F)), as this may provide insight into whether adverse fetal outcomes are associated with MIP in the absence of demonstrable fetal infection.

In addition to the challenges associated with the laboratory definition of congenital infection, it is also difficult to discriminate between pregnancies with MIP and with No MIP as required in analyses based on Table 1. A positive NAAT result or seroconversion during pregnancy are sufficient to confirm MIP. However, even if tested *per protocol*, MIP may be missed due to the narrow window of detection of NAAT tests, perhaps as low as 14 days (32). Tests of recent infection, including IgM, IgG3 or avidity assays, may reflect infection during pregnancy, but may also be the result of infection prior to pregnancy (33), and immunologic cross-reactivity to DENV antibody may need to be ruled out (34). These tests therefore indicate suspected but not confirmed MIP. An IgG negative response in the woman or newborn at or shortly after delivery can be taken as suggestive of No MIP, although the dynamics of ZIKV IgG are not well documented. While a negative IgG is likely to be uncommon in some sites, as some women will have experienced a ZIKV infection prior to pregnancy, it lowers the probability that a ZIKV infection would have occurred in pregnancy. Taking all this together, MIP status will be characterised as “Confirmed”, “Suspected, “No Evidence of MIP" (i.e., all NAAT and IgM tests negative), and "No MIP" (i.e., all NAAT and IgM tests negative AND IgG negative at or shortly after delivery).

Definitions of variables

To support the pooled analyses, a Joint Diagnostics Group and a Joint Endpoint Review Group will be convened to agree on standardized case definitions. Supplementary Table S2 provides some provisional definitions for: MIP (Confirmed, Suspected, No Evidence of MIP, No MIP), Markers of congenital infection (present or absent), signs and symptoms compatible with Congenital Zika Syndrome (CZS), other potentially-Zika-related outcomes (OPZRO), and trimester of MIP. To the greatest extent possible, definitions will be harmonized across consortia and across centres within consortia.

For peer review only - http://bmjopen.bmj.com/site/about/guidelines.xhtml

An essential aspect of the definitions for MIP status used in the statistical analyses below is that they must be based exclusively on the *prospective* diagnostic testing. For example, although CZS and/or laboratory evidence of congenital infection in the newborn provides compelling evidence of MIP, this would not affect the MIP status as ascertained prospectively. Thus, we expect to observe some newborns with CZS and/or with laboratory markers of congenital infection delivered to women with Suspected MIP, or even those categorized as having No Evidence of MIP.

For peer review only

STATISTICAL ANALYSIS

Analysis of data from individual centres

Descriptive tables

We will produce descriptive tables providing a breakdown of the numbers in each centre with Confirmed MIP, Suspected MIP, No Evidence of MIP, and No MIP, and the type of evidence on which this is based (e.g., NAAT, seroconversion, tests of recent infection) (Supplementary Table S3). The similar Supplementary Table S4 will document numbers with signs or symptoms compatible with CZS and with markers of congenital infection, and the type of evidence on which this is based (e.g., NAAT, IgM).

Objective 1: Adverse Outcomes

Prospectively ascertained MIP status will be tabulated against overall (i.e., unconditional on congenital infection status) adverse outcomes (Table 2). Various risks can be estimated within each MIP category, including: the risk of signs and symptoms compatible with CZS, the risk of OPZRO, the risk of both combined, of individual signs and symptoms, or of signs and symptoms grouped in clinically (e.g., ophthalmologic defects) or embryologically meaningful ways. Outcomes may be binary (e.g., microcephaly) or continuous (e.g., head circumference), or multi-category (CZS-related outcomes, OPZRO, asymptomatic). As well as congenital anomalies, rates of standard outcomes, in the absence of congenital anomalies, will be documented, including: fetal loss, stillbirth, low birthweight, intra-uterine growth retardation (IUGR), and premature delivery.

For peer review only - http://bmjopen.bmj.com/site/about/guidelines.xhtml

The rate of adverse outcomes in the No MIP group represents the study-specific background rate of adverse outcomes (i.e., in the absence of MIP, Control Group 2), to be compared to rates in women with Confirmed MIP. Absolute risks will be estimated as well as risk ratios and risk differences.

Parallel sets of estimates will be calculated in the Suspected MIP and No Evidence of MIP groups, as particular adverse events indicate lack of diagnostic specificity and sensitivity in the testing protocol during pregnancy and are therefore informative regarding the effectiveness of the maternal testing protocol in each centre; for example, microcephaly in the No Evidence of MIP group would indicate a lack of sensitivity. These sets of estimates may be pooled, respectively, with adverse outcome risks in Confirmed MIP and No MIP groups in sensitivity analyses.

Objective 2: Markers of vertical transmission

Estimates of the prevalence of markers of congenital infection will be produced in all centres with available data for each MIP group except the No MIP group, as the definition of No MIP is not compatible with laboratory markers of congenital infection. For this purpose, the numerator will be the number with markers of congenital infection, and the denominator will be the sum of the numbers with and without markers of congenital infection. Separate estimates will be obtained for: laboratory markers, clinical markers, such as microcephaly, and combined laboratory and clinical markers (Table 3). Rates in the No Evidence of MIP group are of interest as they carry information about the diagnostic accuracy of the maternal testing protocol.

Objective 3: Effect of covariates

Separate estimates of the prevalence of adverse pregnancy outcomes, and the probability of laboratory markers of congenital infection, will be produced for each trimester of maternal infection, and by presence or absence of maternal symptoms.

Analyses of adverse event frequencies and of markers of congenital infection can be extended to include multiple covariates, using logistic regression. These might include potential *effect modifiers,* such as previous arbovirus infection or co-infection, or *confounding factors* such as socio-economic indicators likely to be associated with both arbovirus exposure and adverse outcomes. However, at the time of writing it is not known whether sufficient data will be available for regression analyses.

For peer review only - http://bmjopen.bmj.com/site/about/guidelines.xhtml

Secondary objectives

ZIKV infection in pregnancy could lead to adverse pregnancy and birth outcomes either following a congenital infection (cell A in Table 1) or in the absence of congenital infection (equally in cells A and C). Although absence of markers of congenital infection does not rule out congenital infection, we might still expect to observe more adverse outcomes in fetuses and newborns with laboratory markers of congenital infection if those outcomes are caused by congenital infection. By contrast, adverse outcomes that are the result of MIP in the absence of congenital infection (Cell E in Table 1) should occur equally with or without laboratory markers of congenital infection. Based on literature on other infections in pregnancy, including dengue virus (35), adverse outcomes associated with MIP in the absence of congenital infection potentially include: fetal loss, stillbirth, prematurity, IUGR, and low birthweight for gestational age (36). The analysis would be based on a tabulation of presence or absence of neonatal symptoms, or sets of symptoms (Supplementary Table S5), and would be stratified by trimester of maternal infection, as this is likely to be associated with the presence of markers of congenital infection and with adverse outcomes.

Missing Covariates

All analyses will be conducted on a "complete case" basis, in the first instance. Methods for handling missing covariates, such as imputation (37), will be considered after the extent and patterns of missing data have been explored.

Combining data across centres

The above analyses will generate a series of centre-specific estimates of proportions, relative risks comparing MIP and No MIP groups, risk-differences, and means of continuous variables, stratified by trimester and maternal clinical presentation. If logistic regression is used to examine effect-modifiers and confounders, further estimates of interaction terms or adjusted estimates can also be produced.

All these estimates can be combined across centres using fixed or random effects models in a "two-stage" meta-analysis. Bayesian Markov chain Monte Carlo methods will be used as this will facilitate the use of exact binomial and multinomial likelihoods, which have a better performance with low and zero cells

For peer review only - http://bmjopen.bmj.com/site/about/guidelines.xhtml

counts. Vague priors will be employed. Centre-specific random effect estimates will be sampled from beta distributions for binomial outcomes data, Dirichlet distributions for multinomial data, and normal distributions for continuous data.

In combining estimates from different centres, we will take account of the fact that in ZikaPLAN only women with rash were recruited, so that women with no ZIKV infection may have experienced other exanthematic infections (10), including arbovirus infections such as dengue and chikungunya, which may themselves be associated with adverse outcomes (38,39).

Depending on the results of two-stage analyses, and the completeness of covariate data, an individual patient data one-stage meta-analyses will be considered for each objective, as a secondary or sensitivity analysis, with centre as an additional fixed "intercept" term.

Sensitivity analyses

Definitions of MIP status, ZIKV-related outcomes, and laboratory markers of congenital infection

We will report differences in adverse event rates between Confirmed MIP and Suspected MIP, and between No Evidence of MIP and No MIP. If the differences are small, we will produce results pooling these categories as a sensitivity analysis.

Further, the No Evidence of MIP category can be subdivided into women who were tested *per protocol* and those who may have been tested less completely. The impact of compliance with protocols will be explored, as it is expected to impact on the proportion of women with MIP who are classified as "No evidence of MIP" and, hence, on the probability of observing adverse outcomes in this group. Similarly, we will conduct sensitivity analyses around the definitions of Confirmed and Suspected MIP on advice from the Joint Diagnostics Group.

Alternative sets of estimates will be generated using alternative criteria for CZS-related outcomes and OPZRO, that are more, or less, specific for ZIKV in pregnancy. Similarly, we will explore the impact of varying the laboratory criteria for congenital infection on the advice of the Joint Diagnostics Group.

For peer review only - http://bmjopen.bmj.com/site/about/guidelines.xhtml

Independent ascertainment of outcomes

A critical requirement of all these analyses is that the ascertainment of markers of congenital infection status and clinical outcomes in the fetuses and newborns, and developing infant are all independent of each other, and also independent of MIP status. For example, ideally the same laboratory testing for congenital infection is carried out regardless of whether the pregnancy outcome is a fetal loss, termination of pregnancy, stillbirth, a case of CZS, or an apparently healthy asymptomatic infant.

These assumptions are difficult, and in certain respects (e.g., first trimester fetal loss and terminations) not possible, to fully implement in practice. To address these inevitable limitations in the analysis, which are expected to impact more on objective 2 than on objective 1, we will carry out exploratory analyses aimed at detecting potential deviations from protocol. For example, the distribution of trimester of MIP should not be associated with prospectively ascertained MIP status. Guided by the results, we will carry out sensitivity analyses that make a range of assumptions about the distribution of missing data, especially data on markers of congenital infection. A series of scenarios will be examined to assess robustness of results to inherent and/or unplanned deviations from the ideal protocols required for unbiased estimation of the target parameters.

DISCUSSION

Risks of CZS and other adverse birth outcomes of ZIKV infection in pregnancy can only be assessed through studies that recruit women whose infection status is prospectively ascertained, or, if retrospective, ascertainment is independent of outcomes. However, reported risks of adverse outcomes even from prospective studies have been highly variable (8–10,17), as have vertical transmission rates based on laboratory markers of congenital ZIKV infection (8,9,12). An important role for joint analyses of multiple studies is to explore whether this heterogeneity in outcomes can be explained by individual or study-level covariates. To do this, it is essential that incidental sources of variation, such as those arising from differences in outcome reporting or diagnostic testing, are controlled or eliminated as much as possible. One of the most difficult sources of variation between consortia, and between sites within consortia, lies in diagnosis of maternal infection. Our approach is to have an Expert Diagnostics Group produce a harmonised classification of Confirmed MIP, Suspected MIP, No Evidence of MIP, and No MIP, and to compute a range of estimates of the relative effect of maternal infection on outcomes, grouping these in different ways. An analysis based on the Confirmed MIP and No MIP groups alone would be expected to

For peer review only - http://bmjopen.bmj.com/site/about/guidelines.xhtml

generate the largest estimates of relative effect, because both poor sensitivity and poor specificity will tend to bias effect estimates towards the null. An alternative proposal (21) is to treat test sensitivity and specificity as study-level covariates in a meta-regression. Other statistical methods may have been developed by the time the data becomes available for these analyses. Whatever form of analysis is adopted, a standardised pooled analysis from three large consortia comprising 20 centres will provide valuable information about these parameters, which will assist in framing a public health response and advice to women who might be exposed in future. It may also throw light on pathologic mechanisms leading to adverse outcomes, which could help in the development of therapeutic or prophylactic interventions.

For peer review only

For peer review only - http://bmjopen.bmj.com/site/about/guidelines.xhtml

Acknowledgements The authors express their thanks to Tom Byrne for organising the references.

Collaborators Other members of the EC Zika Consortia Vertical Transmission Study Group are: Joshua ANZINGER (Department of Microbiology, Virology Section, University of the West Indies, Mona Campus, Kingston, Jamaica; Global Virus Network, Baltimore, MD, USA), Heather BAILEY (Institute for Global Health, University College London, UK) Valery E M BEAU DE ROCHARS (Emerging Pathogens Institute, University of Florida, Gainesville, Florida, United States of America), Luana DAMASCENO (Evandro Chagas National Institute of Infectious Diseases, Fundação Oswaldo Cruz, Rio de Janeiro, Brazil), Maria de Fatima Pessoa Militão DE ALBUQUERQUE (Instituto Aggeu Magalhães, Fundação Oswaldo Cruz, Recife, Pernambuco, Brazil), Rafael F. O. FRANÇA (Instituto Aggeu Magalhães, Fundação Oswaldo Cruz, Recife, Pernambuco, Brazil), Hugo Lopez GATELL RAMIREZ (Centro de Investigación sobre Enfermedades Infecciosas, Instituto Nacional de Salud Pública, Cuernavaca, Mexico), Adriana GOMEZ (Universidad Industrial de Santander, Bucaramanga, Colombia), Fabiana GORDON (Population Health Sciences, Bristol Medical School, University of Bristol, Bristol, United Kingdom), Maria G GUZMAN (Instituto de Medicina Tropical Pedro Kourí, La Havana, Cuba), Daniel LANG (Fondazione Penta Onlus, São Paulo, São Paulo, Brazil), Rigan LOUIS (Emerging Pathogens Institute, University of Florida, Gainesville, Florida, United States of America), Anyela Lozano (Universidad Industrial de Santander, Bucaramanga, Colombia), Eric MARTINEZ (Instituto de Medicina Tropical Pedro Kourí, La Havana, Cuba), Philippe MAYAUD (Department of Clinical Research, London School of Hygiene and Tropical Medicine, London, UK), Ricard ORTIZ SERRANO (Universidad Autónoma de Bucaramanga, Bucaramanga, Colombia), Silvia P. SALGADO (Instituto Nacional de Salud Pública e Investigación, Guyaquil, Ecuador), Aluisio Augusto Cotrim SEGURADO (Department of Infectious and Parasitic Diseases and Instituto de Medicina Tropical, Faculdade de Medicina da Universidade de Sao Paulo, Sao Paulo, Brazil), Zilton F. M. VASCONCELOS (Instituto Fernandes Figueira – Fundação Oswaldo Cruz, Rio de Janeiro, Rio de Janeiro, Brazil), Isabelle Freire Tabosa VIANA (Instituto Aggeu Magalhães, Fundação Oswaldo Cruz, Recife, Pernambuco, Brazil), Luis Angel VILLAR (Universidad Industrial de Santander, Bucaramanga, Colombia)

Author Contributions

The paper was drafted by AEA with the assistance of EBB and NA, following discussions with a steering group that included CT, TJ, KR and RAAX. The draft was revised based on comments from CT, DB, ASA,

For peer review only - http://bmjopen.bmj.com/site/about/guidelines.xhtml

TJ, KR, MP, DBM, RAAX, PM, MCM, KR, ATH. Comments were then solicited from members of the EC Zika Consortia Vertical Transmission Study Group collaborators, and a revision was drafted by AEA, EBB and NA. CG, XdL and AW-S are equal last authors and the consortia PIs. The final draft was agreed by all authors.

Funding European Union Directorate-General for Research grant numbers 734857 (ZIKAction), 734548 (ZIKAlliance), 734584 (ZikaPLAN), Horizon 2020 programme of the European Commission. EBB was also supported by Wellcome Trust & the UK's Department for International Development (205377/Z/16/Z to LCR; https://wellcome.ac.uk/).

Patient consent for publication Not required

Competing interests

All authors have completed the ICMJE uniform disclosure form. The following authors report grants from the European Commission (AET, EB, NA, DB, TJ, KDR, CT, CDC, ASA, JGM, CG, XL, AWS); EBB reports funding from by Wellcome Trust & the UK's Department for International Development; MMT reports grants from Conselho Nacional de Desenvolvimento Cientifico e Tecnologico, Brazil and from Fundacao de Amparo a Pesquisa do EStado de MInas Gerais (FAPEMIG, Brazil), during the conduct of the study; MK has a patent on zika diagnostics pending.

Data statement section Not applicable

For peer review only - http://bmjopen.bmj.com/site/about/guidelines.xhtml

TABLES

Table 1. Design of an idealized prospective vertical transmission study. The vertical transmission rate is estimated by (A+B)/(A+B+C+D). The rate of adverse outcomes conditional on congenital infection is A/(A+B). This can be compared with the rate of adverse outcomes in newborns with no congenital infection, C/(C+D), who form a control group (Control group 1) to account for potential confounders associated with maternal infection.

An overall, non-conditional estimate of the adverse event rate is (A+C)/(A+B+C+D).

Follow-up of births to women with no MIP creates a second control group (Control Group 2), in which the rate of adverse outcomes, E/(E+F), can be compared to the rate in births with no congenital infection to women with MIP. Estimated effects of MIP based on Control Group 2 are vulnerable to confounding by factors associated with MIP. Some cells are set to zero as there can be no congenital infection without MIP.

			**Maternal Infection Status**	
			MIP	No MIP
**Congenital Infection Status**	**congenital infection**	Adverse outcomes	A	0
		No Adverse outcomes	B	0
	**No congenital infection**	Adverse outcomes	C	E
		No Adverse outcomes	D	F

For peer review only

For peer review only - http://bmjopen.bmj.com/site/about/guidelines.xhtml

Table 2. Scheme for a generic analysis of risks of adverse outcomes by prospectively ascertained Maternal Infection in Pregnancy (MIP) status. Presence or absence of fetal and neonatal signs or symptoms (e.g., microcephaly, brain calcifications, arthrogryposis) and other Potentially Zika-related outcomes (e.g., fetal loss) in the different MIP groups will be compared.

**CENTRE**	**Maternal Infection in Pregnancy status**			
	Confirmed	Suspected	No Evidence of MIP	No MIP
**Symptom 1**				
**Symptom 2**				
**Symptom 3**				
**:**				
**No Symptoms**				
**TOTAL**				

Table 3. Scheme for generic analysis of markers of congenital infection by prospectively ascertained Maternal Infection in Pregnancy (MIP) status. Markers of congenital infection may include laboratory markers, clinical markers, such as microcephaly, or both combined.

**CENTRE**	**Maternal Infection in Pregnancy status**			
	Confirmed	Suspected	No Evidence of MIP	No MIP
**Markers of congenital infection present**				
**Markers of congenital infection absent**				
**Not tested**				
**TOTAL**				

For peer review only - http://bmjopen.bmj.com/site/about/guidelines.xhtml

References

1. Roth A, Mercier A, Lepers C, Hoy D, Duituturaga S, Benyon E, et al. Concurrent outbreaks of dengue, chikungunya and zika virus infections null An unprecedented epidemic wave of mosquito-borne viruses in the Pacific 2012null2014. Eurosurveillance. 2014;19(41):1–8.

2. Campos GS, Bandeira AC, Sardi SI. Zika Virus Outbreak, Bahia, Brazil. Emerg Infect Dis. 2015;21(10):1885–6.

3. Gulland A. Zika virus is a global public health emergency, declares WHO. BMJ. 2016;352(February):i657.

4. Wilder-Smith A, Preet R, Renhorn KE, Ximenes RA, Rodrigues LC, Solomon T, et al. ZikaPLAN: Zika Preparedness Latin American Network. Glob Health Action. 2017;10(1):1398485.

5. Reynolds M, Jones A, Petersen E, Lee E, Rice M, Bingham A. Vital Signs: Update on Zika Virus–Associated Birth Defects and Evaluation of All U.S. Infants with Congenital Zika Virus Exposure — U.S. Zika Pregnancy Registry, 2016. Morb Mortal Wkly Rep. 2017;66(13):366–73.

6. Shapiro-Mendoza CK, Rice ME, Galang RR, Fulton AC, VanMaldeghem K, Prado MV, et al. Pregnancy Outcomes After Maternal Zika Virus Infection During Pregnancy — U.S. Territories, January 1, 2016–April 25, 2017. MMWR Morb Mortal Wkly Rep. 2017;66(23):615–21.

7. Honein MA, Dawson AL, Petersen EE, Jones AM, Lee EH, Yazdy MM, et al. Birth defects among fetuses and infants of US women with evidence of possible zika virus infection during pregnancy. JAMA - J Am Med Assoc. 2017;317(1):59–68.

8. Pomar L, Vouga M, Lambert V, Pomar C, Hcini N, Jolivet A, et al. Maternal-fetal transmission and adverse perinatal outcomes in pregnant women infected with Zika virus: prospective cohort study in French Guiana. Bmj. 2018;k4431.

9. Nogueira ML, Nery Júnior NRR, Estofolete CF, Bernardes Terzian AC, Guimarães GF, Zini N, et al. Adverse birth outcomes associated with Zika virus exposure during pregnancy in São José do Rio Preto, Brazil. Clin Microbiol Infect. 2018 Jun;24(6):646–52.

10. Brasil P, Pereira J, Moreira E, Riberio Nogueira RM, Damasceno L, Wakimoto M, et al. Zika Virus Infection in Pregnant Women in Rio de Janeiro. N Engl J Med. 2016;56(11):1024–5.

11. Moore CA, Staples JE, Dobyns WB, Pessoa A, Ventura C V., Da Fonseca EB, et al. Characterizing the pattern of anomalies in congenital zika syndrome for pediatric clinicians. JAMA Pediatr. 2017;171(3):288–95.

12. Conners EE, Lee EH, Thompson CN, Mcgibbon E, Rakeman JL, Iwamoto M, et al. Zika Virus Infection Among PregnantWomen and Their Neonates in NewYork City, January 2016–June 2017. Obs Gynecol. 2018;132:487–95.

13. van der Linden V, Pessoa A, Dobyns W, Barkovich AJ, Júnior H van der L, Filho ELR, et al. Description of 13 Infants Born During October 2015–January 2016 With Congenital Zika Virus Infection Without Microcephaly at Birth — Brazil. Morb Mortal Wkly Rep. 2016;65(47):1343–8.

14. de Araújo TVB, Ximenes RA de A, Miranda-Filho D de B, Souza WV, Montarroyos UR, de Melo APL, et al. Association between microcephaly, Zika virus infection, and other risk factors in Brazil: final report of a case-control study. Lancet Infect Dis. 2018 Mar;18(3):328–36.

15. Schaub B, Vouga M, Najioullah F, Gueneret M, Monthieux A, Harte C, et al. Analysis of blood from Zika virus-infected fetuses: a prospective case series. Lancet Infect Dis. 2017;17(5):520–7.

16. Rodó C, Suy A, Sulleiro E, Soriano-Arandes A, Antón A, García-Ruiz I, et al. In utero negativization of Zika virus in a foetus with serious central nervous system abnormalities. Clin Microbiol Infect. 2018;24(5):549.e1-549.e3.

17. Hoen B, Schaub B, Funk AL, Ardillon V, Boullard M, Cabié A, et al. Pregnancy Outcomes after ZIKV Infection in French Territories in the Americas. N Engl J Med. 2018;378(11):985–94.

For peer review only - http://bmjopen.bmj.com/site/about/guidelines.xhtml

18. Paixao ES, Leong WY, Rodrigues LC, Wilder-Smith A. Asymptomatic prenatal Zika virus infection and congenital Zika syndrome. Open Forum Infect Dis. 2018;5(4):1–5.
19. Bardina S V., Bunduc P, Tripathi S, Duehr J, Frere JJ, Brown JA, et al. Enhancement of Zika virus pathogenesis by preexisting antiflavivirus immunity. Science (80-). 2017;356(6334):175–80.
20. Masel J, McCracken MK, Gleeson T, Morrison B, Rutherford G, Imrie A, et al. Does prior dengue virus exposure worsen clinical outcomes of Zika virus infection? A systematic review, pooled analysis and lessons learned. PLoS Negl Trop Dis. 2019;13(1):1–14.
21. Wilder-Smith A, Wei Y, Araújo TVB de, VanKerkhove M, Turchi Martelli CM, Turchi MD, et al. Understanding the relation between Zika virus infection during pregnancy and adverse fetal, infant and child outcomes: a protocol for a systematic review and individual participant data meta-analysis of longitudinal studies of pregnant women and their inf. BMJ Open. 2019;9(6):e026092.
22. Thorne C, Malyuta R, Semenenko I, Pilipenko T, Patel D, Bunders M, et al. The mother-to-child HIV transmission epidemic in Europe: Evolving in the East and established in the West. Aids. 2006;20(10):1419–27.
23. Mandelbrot, Mayaux, Bongain, Berrebi, Moudoub-Jeanpetit, Bénifla, et al. Obstetric factors and mother-to-child transmission of human immunodeficiency virus type 1: The French perinatal cohorts. Am J Obstet Gynecol. 1996;175(3):661–7.
24. Dunn DT, Ades AE. Estimating the HIV Vertical Transmission Rate and the Pediatric AIDS Incubation Period from Prospective Data AU. J Am Stat Assoc. 1996 Sep 1;91(435):935–43.
25. Gras L, Gilbert RE, Ades AE, Dunn DT. Effect of prenatal treatment on the risk of intracranial and ocular lesions in children with congenital toxoplasmosis. Int J Epidemiol. 2001;30(6):1309–13.
26. Gilbert RE, Gras L, Wallon M, Peyron F, Ades AE, Dunn DT. Effect of prenatal treatment on mother to child transmission of Toxoplasma gondii: Retrospective cohort study of 554 mother-child pairs in Lyon, France. Int J Epidemiol. 2001;30(6):1303–8.
27. The SYROCOT (Systematic Review on Congenital Toxoplasmosis) study group. Eff ectiveness of prenatal treatment for congenital toxoplasmosis: a meta-analysis of individual patients' data. Lancet. 2007;369(9556):115–22.
28. Stagno S, Pass RF, Dworsky ME, Henderson RE, Moore EG, Walton PD, et al. Congenital Cytomegalovirus Infection: The relative importance of primary and recurrent maternal infection. N Engl J Med. 1982 Apr;306(16):945–9.
29. Townsend CL, Forsgren M, Ahlfors K, Ivarsson SA, Tookey PA, Peckham CS. Long-term outcomes of congenital cytomegalovirus infection in Sweden and the United Kingdom. Clin Infect Dis. 2013;56(9):1232–9.
30. Preece PM, Blount JM, Glover J, Fletcher GM, Peckham CS, Griffiths PD. The consequences of primary cytomegalovirus infection in pregnancy. Arch Dis Child. 1983;58(12):970–5.
31. Li XL, Wei HX, Zhang H, Peng HJ, Lindsay DS. A meta analysis on risks of adverse pregnancy outcomes in Toxoplasma gondii infection. PLoS One. 2014;9(5):1–12.
32. Paz-Bailey G, Rosenberg ES, Doyle K, Munoz-Jordan J, Santiago GA, Klein L, et al. Persistence of Zika Virus in Body Fluids — Final Report. N Engl J Med. 2018;NEJMoa1613108.
33. Centers for Disease Control and Prevention. Prolonged IgM Antibody Response in People Infected with Zika Virus : Implications for Interpreting Serologic Testing Results for Pregnant. 2017;5–7.
34. Rabe IB, Staples JE, Villanueva J, Hummel KB, Johnson JA, Rose L, et al. Interim Guidance for Interpretation of Zika Virus Antibody Test Results. MMWR Morb Mortal Wkly Rep. 2016;65(21):543–6.
35. Paixão ES, Teixeira MG, Costa M da CN, Barreto ML, Rodrigues LC. Symptomatic Dengue during Pregnancy and Congenital Neurologic Malformations. Emerg Infect Dis. 2018 Sep;24(9):1748–50.

For peer review only - http://bmjopen.bmj.com/site/about/guidelines.xhtml

36. Cardenas I, Means RE, Aldo P, Koga K, Lang SM, Booth C, et al. Viral Infection of the Placenta Leads to Fetal Inflammation and Sensitization to Bacterial Products Predisposing to Preterm Labor. J Immunol. 2010;185(2):1248–57.
37. JL. S. Multiple imputation: a primer. Stat Methods Med Res. 1999;8(1):3–15.
38. Basurko C, Matheus S, Hildéral H, Everhard S, Restrepo M, Cuadro-Alvarez E, et al. Estimating the risk of vertical transmission of dengue: A prospective study. Am J Trop Med Hyg. 2018;98(6):1826–32.
39. Contopoulos-Ioannidis D, Newman-Lindsay S, Chow C, LaBeaud AD. Mother-to-child transmission of Chikungunya virus: A systematic review and meta-analysis. PLoS Negl Trop Dis. 2018;12(6):1–20.

For peer review only

For peer review only - http://bmjopen.bmj.com/site/about/guidelines.xhtml

SUPPLEMENTARY MATERIALS

Supplementary Table S1. Schedule of tests and examinations in pregnancy, at delivery, and during paediatric follow-up, in each consortium.

Consortium	Mother	Fetus	Newborn and neonate	Pediatric follow-up
ZIKAlliance	ZIKV serology IgG & IgM, NAAT blood and urine, at booking and every 4w.	Laboratory, pathology examination of fetal loss and stillbirth.	NAAT on placenta.<br><br>Clinical examination of newborn.<br><br>Newborn testing: NAAT, IgM cord blood, urine; TORCH, HIV	Clinical examination<br><br>NAAT blood urine; IgM, IgG blood at 4w, 4m, 12m, 24m.<br><br>Transcranial U/S at 4w. Hearing, eye, neurodevelopmental assessments
ZIKAction	IgM, IgG serology booking, 20, 28, Delivery, and at pediatric follow-up<br><br>NAAT testing, and additional U/S investigations if evidence of infection	Laboratory, pathology examination of fetal loss and stillbirth.	Clinical examination of newborn<br><br>Newborn testing: IgM, IgG (serum; CSF only if clinically indicated), urine, saliva.<br><br>NAAT, U/S and ophthalmology investigations if evidence of infection	Paediatric testing:<br><br>IgG, IgM (serum, urine, saliva) and clinical examination at: 4w, 4m, 9m, 12m, 18m and 24m. NAAT if evidence on infection<br><br>Neurodevelopmental assessments
ZikaPLAN	NAAT and serologic testing (PRNT, IgM, IgG3) when symptoms reported and at up to three follow-up visits.<br><br>TORCH, DENV, and CHIKV testing	Not routinely tested.	Clinical examination in early infancy.<br><br>NAAT testing in the neonatal period in some centres	Clinical examination of the child by specialists at 3, 6, 12, 18, 24, 36, and 48m<br><br>Neurodevelopmental assessments

CSF, Cerebro-spinal fluid; DENV, Dengue virus; CHIKV, Chikungunya virus; HIV, Human Immunodeficiency virus; Ig, Immunoglobulin; NAAT, Nucleic Acid Amplification Test; PRNT, Plaque Reduction Neutralisation Test; TORCH, Toxoplasmosis, Other, Rubella, Cytomegalovirus, Herpes; U/S, Ultrasound; ZIKV, Zika virus

For peer review only

For peer review only - http://bmjopen.bmj.com/site/about/guidelines.xhtml

Supplementary Table S2. Illustrative and approximate definitions of key explanatory and outcome variables. The final definitions will be determined by the Joint Diagnostics Group and Joint End-point Review Group.

Maternal Symptoms	For example: rash, fever, headache, joint pain
Maternal Infection in Pregnancy (MIP) status, established only on the basis of maternal testing	Confirmed: NAAT, Seroconversion<br>Suspected: Tests of recent infection, including: IgM, IgG3, avidity<br>No Evidence of MIP: Maternal testing protocol was followed, but none of the above were positive.<br>No MIP: As above but with IgG negative at or near time of delivery
Markers of Congenital Infection (CI)	Present: NAAT or IgM any time in first 7 days;<br>Absent: all other findings
Most likely trimester of onset of maternal infection	The highest available from the following hierarchy:<br>• Date of first NAAT positive test minus average duration of viremia<br>• Seroconversion: Midpoint between last serological negative and last positive<br>• Tests of recent infection: Date of first positive
Signs and symptoms compatible with Congenital Zika Syndrome (CZS)	A definition will be prepared by the Joint End-point Review Group, based on best evidence available.
Other Potentially Zika-related Outcomes (OPZRO)	A definition will be prepared by the Joint End-point Review Group, based on best evidence available.

Ig, Immunoglobulin; NAAT, Nucleic Acid Amplification Test.

For peer review only

Supplementary Table S3. Prospectively ascertained Maternal Infection in Pregnancy (MIP) status and evidence on which it is based. NB: Data from the same woman can appear in more than one of the last four columns

Centre	Total women	Prospectively ascertained MIP status				Evidence for Confirmed or Suspected MIP			
		Confirmed MIP	Suspected MIP	No Evidence of MIP	No MIP	NAAT	Sero-conversion	IgM/ IgG3	PRNT
1									
2									
…									
etc									

Ig, Immunoglobulin; NAAT, Nucleic Acid Amplification Test; PRNT, Plaque Reduction Neutralisation Test.

Supplementary Table S4. Summary of evidence on laboratory and clinical markers of congenital infection (CI) and evidence on which it is based. NB: Data from the same fetus/newborn can appear in more than one column

Centre	Total number fetus / newborn	Markers of congenital infection			Evidence on congenital infection				
		Any marker of congenital infection	Laboratory markers of congenital infection	No markers of congenital infection	NAAT	IgM	Signs and symptoms compatible with CZS	Microcephaly	Other potentially Zika-related Symptoms
1									
2									
…									
etc									

CZS, Symptoms compatible with Congenital Zika Syndrome; IgM/G, Immunoglobulin M/G; NAAT, Nucleic Acid Amplification Test; OPZRO, Other potentially Zika-related symptoms; PRNT, Plaque Reduction Neutralisation Test.

For peer review only - http://bmjopen.bmj.com/site/about/guidelines.xhtml

Supplementary Table S5. Distributions of signs and symptoms by laboratory markers of congenital infection and prospectively ascertained maternal infection in pregnancy (MIP) status**.** The breakdown shown is an example. Other examples might be "Termination of pregnancy, Pregnancy loss, Stillbirth, Livebirth"; or "Sensorineural hearing loss (SNHL), No SNHL "

**CENTRE**			
**Sign or symptom in fetus, newborn, or infant**	**MIP with markers of congenital infection**	**MIP with no markers of congenital infection**	**No MIP**
Signs and symptoms compatible with Congenital Zika Syndrome (CZS)			
Other potentially Zika-related outcomes (OPZRO)			
Asymptomatic			
**TOTAL = 100%**			

For peer review only

BMJ Open

Zika virus infection in pregnancy: A protocol for the joint analysis of the prospective cohort studies of the ZIKAlliance, ZikaPLAN and ZIKAction consortia

Journal:	*BMJ Open*
Manuscript ID	bmjopen-2019-035307.R1
Article Type:	Protocol
Date Submitted by the Author:	20-Jul-2020
Complete List of Authors:	Ades, A E; University of Bristol<br>Brickley, Elizabeth B.; London School of Hygiene and Tropical Medicine, Department of Infectious Disease Epidemiology<br>Alexander, Neal; London School of Hygiene and Tropical Medicine Faculty of Epidemiology and Population Health<br>Brown, David; 4. Fundacão Oswaldo Cruz, Flavivirus Reference Laboratory<br>Jaenisch, Thomas ; UniversitatsKlinikum Heidelberg, Department of Infectious Diseases, Section Clinical Tropical Medicine;<br>Miranda-Filho, Demócrito de Barros; University of Pernambuco, Faculty of Medical Sciences<br>Pohl, Moritz; 7. Institute of Medical Biometry and Informatics, University of Heidelberg<br>Rosenberger, Kerstin Daniela; UniversitatsKlinikum Heidelberg, Department of Infectious Diseases, Section Clinical Tropical Medicine<br>Soriano-Arandes, Antoni; University Hospital Vall d'Hebron, Paediatric Infectious Diseases and Immunodeficiencies Unit, Department of Pediatrics<br>Thorne, Claire; University College London, Great Ormond Street Institute of Child Health<br>Arraes de Alencar Ximenes, Ricardo; Federal University of Pernambuco, Department of Tropical Medicine<br>Velho Barreto de Araújo, Thalia; Universidade Federal de Pernambuco, Department of Social Medicine<br>Avelino-Silva, Vivian ; Faculdade de Medicina da Universidade de Sao Paulo, Department of Infectious and Parasitic Diseases<br>Bethencourt, Sarah ; Universidad de Carabobo, Facultad de Ciencias de la Salud<br>Borja-Aburto, Victor; Mexican Institute of Social Security<br>Brasil, Patrícia ; Fundacao Oswaldo Cruz, Instituto de pesquisa Clínica Evandro Chagas<br>Christie, C. D. C.; University of the West Indies at Mona, Department of Child and Adolescent Health<br>DE SOUZA, Wayner; Instituto Aggeu Magalhães, Fundação Oswaldo Cruz<br>GOTUZZO H., Jose Eduardo ; Universidad Peruana Cayetano Heredia Instituto de Medicina Tropical Alexander von Humboldt<br>Hoen, Bruno; INSERM Centre d'Investigation Clinique 1424, Centre Hospitalier Universitaire de Pointe-à-Pitre; Centre Hospitalier Universitaire de Pointe-à-Pitre

	Koopmans, M; Erasmus Universiteit Rotterdam, Department of Viroscience<br>Turchi Martelli, Celina Maria; Oswaldo Cruz Foundation , Department of Collective Health, Institute Aggeu Magalhães (CPqAM)<br>Teixeira, Mauro; Universidade Federal de Minas Gerais<br>Marques, Ernesto; Instituto Aggeu Magalhães, Fundação Oswaldo Cruz; University of Pittsburgh, Department of Infectious Diseases and Microbiology<br>Miranda Montoya, Maria Consuelo ; Universidad Industrial de Santander, Facultad de Salud ; Universidad Industrial de Santander, Centro de >Investigaciones Epidemiológicas<br>Ramos Montarroyos, Ulisses; 26. Instituto de Ciências Biológicas, Universidade de Pernambuco<br>Moreira, Maria ; Figueira National Institute for Women’s, Children’s and Adolescents Health, Oswaldo Cruz Foundation<br>Morris, J; University of Florida, Emerging Pathogens Institute<br>Rockx, Barry; Erasmus Universiteit Rotterdam, Department of Viroscience<br>SABA VILLARROEL, Paola Mariela ; Centro Nacional de Enfermedades Tropicales, Santa Cruz de la Sierra<br>SORIA SEGARRA, Carmen ; Universidad Católica de Santiago de Guayaquil; SOSECALI C., Ltda<br>Tami, Adriana; University Medical Center Groningen, Department of Medical Microbiology and Infection Prevention<br>Turchi, Marília Dalva; Federal University of Goias, Institute of Tropical Pathology and Public Health<br>Giaquinto, Carlo; University of Padova Faculty of Medicine and Surgery, Department of Woman’s and Child’s Health<br>de-Lamballerie, Xavier; Aix-Marseille Université Institut Universitaire de Technologie d'Aix-en-Provence<br>Wilder-Smith, Annelies; Umeå University

**Primary Subject Heading**:	Global health
Secondary Subject Heading:	Epidemiology, Infectious diseases
Keywords:	EPIDEMIOLOGY, Public health < INFECTIOUS DISEASES, Paediatric infectious disease & immunisation < PAEDIATRICS

SCHOLARONE™ Manuscripts

I, the Submitting Author has the right to grant and does grant on behalf of all authors of the Work (as defined in the below author licence), an exclusive licence and/or a non-exclusive licence for contributions from authors who are: i) UK Crown employees; ii) where BMJ has agreed a CC-BY licence shall apply, and/or iii) in accordance with the terms applicable for US Federal Government officers or employees acting as part of their official duties; on a worldwide, perpetual, irrevocable, royalty-free basis to BMJ Publishing Group Ltd ("BMJ") its licensees and where the relevant Journal is co-owned by BMJ to the co-owners of the Journal, to publish the Work in this journal and any other BMJ products and to exploit all rights, as set out in our licence.

The Submitting Author accepts and understands that any supply made under these terms is made by BMJ to the Submitting Author unless you are acting as an employee on behalf of your employer or a postgraduate student of an affiliated institution which is paying any applicable article publishing charge ("APC") for Open Access articles. Where the Submitting Author wishes to make the Work available on an Open Access basis (and intends to pay the relevant APC), the terms of reuse of such Open Access shall be governed by a Creative Commons licence – details of these licences and which Creative Commons licence will apply to this Work are set out in our licence referred to above.

Other than as permitted in any relevant BMJ Author's Self Archiving Policies, I confirm this Work has not been accepted for publication elsewhere, is not being considered for publication elsewhere and does not duplicate material already published. I confirm all authors consent to publication of this Work and authorise the granting of this licence.

For peer review only

Zika virus infection in pregnancy: A protocol for the joint analysis of the prospective cohort studies of the ZIKAlliance, ZikaPLAN and ZIKAction consortia

A. E. ADES[1], Elizabeth B. BRICKLEY[2], Neal ALEXANDER[3], David BROWN[4], Thomas JAENISCH[5], Demócrito de Barros MIRANDA-FILHO[6], Moritz POHL[7], Kerstin D. ROSENBERGER[5], Antoni SORIANO-ARANDES[8], Claire THORNE[9], Ricardo Arraes de Alencar XIMENES[6,10], Thália Velho Barreto de ARAÚJO[11], Vivian I. AVELINO-SILVA[12], Sarah Esperanza BETHENCOURT CASTILLO[13], Victor Hugo BORJA ABURTO[14], Patricia BRASIL[15], Celia D. C. CHRISTIE[16], Wayner Vieira DE SOUZA[17], Jose Eduardo GOTUZZO H.[18], Bruno HOEN[19,20,21], Marion KOOPMANS[22], Celina Maria Turchi MARTELLI[17], Mauro MARTINS TEIXEIRA[23], Ernesto T. A. MARQUES[17,24], Maria Consuelo MIRANDA[25], Ulisses Ramos MONTARROYOS[26], Maria Elisabeth MOREIRA[27], J Glenn MORRIS[28], Barry ROCKX[22], Paola Mariela SABA VILLARROEL[29], Carmen SORIA SEGARRA[30,31], Adriana TAMI[13,32], Marília Dalva TURCHI[33], Carlo GIAQUINTO[34] Xavier de LAMBALLERIE[35], Annelies WILDER-SMITH[36], on behalf of the EC Zika Consortia Vertical Transmission Study Group

1. Department of Population Health Sciences, Bristol Medical School, University of Bristol, Bristol, United Kingdom
2. Department of Infectious Disease Epidemiology, Faculty of Epidemiology and Population Health, London School of Hygiene & Tropical Medicine, London, United Kingdom
3. MRC Tropical Epidemiology Group, Faculty of Epidemiology and Population Health, London School of Hygiene & Tropical Medicine, London, United Kingdom
4. Fundacão Oswaldo Cruz, Flavivirus Reference Laboratory, Rio de Janeiro, Rio de Janeiro, Brazil
5. Section Clinical Tropical Medicine, Department of Infectious Diseases, Heidelberg University Hospital, Heidelberg, Germany
6. Departamento de Medicina Interna, Universidade de Pernambuco, Recife, Pernambuco, Brazil
7. Institute of Medical Biometry and Informatics, University of Heidelberg, Heidelberg, Germany
8. Pediatric Infectious Diseases and Immunodeficiencies Unit, Hospital Universitari Vall d'Hebron, Vall d'Hebron Research Institute, Barcelona, Spain
9. Great Ormond Street Institute of Child Health, University College London, United Kingdom
10. Departamento de Medicina Tropical, Universidade Federal de Pernambuco, Recife, Pernambuco, Brazil
11. Departamento de Medicina Social, Universidade Federal de Pernambuco, Recife, Pernambuco, Brazil
12. Department of Infectious and Parasitic Diseases and Instituto de Medicina Tropical, Faculdade de Medicina da Universidade de Sao Paulo, Sao Paulo, Brazil
13. Facultad de Ciencias de la Salud, Universidad de Carabobo, Valencia, Venezuela
14. Mexican Institute of Social Security, Mexico City, Mexico
15. Evandro Chagas National Institute of Infectious Diseases, Fundação Oswaldo Cruz, Rio de Janeiro, Rio de Janeiro, Brazil
16. Department of Child and Adolescent Health, University of the West Indies, Mona Campus, Kingston, Jamaica
17. Instituto Aggeu Magalhães, Fundação Oswaldo Cruz, Recife, Pernambuco, Brazil
18. Instituto de Medicina Tropical Alexander von Humboldt, Universidad Peruana Cayetano Heredia, Lima, Peru
19. INSERM Centre d'Investigation Clinique 1424, Centre Hospitalier Universitaire de Pointe-à-Pitre, Guadeloupe, France

For peer review only - http://bmjopen.bmj.com/site/about/guidelines.xhtml

20. Service de Maladies Infectieuses et Tropicales, Dermatologie, Médecine Interne, Centre Hospitalier Universitaire de Pointe-à-Pitre, Guadeloupe, France
21. Université des Antilles et de la Guyane, Faculté de Médecine Hyacinthe Bastaraud, EA 4537, Pointe-à-Pitre, Guadeloupe, France.
22. Department of Viroscience, Erasmus Medical Center, Rotterdam, The Netherlands
23. Universidade Federal de Minas Gerais, Belo Horizonte, Minas Gerais, Brazil
24. Department of Infectious Diseases and Microbiology, University of Pittsburgh, Pittsburgh, Pennsylvania, United States of America
25. Universidad Industrial de Santander, Bucaramanga, Colombia
26. Instituto de Ciências Biológicas, Universidade de Pernambuco, Recife, Pernambuco, Brazil
27. Instituto Fernandes Figueira – Fundação Oswaldo Cruz, Rio de Janeiro, Rio de Janeiro, Brazil
28. Emerging Pathogens Institute, University of Florida, Gainesville, Florida, United States of America
29. Centro Nacional de Enfermedades Tropicales, Santa Cruz de la Sierra, Bolivia
30. Universidad Católica de Santiago de Guayaquil, Ecuador
31. SOSECALI C., Ltda, Guayaquil, Ecuador
32. University of Groningen, University Medical Center Groningen, Department of Medical Microbiology and Infection Prevention, The Netherlands
33. Instituto de Patologia Tropical e Saúde Publica, Universidade Federal de Goiás, Goiânia, Goiás, Brazil.
34. Dipartimento di Salute della Donna e del Bambino, Università degli Studi di Padova, Italy
35. Université *d'*Aix-Marseille, Marseille, France
36. Department of Epidemiology and Global Health, Umeå Universite, Umeå, Sweden

Corresponding author:

Claire Thorne, Population Policy and Practice Research and Teaching Department, University College London Great Ormond Street Institute of Child Health, 30 Guilford Street, London WC1N 1EH, United Kingdom

claire.thorne@ucl.ac.uk

For peer review only

ABSTRACT

Introduction

Zika virus (ZIKV) infection in pregnancy has been associated with microcephaly and severe neurological damage to the fetus. Our aim is to document the risks of adverse pregnancy and birth outcomes and the prevalence of laboratory markers of congenital infection in deliveries to women experiencing ZIKV infection during pregnancy, using data from European Commission-funded prospective cohort studies in 20 centres in 11 countries across Latin America and the Caribbean.

Methods and analysis

We will carry out a centre-by-centre analysis of the risks of adverse pregnancy and birth outcomes, comparing women with confirmed and suspected ZIKV infection in pregnancy to those with no evidence of infection in pregnancy. We will document the proportion of deliveries in which laboratory markers of congenital infection were present. Finally, we will investigate the associations of trimester of maternal infection in pregnancy, presence or absence of maternal symptoms of acute ZIKV infection, and previous flavivirus infections with adverse outcomes and with markers of congenital infection. Centre-specific estimates will be pooled using a two-stage approach.

Ethics and dissemination

Ethical approval was obtained at each centre. Findings will be presented at international conferences and published in peer-reviewed open access journals, and discussed with local public health officials and representatives of the national Ministries of Health, Pan American Health Organization, and World Health Organization involved with ZIKV prevention and control activities.

Keywords

Zika virus; pregnancy; vertical transmission; Congenital Zika Syndrome; microcephaly; meta-analysis

For peer review only

For peer review only - http://bmjopen.bmj.com/site/about/guidelines.xhtml

Strengths and limitations of this study

- This will be a pooled analysis of data from 3 international consortia conducting prospective cohort studies of outcomes following ZIKV infection in pregnancy in 20 centres in 11 countries.
- Standardised definitions of outcomes will provide clarity about the absolute risks of adverse outcomes, which have not been reported consistently in prospective studies so far.
- These studies include a control group of women with no evidence of ZIKV infection in pregnancy, allowing improved estimation of the proportion of adverse events attributable to ZIKV in pregnancy.
- Inferences will be limited by: difficulties in distinguishing between women who did and who did not experience a ZIKV infection in pregnancy, due to the high frequency of mild and asymptomatic infections and the low sensitivity and specificity of diagnostic tools; low diagnostic sensitivity of markers of congenital infection; and outcome data that are not missing at random.

For peer review only

For peer review only - http://bmjopen.bmj.com/site/about/guidelines.xhtml

INTRODUCTION

Following the emergence of Zika virus (ZIKV) in Asia and the Pacific in 2013 (1) and the subsequent introduction to Brazil (2), clusters of neonates with severe neurological complications and microcephaly were observed across Latin America. Following recent experiences with the H1N1 influenza pandemic and Ebola outbreak in Western Africa, the need for coordinated international research on ZIKV was quickly recognized. In January 2016, before the World Health Organization (WHO) declared a Public Health Emergency of International Concern (3), the European Commission (EC) issued a funding call to set up a network in Latin America and the Caribbean with the aim of implementing and coordinating urgently required research, while simultaneously contributing to research capacity and preparedness for other emerging infectious diseases. Three consortia were funded: ZikaPLAN (4) (https://zikaplan.tghn.org/), ZIKAlliance (https://zikalliance.tghn.org/) (5,6), and ZIKAction (http://zikaction.org/). All are multidisciplinary international collaborations with active investigations in epidemiology, virology, immunology, diagnostics, mathematical modelling, social science, and animal studies. Each consortium includes its own prospective cohort study of ZIKV in pregnancy and a shared work package that aims to ensure the harmonization of protocols and data sets in order to facilitate a pooled analysis of cohort data. The primary aim of the pooled analysis is to investigate the incidence of adverse outcomes of ZIKV infection in pregnancy, including "congenital infection, microcephaly, Zika congenital syndrome, and other sequelae of ZIKV infection".

The aim of this paper is to present a protocol for this pooled analysis. Data has been or is still being collected in multiple sites in 20 regional coordinating centres spread over 11 countries and regions across Latin America and the Caribbean. There are 15 ZIKAlliance centres: Sao Paulo, Rio de Janeiro, Recife and Belo Horizonte (Brazil); Valencia (Venezuela); Bucaramanga (Colombia); Guayaquil (Ecuador); Lima (Peru); Jalisco, Nayarit, Veracruz, Yucatan (Mexico); Santa Cruz de la Sierra (Bolivia); Havana (Cuba); Guadeloupe (French Territory of the Americas); 3 ZikaPLAN centres: Goiânia, Rio de Janeiro, Recife (Brazil); and 2 ZIKAction centres: Kingston (Jamaica) and Port-au-Prince (Haiti). Recruitment to ZIKAlliance began May 2017, September 2017 for ZIKAction, and December 2015 for ZikaPLAN. Over 700 women with confirmed infection had been recruited by April 2020; recruitment continues in some centres.

Several studies of ZIKV in pregnancy have recently been published. In registry-based studies (7-9), fetuses and newborns of women with confirmed infection in pregnancy have been reported to have "potentially Zika-related" adverse outcomes at rates of up to 15%, with higher rates in the first trimester. Registry-based studies are likely to over-estimate the risk of severe clinical manifestations and underestimate the

For peer review only - http://bmjopen.bmj.com/site/about/guidelines.xhtml

risk of more mild clinical presentations because they recruit both prospectively ascertained ZIKV-infected pregnant women and women whose infection was recognized retrospectively following the birth of an infant with congenital abnormalities. Prospective studies of congenital infection have variously reported 25% “severe” and 21% “mild to moderate” outcomes in French Guiana (10), and 27% adverse outcomes in Brazil (11). The specificity of these outcome definitions for ZIKV in pregnancy is not known as these studies did not include a control group of women with no ZIKV infection in pregnancy. In another Brazilian study, the risk of adverse outcomes was reported to be 46% in births to women with NAAT (Nucleic Acid Amplification Test)-confirmed ZIKV infection in pregnancy compared to 11.5% in NAAT-negative women (12). In a large prospective study based in the French Territories of the Americas, among infants born to women with NAAT-confirmed ZIKV infection, 7.0% presented with neurologic or ocular birth defects and 3.1% met the study’s criteria for Congenital Zika Syndrome (CZS) (13), which is characterized by several unique features (14). An important limitation in the comparison of the results of these different studies is the lack of a standard definition of CZS and of the clinical and diagnostic procedures used to evaluate these children, leading to possible misclassification of the outcomes studied.

The vertical transmission rate is the probability of congenital infection in births to women with infection in pregnancy. The rates reported so far, 26% (11) and 35% (10), are based on laboratory markers of congenital infection such as NAAT or IgM in the fetus or newborn. However, a prospective cohort retrospectively reconstructed from a register study estimated the vertical transmission rate to be only 9% (15). Comparison of these rates is difficult as different markers and different biological samples were used. In addition, although these tests are analytically sensitive and specific they have poor diagnostic sensitivity as markers of congenital infection. These markers were absent from serum in a high proportion of CZS cases (16) (16,17) and in newborns with other potentially ZIKV-related adverse outcomes born to women with confirmed ZIKV during pregnancy (10,11). Clearance of virus from amniotic fluid and fetal blood has been reported in cases of CZS, even when ZIKV is found in brain tissue post-mortem (18,19). It therefore appears that fetal infection may occur, causing profound damage, but clearing before delivery and leaving no discernable immunological trace in serum. Consequently, in this study we will document the prevalence of markers of congenital infection using uniform criteria, recognizing that this is an underestimate of the true vertical transmission rate.

Regarding effect modifiers, a number of studies have reported a higher incidence of congenital abnormalities following maternal infections in the first trimester (7,10–11,14). Maternal symptoms during acute ZIKV infection do not appear to be a risk factor for adverse outcomes (20). There is evidence of

For peer review only - http://bmjopen.bmj.com/site/about/guidelines.xhtml

antibody dependent enhancement of ZIKV by dengue virus (DENV) antibody in animal models (21), but it is unclear whether previous DENV infection or exposure to other flaviviruses has a protective, risk-enhancing, or null effect, in maternal or congenital infection in humans (22). It also remains to be established whether a previous ZIKV infection confers protective immunity. Little is currently known about risk factors for trans-placental transmission of ZIKV.

The analysis plan described here complements the recently published protocol of the Zika Virus Individual Participant Data (IPD) Consortium (23), which will eventually include data from the three EC consortia as well as data from many other sources. Although the objectives of the protocols are similar, different methods are proposed in relation to design of included studies, definition of congenital infection, and approach to imperfect diagnosis of maternal infection.

In light of the unexplained heterogeneity in reported rates of adverse outcomes, and the variation in prevalence of markers of congenital infection, a pooled analysis of data from 20 centers following similar protocols with harmonized definitions of clinical and laboratory outcomes will provide important new information on outcomes of ZIKV in pregnancy.

OBJECTIVES OF THE JOINT ANALYSIS

1. To estimate the risk of adverse outcomes in the fetus, newborn, and child following maternal ZIKV infection in pregnancy, compared to outcomes in controls with no evidence of maternal infection in pregnancy.
2. To estimate the prevalence of markers of congenital infection in deliveries to women with ZIKV infection during pregnancy.
3. To assess the associations between trimester of maternal infection, presence or absence of maternal symptoms, and previous flavivirus infections with adverse outcomes and markers of congenital infection.

METHODS

Participants

For peer review only - http://bmjopen.bmj.com/site/about/guidelines.xhtml

Pregnant women were eligible only if their infection status during pregnancy (infected or not infected) was ascertained prior to the detection of adverse outcomes, or was not influenced by fetal examination or outcome on delivery. This definition is compatible with retrospective testing of previously collected maternal samples, after delivery. Although the unit of recruitment is the mother, the unit of analysis is the fetus, newborn, and infant; multiple births are sufficiently rare to be treated as independent observations (24).

Study design

Consenting women were screened in pregnancy for markers of ZIKV infection. Those in whom maternal infection in pregnancy (MIP) was suspected were followed with enhanced investigations. In ZIKAction and ZIKAlliance, all deliveries to these women, including fetal losses, stillbirths, and newborns were examined clinically and tested for markers of congenital infection. This testing was not routinely performed in ZikaPLAN. In all three cohorts, newborns were prospectively followed to identify any adverse outcomes that may develop later. In all three consortia, a sample of newborns delivered to women with no evidence of infection in pregnancy served as an unexposed control group.

There were some differences between the protocols adopted by the three consortia in terms of how women were recruited into the study, and the choice and scheduling of tests and investigations (Supplementary Table S1). In ZIKAction and ZIKAlliance, women were recruited regardless of symptoms during pregnancy, although report of symptoms was recorded. In ZikaPLAN, only women with rash, a common sign of ZIKV infection, were recruited. Statistical analyses will therefore be stratified by whether the mother reported symptoms in pregnancy. .

When the studies were designed, there was little information on the risk of adverse outcomes of ZIKV in pregnancy, on vertical transmission rates, nor on what infection rates among pregnant women might be expected. Formal sample size calculations were not undertaken.

Target parameters and terminology of vertical transmission studies

Six categories of joint congenital infection status and maternal infection status (A-F) are defined in Table 1, which illustrates the logic of an idealised prospective study. The usual target parameters are the *vertical transmission rate*, which is the probability of congenital infection following MIP, (A+B) / (A+B+C+D); and the *rate of adverse outcomes in those with congenital infection,* A/(A+B). The definitions of "adverse

For peer review only - http://bmjopen.bmj.com/site/about/guidelines.xhtml

outcomes", congenital infection, and MIP will be determined by a Joint Diagnostics Group and a Joint Endpoint Review Group, after the data have been assembled. Estimates of these parameters are standard in the classic studies of vertical transmission of human immune-deficiency virus (HIV) (25,26), toxoplasmosis (27–29) and cytomegalovirus (CMV) (30). In studies of less specific outcomes, the event rate C/(C+D) in fetuses and newborns of women with MIP but in whom no congenital infection occurred (Paediatric Control Group 1 in Table 1) forms a comparison group (31,32) representing the adverse event rate that is due to MIP in the absence of congenital infection. The present analysis plan is modelled closely on these earlier studies, but includes adaptations to take account of the difficulties in diagnosing maternal and congenital ZIKV infection.

For example, because cases of congenital infection cannot be reliably identified by diagnostic tests, we can only estimate the prevalence of laboratory *markers of vertical infection* (Objective 2). Similarly, the "overall" (unconditional) adverse event rate is taken as the primary outcome for Objective 1; this includes all births to women with MIP, (A+C)/(A+B+C+D) (Table 1). All three consortia included a further control group of births to unexposed women, those with no infection in pregnancy (Maternal Control Group in Table 1). The adverse event rates in this group, E/(E+F), represents a baseline for comparison with the overall event rates in exposed women (33).

Although estimates of the vertical transmission rate are compromised, it may still be of interest to compare adverse outcome rates in the MIP with congenital infection group (i.e., A/(A+B)), the MIP without congenital infection group (i.e., C/(C+D)), and the No MIP group (i.e., E/(E+F)), as this may provide insight into whether adverse fetal outcomes are associated with MIP in the absence of demonstrable fetal infection.

In addition to the challenges associated with the laboratory definition of congenital infection, it is also difficult to discriminate between pregnancies with MIP and with No MIP as required in analyses based on Table 1. A positive NAAT result or seroconversion during pregnancy are sufficient to confirm MIP. However, even if tested *per protocol,* MIP may be missed due to the narrow window of detection of NAAT tests, perhaps as low as 14 days (34). Tests of recent infection, including IgM, IgG3 or avidity assays, may reflect infection during pregnancy, but may also be the result of infection prior to pregnancy (35), and immunologic cross-reactivity to DENV antibody may need to be ruled out (36). These tests therefore indicate suspected but not confirmed MIP. An IgG negative response in the woman or newborn at or tests, perhaps as low as 14 days (34). Tests of recent infection, including IgM, IgG3 or avidity assays, may reflect infection during pregnancy, but may also be the result of infection prior to pregnancy (35), and

For peer review only - http://bmjopen.bmj.com/site/about/guidelines.xhtml

immunologic cross-reactivity to DENV antibody may need to be ruled out (36). These tests therefore indicate suspected but not confirmed MIP. An IgG negative response in the woman or newborn at or shortly after delivery can be taken as suggestive of No MIP, although the dynamics of ZIKV IgG are not well documented. While a negative IgG is likely to be uncommon in some sites, as some women will have experienced a ZIKV infection prior to pregnancy, it lowers the probability that a ZIKV infection would have occurred in pregnancy. Taking all this together, MIP status will be characterised as “Confirmed”, “Suspected, “No Evidence of MIP" (i.e., all NAAT and IgM tests negative), and "No MIP" (i.e., all NAAT and IgM tests negative AND IgG negative at or shortly after delivery). To maintain the principle of prospective ascertainment, confirmation of congenital infection will have no impact on the mother’s assigned infection status.

Definitions of variables

To support the pooled analyses, a Joint Diagnostics Group consisting of immunologists and virologists and a Joint Endpoint Review Group consisting of paediatricians with experience of congenital ZIKV will be convened to agree on standardized case definitions. The names and qualifications of members of both groups will be published at the time of the joint data analysis, together with rationale and process for their recruitment. Supplementary Table S2 provides some provisional definitions for: MIP (Confirmed, Suspected, No Evidence of MIP, No MIP), laboratory markers of congenital infection (present or absent), signs and symptoms compatible with Congenital Zika Syndrome (CZS), other potentially-Zika-related outcomes (OPZRO), and trimester of MIP. The Diagnostics Group will also have responsibility for definitions of covariates, such as previous flavivirus infection. Definitions of both diagnostic categories and clinical endpoints will be based on the best information available at the time of analysis, and will to the greatest extent possible, be harmonized across consortia and across centres within consortia. As the expert groups will be assembled from representatives of each consortium, they will already have examined the data prior to a joint analysis, so that blinding will not be possible; however, they will devise diagnostic and clinical criteria that can be applied objectively across the three consortia.

An essential aspect of the definitions for MIP status used in the statistical analyses below is that they must be based exclusively on the *prospective* diagnostic testing. For example, although CZS and/or laboratory evidence of congenital infection in the newborn provides compelling evidence of MIP, this would not affect the MIP status as ascertained prospectively. Thus, we expect to observe some newborns with CZS and/or with laboratory markers of congenital infection delivered to women with Suspected MIP, or even those categorized as having No Evidence of MIP.

For peer review only - http://bmjopen.bmj.com/site/about/guidelines.xhtml

STATISTICAL ANALYSIS

Analysis of data from individual centres

Descriptive tables

We will produce descriptive tables providing a breakdown of the numbers in each centre with Confirmed MIP, Suspected MIP, No Evidence of MIP, and No MIP, and the type of evidence on which this is based (e.g., NAAT, seroconversion, tests of recent infection) (Supplementary Table S3). The similar Supplementary Table S4 will document numbers with signs or symptoms compatible with CZS and with markers of congenital infection, and the type of evidence on which this is based, for example NAAT, IgM, or clinical markers (CZS).

Objective 1: Adverse Outcomes

Prospectively ascertained MIP status will be tabulated against overall (i.e., unconditional on congenital infection status) adverse outcomes (Table 2). Various risks can be estimated within each MIP category, including: the risk of signs and symptoms compatible with CZS, the risk of OPZRO, the risk of both combined, of individual signs and symptoms, or of signs and symptoms grouped in clinically (e.g., ophthalmologic defects) or embryologically meaningful ways. Outcomes may be binary (e.g., microcephaly) or continuous (e.g., head circumference), or multi-category (CZS-related outcomes, OPZRO, asymptomatic). As well as congenital anomalies, rates of standard outcomes, in the absence of congenital anomalies, will be documented, including: fetal loss, stillbirth, low birthweight, intra-uterine growth retardation (IUGR), and premature delivery.

The rate of adverse outcomes in the No MIP group represents the study-specific background rate of adverse outcomes (i.e., in the absence of MIP, Control Group 2), to be compared to rates in women with Confirmed MIP. Absolute risks will be estimated as well as risk ratios and risk differences.

Parallel sets of estimates will be calculated in the Suspected MIP and No Evidence of MIP groups, as particular adverse events indicate lack of diagnostic specificity and sensitivity in the testing protocol

For peer review only - http://bmjopen.bmj.com/site/about/guidelines.xhtml

during pregnancy and are therefore informative regarding the effectiveness of the maternal testing protocol in each centre; for example, microcephaly in the No Evidence of MIP group would indicate a lack of sensitivity. These sets of estimates may be pooled, respectively, with adverse outcome risks in Confirmed MIP and No MIP groups in sensitivity analyses.

Objective 2: Laboratory and clinical markers of vertical transmission

Estimates of the prevalence of markers of congenital infection will be produced in all centres with available data for each MIP group except the No MIP group, as the definition of No MIP is not compatible with laboratory markers of congenital infection. For this purpose, the numerator will be the number with markers of congenital infection, and the denominator will be the sum of the numbers with and without markers of congenital infection. Separate estimates will be obtained for: laboratory markers, clinical markers (namely CZS) , and combined laboratory and clinical markers (Table 3). Rates in the No Evidence of MIP group are of interest as they carry information about the diagnostic accuracy of the maternal testing protocol.

Objective 3: Effect of covariates

Separate estimates of the prevalence of adverse pregnancy outcomes, and the probability of laboratory and clinical markers of congenital infection, will be produced for each trimester of maternal infection, and by presence or absence of maternal symptoms.

Analyses of adverse event frequencies and of markers of congenital infection can be extended to include multiple covariates, using logistic regression. These might include potential *effect modifiers,* such as previous arbovirus infection or co-infection, or *confounding factors* such as socio-economic indicators likely to be associated with both arbovirus exposure and adverse outcomes. However, at the time of writing it is not known whether sufficient data will be available for regression analyses.

Secondary objectives

ZIKV infection in pregnancy could lead to adverse pregnancy and birth outcomes either following a congenital infection (cell A in Table 1) or in the absence of congenital infection (equally in cells A and C).

For peer review only - http://bmjopen.bmj.com/site/about/guidelines.xhtml

Although absence of markers of congenital infection does not rule out congenital infection, we might still expect to observe more adverse outcomes in fetuses and newborns with laboratory markers of congenital infection if those outcomes are caused by congenital infection. By contrast, adverse outcomes that are the result of MIP in the absence of congenital infection (Cell E in Table 1) should occur equally with or without laboratory markers of congenital infection. Based on literature on other infections in pregnancy, including dengue virus (37), adverse outcomes associated with MIP in the absence of congenital infection potentially include: fetal loss, stillbirth, prematurity, IUGR, and low birthweight for gestational age (38). The analysis would be based on a tabulation of presence or absence of neonatal symptoms, or sets of symptoms (Supplementary Table S5), and would be stratified by trimester of maternal infection, as this is likely to be associated with the presence of markers of congenital infection and with adverse outcomes.

Missing Covariates

All analyses will be conducted on a "complete case" basis, in the first instance. Methods for handling missing covariates, such as imputation (39), will be considered after the extent and patterns of missing data have been explored.

Combining data across centres

The above analyses will generate a series of centre-specific estimates of proportions, relative risks comparing MIP and No MIP groups, risk-differences, and means of continuous variables, stratified by trimester and maternal clinical presentation. If logistic regression is used to examine effect-modifiers and confounders, further estimates of interaction terms or adjusted estimates can also be produced.

All these estimates can be combined across centres using fixed or random effects models in a "two-stage" meta-analysis. Bayesian Markov chain Monte Carlo methods will be used as this will facilitate the use of exact binomial and multinomial likelihoods, which have a better performance with low and zero cells counts. Vague priors will be employed. Centre-specific random effect estimates will be sampled from beta distributions for binomial outcomes data, Dirichlet distributions for multinomial data, and normal distributions for continuous data. We will report ranges, between-centre standard deviations, mean effects and predictive effects with 95% credible intervals for each estimate.

For peer review only - http://bmjopen.bmj.com/site/about/guidelines.xhtml

In combining estimates from different centres, we will take account of the fact that in ZikaPLAN only women with rash were recruited, so that women with no ZIKV infection may have experienced other exanthematic infections (12), including arbovirus infections such as dengue and chikungunya, which may themselves be associated with adverse outcomes (40,41).

Depending on the results of two-stage analyses, and the completeness of covariate data, an individual patient data one-stage meta-analyses will be considered for each objective, as a secondary or sensitivity analysis, with centre as an additional fixed "intercept" term.

Sensitivity analyses

Definitions of MIP status, ZIKV-related outcomes, and laboratory markers of congenital infection

We will report differences in adverse event rates between Confirmed MIP and Suspected MIP, and between No Evidence of MIP and No MIP. If the differences are small, we will produce results pooling these categories as a sensitivity analysis.

Further, the No Evidence of MIP category can be subdivided into women who were tested *per protocol* and those who may have been tested less completely. The impact of compliance with protocols will be explored, as it is expected to impact on the proportion of women with MIP who are classified as "No evidence of MIP" and, hence, on the probability of observing adverse outcomes in this group. Similarly, we will conduct sensitivity analyses around the definitions of Confirmed and Suspected MIP on advice from the Joint Diagnostics Group.

Alternative sets of estimates will be generated using alternative criteria for CZS-related outcomes and OPZRO, that are more, or less, specific for ZIKV in pregnancy. Similarly, we will explore the impact of varying the laboratory criteria for congenital infection on the advice of the Joint Diagnostics Group.

Independent ascertainment of outcomes

A critical requirement of all these analyses is that the ascertainment of markers of congenital infection status and clinical outcomes in the fetuses and newborns, and developing infant are all independent of

For peer review only - http://bmjopen.bmj.com/site/about/guidelines.xhtml

each other, and also independent of MIP status. For example, ideally the same laboratory testing for congenital infection is carried out regardless of whether the pregnancy outcome is a fetal loss, termination of pregnancy, stillbirth, a case of CZS, or an apparently healthy asymptomatic infant.

These assumptions are difficult, and in certain respects (e.g., first trimester fetal loss and terminations) not possible, to fully implement in practice. To address these inevitable limitations in the analysis, which are expected to impact more on objective 2 than on objective 1, we will carry out exploratory analyses aimed at detecting potential deviations from protocol. For example, the distribution of trimester of MIP should not be associated with prospectively ascertained MIP status. Guided by the results, we will carry out sensitivity analyses that make a range of assumptions about the distribution of missing data, especially data on markers of congenital infection. A series of scenarios will be examined to assess robustness of results to inherent and/or unplanned deviations from the ideal protocols required for unbiased estimation of the target parameters.

DISCUSSION

Risks of CZS and other adverse birth outcomes of ZIKV infection in pregnancy can only be assessed through studies that recruit women whose infection status is prospectively ascertained, or, if retrospective, ascertainment is independent of outcomes. However, reported risks of adverse outcomes even from prospective studies have been highly variable (10–13), as have vertical transmission rates based on laboratory markers of congenital ZIKV infection (10,11,15). An important role for joint analyses of multiple studies is to explore whether this heterogeneity in outcomes can be explained by individual or study-level covariates. To do this, it is essential that incidental sources of variation, such as those arising from differences in outcome reporting or diagnostic testing, are controlled or eliminated as much as possible. One of the most difficult sources of variation between consortia, and between sites within consortia, lies in diagnosis of maternal infection. Our approach is to have an Expert Diagnostics Group produce a harmonised classification of Confirmed MIP, Suspected MIP, No Evidence of MIP, and No MIP, and to compute a range of estimates of the relative effect of maternal infection on outcomes, grouping these in different ways. An analysis based on the Confirmed MIP and No MIP groups alone would be expected to generate the largest estimates of relative effect, because both poor sensitivity and poor specificity will tend to bias effect estimates towards the null.

An alternative proposal (23) in relation to maternal infection status is to treat test sensitivity and specificity as study-level covariates in a meta-regression, but the risk of false positive diagnosis depends

For peer review only - http://bmjopen.bmj.com/site/about/guidelines.xhtml

more on the incidence of ZIKV and cross-reacting antibodies to other arbovirus infections such as dengue than on test specificity. The Zika Virus IPD Consortium protocol differs in two other ways. First it proposes to include surveillance studies, which may result in over-estimating the risk of adverse outcomes due to retrospective ascertainment of infected women following adverse newborn outcomes (7,42). Second, congenital infection is to be defined by clinical and radiological criteria alone.

Other statistical methods may have been developed by the time the data becomes available for these analyses. Whatever form of analysis is adopted, a standardised pooled analysis from three large consortia comprising 20 centres will provide valuable information about these parameters, which will assist in framing a public health response and advice to women who might be exposed in future. It may also throw light on pathologic mechanisms leading to adverse outcomes, which could help in the development of therapeutic or prophylactic interventions.

Patient and Public Involvement

No patients were involved in the development of this protocol

Acknowledgements The authors express their thanks to Tom Byrne for organising the references.

Collaborators Other members of the EC Zika Consortia Vertical Transmission Study Group are: Joshua ANZINGER (Department of Microbiology, Virology Section, University of the West Indies, Mona Campus, Kingston, Jamaica; Global Virus Network, Baltimore, MD, USA), Heather BAILEY (Institute for Global Health, University College London, UK) Valery E M BEAU DE ROCHARS (Emerging Pathogens Institute, University of Florida, Gainesville, Florida, United States of America), Luana DAMASCENO (Evandro Chagas National Institute of Infectious Diseases, Fundação Oswaldo Cruz, Rio de Janeiro, Brazil), Maria de Fatima Pessoa Militão DE ALBUQUERQUE (Instituto Aggeu Magalhães, Fundação Oswaldo Cruz, Recife, Pernambuco, Brazil), Rafael F. O. FRANÇA (Instituto Aggeu Magalhães, Fundação Oswaldo Cruz, Recife, Pernambuco, Brazil), Hugo Lopez GATELL RAMIREZ (Centro de Investigación sobre Enfermedades Infecciosas, Instituto Nacional de Salud Pública, Cuernavaca, Mexico), Adriana GOMEZ (Universidad Industrial de Santander, Bucaramanga, Colombia), Fabiana GORDON (Population Health Sciences, Bristol Medical School, University of Bristol, Bristol, United Kingdom), Maria G GUZMAN (Instituto de Medicina Tropical Pedro Kourí, La Havana, Cuba), Daniel LANG (Fondazione Penta Onlus, São Paulo, São Paulo, Brazil), Rigan LOUIS (Emerging Pathogens Institute, University of Florida, Gainesville, Florida, United States of America), Anyela Lozano (Universidad Industrial de Santander, Bucaramanga, Colombia), Eric

For peer review only - http://bmjopen.bmj.com/site/about/guidelines.xhtml

MARTINEZ (Instituto de Medicina Tropical Pedro Kourí, La Havana, Cuba), Philippe MAYAUD (Department of Clinical Research, London School of Hygiene and Tropical Medicine, London, UK), Ricard ORTIZ SERRANO (Universidad Autónoma de Bucaramanga, Bucaramanga, Colombia), Silvia P. SALGADO (Instituto Nacional de Salud Pública e Investigación, Guyaquil, Ecuador), Aluisio Augusto Cotrim SEGURADO (Department of Infectious and Parasitic Diseases and Instituto de Medicina Tropical, Faculdade de Medicina da Universidade de Sao Paulo, Sao Paulo, Brazil), Zilton F. M. VASCONCELOS (Instituto Fernandes Figueira – Fundação Oswaldo Cruz, Rio de Janeiro, Rio de Janeiro, Brazil), Isabelle Freire Tabosa VIANA (Instituto Aggeu Magalhães, Fundação Oswaldo Cruz, Recife, Pernambuco, Brazil), Luis Angel VILLAR (Universidad Industrial de Santander, Bucaramanga, Colombia)

Contributors A steering group (CT, TJ, KDR, RAAX, AEA, EBB, NA) was formed to prepare a Joint Statistical Analysis Plan pursuant to the terms of the three EC Zika Consortia funding, at the direction of the consortia PIs (CG, XdL and AW-S). After initial discussions the steering group delegated the task to AEA, EBB and NA. The SAP was conceptualised and drafted by AEA with EBB and NA. After several iterations, the draft was revised based on comments from CT, DB, ASA, TJ, KDR, MP, DBM, RAAX, MCM, AT, CG, XdL and AW-S. Comments were then solicited from TVBA, VIAS, SEBC, VHBA, PB, CDCC, WVS, JEGH, BH, MK, CMTM, MMT, ETAM, URM, MEM, JGM, BR, PMSV, CSS and MDT and a revision was drafted by AEA, EBB and NA. The final draft was agreed by all authors.

Funding European Union Directorate-General for Research grant numbers 734857 (ZIKAction), 734548 (ZIKAlliance), 734584 (ZikaPLAN), Horizon 2020 programme of the European Commission. EBB was also supported by Wellcome Trust & the UK's Department for International Development (205377/Z/16/Z to LCR; https://wellcome.ac.uk/).

Patient consent for publication Not required

Competing interests None

For peer review only - http://bmjopen.bmj.com/site/about/guidelines.xhtml

TABLES

Table 1. Design of an idealized prospective vertical transmission study. The vertical transmission rate is estimated by (A+B)/(A+B+C+D). The rate of adverse outcomes conditional on congenital infection is A/(A+B). This can be compared with the rate of adverse outcomes in newborns with no congenital infection, C/(C+D), who form a control group (Pediatric Control Group) to account for potential confounders associated with maternal infection.

An overall, non-conditional estimate of the adverse event rate is (A+C)/(A+B+C+D).

Follow-up of births to women with no MIP creates a second control group (Maternal Control Group), in which the rate of adverse outcomes, E/(E+F), can be compared to the rate in births with no congenital infection to women with MIP. Estimated effects of MIP based on the Maternal Control Group are vulnerable to confounding by factors associated with MIP. Some cells are set to zero as there can be no congenital infection without MIP.

<table>
<tr><td colspan="3" rowspan="2"></td><td colspan="2">Maternal Infection Status</td></tr>
<tr><td>MIP</td><td>No MIP</td></tr>
<tr><td rowspan="4">Congenital Infection Status</td><td rowspan="2">congenital infection</td><td>Adverse outcomes</td><td>A</td><td>0</td></tr>
<tr><td>No Adverse outcomes</td><td>B</td><td>0</td></tr>
<tr><td rowspan="2">No congenital infection</td><td>Adverse outcomes</td><td>C</td><td>E</td></tr>
<tr><td>No Adverse outcomes</td><td>D</td><td>F</td></tr>
</table>

For peer review only

For peer review only - http://bmjopen.bmj.com/site/about/guidelines.xhtml

Table 2. Scheme for a generic analysis of risks of adverse outcomes by prospectively ascertained Maternal Infection in Pregnancy (MIP) status. Presence or absence of fetal and neonatal signs or symptoms (e.g., microcephaly, brain calcifications, arthrogryposis) and other Potentially Zika-related outcomes (e.g., fetal loss) in the different MIP groups will be compared.

**CENTRE**	**Maternal Infection in Pregnancy status**			
	Confirmed	Suspected	No Evidence of MIP	No MIP
**Symptom 1**				
**Symptom 2**				
**Symptom 3**				
**:**				
**No Symptoms**				
**TOTAL**				

Table 3. Scheme for generic analysis of markers of congenital infection by prospectively ascertained Maternal Infection in Pregnancy (MIP) status. Markers of congenital infection may include laboratory markers, clinical markers, such as microcephaly, or both combined.

**CENTRE**	**Maternal Infection in Pregnancy status**			
	Confirmed	Suspected	No Evidence of MIP	No MIP
**Both laboratory and clinical markers of congenital infection**				
**Only laboratory markers of congenital infection**				
**Only clinical markers of congenital infection**				

For peer review only - http://bmjopen.bmj.com/site/about/guidelines.xhtml

**No markers of congenital infection**				
**Not tested**				
**TOTAL**				

For peer review only

For peer review only - http://bmjopen.bmj.com/site/about/guidelines.xhtml

References

1. Roth A, Mercier A, Lepers C, Hoy D, Duituturaga S, Benyon E, et al. Concurrent outbreaks of dengue, chikungunya and zika virus infections null An unprecedented epidemic wave of mosquito-borne viruses in the Pacific 2012null2014. Eurosurveillance. 2014;19(41):1–8.
2. Campos GS, Bandeira AC, Sardi SI. Zika Virus Outbreak, Bahia, Brazil. Emerg Infect Dis. 2015;21(10):1885–6.
3. Gulland A. Zika virus is a global public health emergency, declares WHO. BMJ. 2016;352(February):i657.
4. Wilder-Smith A, Preet R, Renhorn KE, Ximenes RA, Rodrigues LC, Solomon T, et al. ZikaPLAN: Zika Preparedness Latin American Network. Glob Health Action. 2017;10(1):1398485.
5. Avelino-Silva VI, Mayaud P, Tami A, Miranda MC, Rosenberger KD, Alexander N, et al. Study protocol for the multicentre cohorts of Zika virus infection in pregnant women, infants, and acute clinical cases in Latin America and the Caribbean: The ZIKAlliance consortium. BMC Infect Dis. 2019;19(1):1–14.
6. De Alencar Ximenes RA, De Barros Miranda-Filho D, Brickley EB, Montarroyos UR, Martelli CMT, de Araújo TVB, et al. Zika virus infection in pregnancy: Establishing a case definition for clinical research on pregnant women with rash in an active transmission setting. PLoS Negl Trop Dis. 2019;13(10):1–17.
7. Reynolds M, Jones A, Petersen E, Lee E, Rice M, Bingham A. Vital Signs: Update on Zika Virus–Associated Birth Defects and Evaluation of All U.S. Infants with Congenital Zika Virus Exposure — U.S. Zika Pregnancy Registry, 2016. Morb Mortal Wkly Rep. 2017;66(13):366–73.
8. Shapiro-Mendoza CK, Rice ME, Galang RR, Fulton AC, VanMaldeghem K, Prado MV, et al. Pregnancy Outcomes After Maternal Zika Virus Infection During Pregnancy — U.S. Territories, January 1, 2016–April 25, 2017. MMWR Morb Mortal Wkly Rep. 2017;66(23):615–21.
9. Honein MA, Dawson AL, Petersen EE, Jones AM, Lee EH, Yazdy MM, et al. Birth defects among fetuses and infants of US women with evidence of possible zika virus infection during pregnancy. JAMA - J Am Med Assoc. 2017;317(1):59–68.
10. Pomar L, Vouga M, Lambert V, Pomar C, Hcini N, Jolivet A, et al. Maternal-fetal transmission and adverse perinatal outcomes in pregnant women infected with Zika virus: prospective cohort study in French Guiana. Bmj. 2018;k4431.
11. Nogueira ML, Nery Júnior NRR, Estofolete CF, Bernardes Terzian AC, Guimarães GF, Zini N, et al. Adverse birth outcomes associated with Zika virus exposure during pregnancy in São José do Rio Preto, Brazil. Clin Microbiol Infect. 2018 Jun;24(6):646–52.
12. Brasil P, Pereira J, Moreira E, Riberio Nogueira RM, Damasceno L, Wakimoto M, et al. Zika Virus Infection in Pregnant Women in Rio de Janeiro. N Engl J Med. 2016;56(11):1024–5.
13. Hoen B, Schaub B, Funk AL, Ardillon V, Boullard M, Cabié A, et al. Pregnancy Outcomes after ZIKV Infection in French Territories in the Americas. N Engl J Med. 2018;378(11):985–94.
14. Moore CA, Staples JE, Dobyns WB, Pessoa A, Ventura C V., Da Fonseca EB, et al. Characterizing the pattern of anomalies in congenital zika syndrome for pediatric clinicians. JAMA Pediatr. 2017;171(3):288–95.
15. Conners EE, Lee EH, Thompson CN, Mcgibbon E, Rakeman JL, Iwamoto M, et al. Zika Virus Infection Among PregnantWomen and Their Neonates in NewYork City, January 2016–June 2017. Obs Gynecol. 2018;132:487–95.
16. van der Linden V, Pessoa A, Dobyns W, Barkovich AJ, Júnior H van der L, Filho ELR, et al. Description of 13 Infants Born During October 2015–January 2016 With Congenital Zika

For peer review only - http://bmjopen.bmj.com/site/about/guidelines.xhtml

Virus Infection Without Microcephaly at Birth — Brazil. Morb Mortal Wkly Rep. 2016;65(47):1343–8.
17. de Araújo TVB, Ximenes RA de A, Miranda-Filho D de B, Souza WV, Montarroyos UR, de Melo APL, et al. Association between microcephaly, Zika virus infection, and other risk factors in Brazil: final report of a case-control study. Lancet Infect Dis. 2018 Mar;18(3):328–36.
18. Schaub B, Vouga M, Najioullah F, Gueneret M, Monthieux A, Harte C, et al. Analysis of blood from Zika virus-infected fetuses: a prospective case series. Lancet Infect Dis. 2017;17(5):520–7.
19. Rodó C, Suy A, Sulleiro E, Soriano-Arandes A, Antón A, García-Ruiz I, et al. In utero negativization of Zika virus in a foetus with serious central nervous system abnormalities. Clin Microbiol Infect. 2018;24(5):549.e1-549.e3.
20. Paixao ES, Leong WY, Rodrigues LC, Wilder-Smith A. Asymptomatic prenatal Zika virus infection and congenital Zika syndrome. Open Forum Infect Dis. 2018;5(4):1–5.
21. Bardina S V., Bunduc P, Tripathi S, Duehr J, Frere JJ, Brown JA, et al. Enhancement of Zika virus pathogenesis by preexisting antiflavivirus immunity. Science (80-). 2017;356(6334):175–80.
22. Masel J, McCracken MK, Gleeson T, Morrison B, Rutherford G, Imrie A, et al. Does prior dengue virus exposure worsen clinical outcomes of Zika virus infection? A systematic review, pooled analysis and lessons learned. PLoS Negl Trop Dis. 2019;13(1):1–14.
23. Wilder-Smith A, Wei Y, Araújo TVB de, VanKerkhove M, Turchi Martelli CM, Turchi MD, et al. Understanding the relation between Zika virus infection during pregnancy and adverse fetal, infant and child outcomes: a protocol for a systematic review and individual participant data meta-analysis of longitudinal studies of pregnant women and their inf. BMJ Open. 2019;9(6):e026092.
24. Thorne C, Malyuta R, Semenenko I, Pilipenko T, Patel D, Bunders M, et al. The mother-to-child HIV transmission epidemic in Europe: Evolving in the East and established in the West. Aids. 2006;20(10):1419–27.
25. Mandelbrot, Mayaux, Bongain, Berrebi, Moudoub-Jeanpetit, Bénifla, et al. Obstetric factors and mother-to-child transmission of human immunodeficiency virus type 1: The French perinatal cohorts. Am J Obstet Gynecol. 1996;175(3):661–7.
26. Dunn DT, Ades AE. Estimating the HIV Vertical Transmission Rate and the Pediatric AIDS Incubation Period from Prospective Data AU. J Am Stat Assoc. 1996 Sep 1;91(435):935–43.
27. Gras L, Gilbert RE, Ades AE, Dunn DT. Effect of prenatal treatment on the risk of intracranial and ocular lesions in children with congenital toxoplasmosis. Int J Epidemiol. 2001;30(6):1309–13.
28. Gilbert RE, Gras L, Wallon M, Peyron F, Ades AE, Dunn DT. Effect of prenatal treatment on mother to child transmission of Toxoplasma gondii: Retrospective cohort study of 554 mother-child pairs in Lyon, France. Int J Epidemiol. 2001;30(6):1303–8.
29. The SYROCOT (Systematic Review on Congenital Toxoplasmosis) study group. Eff ectiveness of prenatal treatment for congenital toxoplasmosis: a meta-analysis of individual patients' data. Lancet. 2007;369(9556):115–22.
30. Stagno S, Pass RF, Dworsky ME, Henderson RE, Moore EG, Walton PD, et al. Congenital Cytomegalovirus Infection: The relative importance of primary and recurrent maternal infection. N Engl J Med. 1982 Apr;306(16):945–9.
31. Townsend CL, Forsgren M, Ahlfors K, Ivarsson SA, Tookey PA, Peckham CS. Long-term outcomes of congenital cytomegalovirus infection in Sweden and the United Kingdom. Clin Infect Dis. 2013;56(9):1232–9.

For peer review only - http://bmjopen.bmj.com/site/about/guidelines.xhtml

32. Preece PM, Blount JM, Glover J, Fletcher GM, Peckham CS, Griffiths PD. The consequences of primary cytomegalovirus infection in pregnancy. Arch Dis Child. 1983;58(12):970–5.
33. Li XL, Wei HX, Zhang H, Peng HJ, Lindsay DS. A meta analysis on risks of adverse pregnancy outcomes in Toxoplasma gondii infection. PLoS One. 2014;9(5):1–12.
34. Paz-Bailey G, Rosenberg ES, Doyle K, Munoz-Jordan J, Santiago GA, Klein L, et al. Persistence of Zika Virus in Body Fluids — Final Report. N Engl J Med. 2018;NEJMoa1613108.
35. Centers for Disease Control and Prevention. Prolonged IgM Antibody Response in People Infected with Zika Virus : Implications for Interpreting Serologic Testing Results for Pregnant. 2017;5–7.
36. Rabe IB, Staples JE, Villanueva J, Hummel KB, Johnson JA, Rose L, et al. Interim Guidance for Interpretation of Zika Virus Antibody Test Results. MMWR Morb Mortal Wkly Rep. 2016;65(21):543–6.
37. Paixão ES, Teixeira MG, Costa M da CN, Barreto ML, Rodrigues LC. Symptomatic Dengue during Pregnancy and Congenital Neurologic Malformations. Emerg Infect Dis. 2018 Sep;24(9):1748–50.
38. Cardenas I, Means RE, Aldo P, Koga K, Lang SM, Booth C, et al. Viral Infection of the Placenta Leads to Fetal Inflammation and Sensitization to Bacterial Products Predisposing to Preterm Labor. J Immunol. 2010;185(2):1248–57.
39. JL. S. Multiple imputation: a primer. Stat Methods Med Res. 1999;8(1):3–15.
40. Basurko C, Matheus S, Hildéral H, Everhard S, Restrepo M, Cuadro-Alvarez E, et al. Estimating the risk of vertical transmission of dengue: A prospective study. Am J Trop Med Hyg. 2018;98(6):1826–32.
41. Contopoulos-Ioannidis D, Newman-Lindsay S, Chow C, LaBeaud AD. Mother-to-child transmission of Chikungunya virus: A systematic review and meta-analysis. PLoS Negl Trop Dis. 2018;12(6):1–20.
42. Honein MA, Dawson AL, Petersen EE, Jones AM, Lee EH, Yazdy MM, et al. Birth defects among fetuses and infants of US women with evidence of possible zika virus infection during pregnancy. JAMA - J Am Med Assoc. 2017 Jan 3;317(1):59–68.

For peer review only

For peer review only - http://bmjopen.bmj.com/site/about/guidelines.xhtml

SUPPLEMENTARY MATERIALS

Supplementary Table S1. Schedule of tests and examinations in pregnancy, at delivery, and during paediatric follow-up, in each consortium.

Consortium	Mother	Fetus	Newborn and neonate	Pediatric follow-up
ZIKAlliance	ZIKV serology IgG & IgM, NAAT blood and urine, at booking, every 4w, at birth, and at paediatric follow-up.<br><br>NAAT placenta and amniotic fluid at birth; NAAT breast milk and saliva at birth, if evidence of infection.<br><br>TORCH at enrolment / birth, HIV at birth according to local / national guidelines.	Laboratory, pathology examination of fetal loss and stillbirth.	Clinical examination of newborn.<br><br>Newborn testing: NAAT, IgM blood, cord blood; TORCH according to local / national guidelines.	Clinical examination, anthropometry, NAAT blood and urine; IgM, IgG blood at 4w, 4m, 12m, 24m.<br><br>Transcranial U/S at 4w. Hearing, eye, neurodevelopmental assessments.
ZIKAction	IgM, IgG serology booking, 20, 28, Delivery, and at paediatric follow-up<br><br>NAAT testing, and additional U/S investigations if evidence of infection	Laboratory, pathology examination of fetal loss and stillbirth.	Clinical examination of newborn<br><br>Newborn testing: IgM, IgG (serum; CSF only if clinically indicated), urine, saliva. TORCH testing if clinically indicated<br><br>NAAT, U/S and ophthalmology investigations if evidence of infection	Paediatric testing:<br><br>IgG, IgM (serum, urine, saliva) and clinical examination at: 4w, 4m, 9m, 12m, 18m and 24m. NAAT if evidence on infection<br><br>Neurodevelopmental assessments

Deleted: NAAT on placenta.¶
Deleted:
Deleted: ¶
Deleted: and
Deleted:
Deleted:
Deleted: cord blood,
Deleted: urine;
Deleted: TORCH, HIV
Deleted: ¶
¶
Deleted: pediatric

For peer review only - http://bmjopen.bmj.com/site/about/guidelines.xhtml

ZikaPLAN	NAAT and serologic testing (PRNT, IgM, IgG3) when symptoms reported and at up to three follow-up visits.<br>TORCH, DENV, and CHIKV testing	Not routinely tested.	Clinical examination in early infancy.<br>NAAT testing in the neonatal period in some centres. TORCH testing if clinically indicated	Clinical examination of the child by specialists at 3, 6, 12, 18, 24, 36, and 48m<br>Neurodevelopmental assessments

CSF, Cerebro-spinal fluid; DENV, Dengue virus; CHIKV, Chikungunya virus; HIV, Human Immunodeficiency virus; Ig, Immunoglobulin; NAAT, Nucleic Acid Amplification Test; PRNT, Plaque Reduction Neutralisation Test; TORCH, Toxoplasmosis, Other, Rubella, Cytomegalovirus, Herpes; U/S, Ultrasound; ZIKV, Zika virus

For peer review only

Supplementary Table S2. Illustrative and approximate definitions of key explanatory and outcome variables. The final definitions will be determined by the Joint Diagnostics Group and Joint End-point Review Group.

Maternal Symptoms	For example: rash, fever, headache, joint pain
Maternal Infection in Pregnancy (MIP) status, established only on the basis of maternal testing	Confirmed: NAAT, Seroconversion<br>Suspected: Serological tests of recent infection, including: IgM, IgG3, avidity<br>No Evidence of MIP: Maternal testing protocol was followed, but none of the above were positive.<br>No MIP: As above but with IgG negative at or near time of delivery
Laboratory markers of Congenital Infection (CI)	Present: NAAT or IgM any time in first 7 days;<br>Absent: all other findings
Most likely trimester of onset of maternal infection	The highest available from the following hierarchy:<br>• Date of first NAAT positive test minus average duration of viremia<br>• Seroconversion: Midpoint between last serological negative and last positive<br>• Serological tests of recent infection: Date of first positive
Signs and symptoms compatible with Congenital Zika Syndrome (CZS)	A definition will be prepared by the Joint End-point Review Group, based on best evidence available. *The intention will be to produce a definition that is virtually 100% specific for congenital ZIKV infection in mothers exposed to a ZIKV outbreak, especially if other TORCH infections can be ruled out.*
Other Potentially Zika-related Outcomes (OPZRO)	A definition will be prepared by the Joint End-point Review Group, based on best evidence available.

Ig, Immunoglobulin; NAAT, Nucleic Acid Amplification Test.

Deleted: T

Deleted: *M*

Deleted: T

For peer review only - http://bmjopen.bmj.com/site/about/guidelines.xhtml

Supplementary Table S3. Prospectively ascertained Maternal Infection in Pregnancy (MIP) status and evidence on which it is based. NB: Data from the same woman can appear in more than one of the last four columns

Centre	Total women	Prospectively ascertained MIP status				Evidence for Confirmed or Suspected MIP			
		Confirmed MIP	Suspected MIP	No Evidence of MIP	No MIP	NAAT	Sero-conversion	IgM/ IgG3	PRNT
1									
2									
…									
etc									

Ig, Immunoglobulin; NAAT, Nucleic Acid Amplification Test; PRNT, Plaque Reduction Neutralisation Test.

Supplementary Table S4. Summary of evidence on laboratory and clinical markers of congenital infection (CI) and evidence on which it is based. NB: Data from the same fetus/newborn can appear in more than one column

Deleted: S5

		Markers of congenital infection						Other evidence of congenital infection			
Centre	Total number fetus/newborn	Laboratory or clinical markers of congenital infection	Laboratory markers of congenital infection only	Signs and symptoms compatible with CZS	NAAT	IgM	No markers of congenital infection	Microcephaly	Neurological abnormalities	Ocular abnormalities	Other, to be defined
1											
2											
…											
etc											

CZS, Symptoms compatible with Congenital Zika Syndrome; IgM/G, Immunoglobulin M/G; NAAT, Nucleic Acid Amplification Test; OPZRO, Other potentially Zika-related symptoms; PRNT, Plaque Reduction Neutralisation Test.

For peer review only - http://bmjopen.bmj.com/site/about/guidelines.xhtml

Supplementary Table S5. Distributions of signs and symptoms by laboratory markers of congenital infection and prospectively ascertained maternal infection in pregnancy (MIP) status**.** The breakdown shown is an example. Other examples might be "Termination of pregnancy, Pregnancy loss, Stillbirth, Livebirth"; or "Sensorineural hearing loss (SNHL), No SNHL "

**CENTRE**			
**Sign or symptom in fetus, newborn, or infant**	**MIP with laboratory markers of congenital infection**	**MIP with no laboratory markers of congenital infection**	**No MIP**
Signs and symptoms compatible with Congenital Zika Syndrome (CZS)			
Other potentially Zika-related outcomes (OPZRO)			
Asymptomatic			
**TOTAL = 100%**			

Deleted: ¶

For peer review only - http://bmjopen.bmj.com/site/about/guidelines.xhtml

SUPPLEMENTARY MATERIALS

Supplementary Table S1. Schedule of tests and examinations in pregnancy, at delivery, and during paediatric follow-up, in each consortium.

Consortium	Mother	Fetus	Newborn and neonate	Pediatric follow-up
ZIKAlliance	ZIKV serology IgG & IgM, NAAT blood and urine, at booking, every 4w, at birth, and at paediatric follow-up.<br><br>NAAT placenta and amniotic fluid at birth; NAAT breast milk and saliva at birth, if evidence of infection.<br><br>TORCH at enrolment / birth, HIV at birth according to local / national guidelines.	Laboratory, pathology examination of fetal loss and stillbirth.	Clinical examination of newborn.<br><br>Newborn testing: NAAT, IgM blood, cord blood; TORCH according to local / national guidelines.	Clinical examination, anthropometry, NAAT blood and urine; IgM, IgG blood at 4w, 4m, 12m, 24m.<br><br>Transcranial U/S at 4w. Hearing, eye, neurodevelopmental assessments.
ZIKAction	IgM, IgG serology booking, 20, 28, Delivery, and at paediatric follow-up<br><br>NAAT testing, and additional U/S investigations if evidence of infection	Laboratory, pathology examination of fetal loss and stillbirth.	Clinical examination of newborn<br><br>Newborn testing: IgM, IgG (serum; CSF only if clinically indicated), urine, saliva. TORCH testing if clinically indicated<br><br>NAAT, U/S and ophthalmology investigations if evidence of infection	Paediatric testing:<br><br>IgG, IgM (serum, urine, saliva) and clinical examination at: 4w, 4m, 9m, 12m, 18m and 24m. NAAT if evidence on infection<br><br>Neurodevelopmental assessments

For peer review only - http://bmjopen.bmj.com/site/about/guidelines.xhtml

ZikaPLAN	NAAT and serologic testing (PRNT, IgM, IgG3) when symptoms reported and at up to three follow-up visits.<br>TORCH, DENV, and CHIKV testing	Not routinely tested.	Clinical examination in early infancy.<br>NAAT testing in the neonatal period in some centres. TORCH testing if clinically indicated	Clinical examination of the child by specialists at 3, 6, 12, 18, 24, 36, and 48m<br>Neurodevelopmental assessments

CSF, Cerebro-spinal fluid; DENV, Dengue virus; CHIKV, Chikungunya virus; HIV, Human Immunodeficiency virus; Ig, Immunoglobulin; NAAT, Nucleic Acid Amplification Test; PRNT, Plaque Reduction Neutralisation Test; TORCH, Toxoplasmosis, Other, Rubella, Cytomegalovirus, Herpes; U/S, Ultrasound; ZIKV, Zika virus

For peer review only

For peer review only - http://bmjopen.bmj.com/site/about/guidelines.xhtml

Supplementary Table S2. Illustrative and approximate definitions of key explanatory and outcome variables. The final definitions will be determined by the Joint Diagnostics Group and Joint End-point Review Group.

Maternal Symptoms	For example: rash, fever, headache, joint pain
Maternal Infection in Pregnancy (MIP) status, established only on the basis of maternal testing	Confirmed: NAAT, Seroconversion<br>Suspected: Serological tests of recent infection, including: IgM, IgG3, avidity<br>No Evidence of MIP: Maternal testing protocol was followed, but none of the above were positive.<br>No MIP: As above but with IgG negative at or near time of delivery
Laboratory markers of Congenital Infection (CI)	Present: NAAT or IgM any time in first 7 days;<br>Absent: all other findings
Most likely trimester of onset of maternal infection	The highest available from the following hierarchy:<br>• Date of first NAAT positive test minus average duration of viremia<br>• Seroconversion: Midpoint between last serological negative and last positive<br>• Serological tests of recent infection: Date of first positive
Signs and symptoms compatible with Congenital Zika Syndrome (CZS)	A definition will be prepared by the Joint End-point Review Group, based on best evidence available. *The intention will be to produce a definition that is virtually 100% specific for congenital ZIKV infection in mothers exposed to a ZIKV outbreak, especially if other TORCH infections can be ruled out.*
Other Potentially Zika-related Outcomes (OPZRO)	A definition will be prepared by the Joint End-point Review Group, based on best evidence available.

Ig, Immunoglobulin; NAAT, Nucleic Acid Amplification Test.

For peer review only - http://bmjopen.bmj.com/site/about/guidelines.xhtml

Supplementary Table S3. Prospectively ascertained Maternal Infection in Pregnancy (MIP) status and evidence on which it is based. NB: Data from the same woman can appear in more than one of the last four columns

Centre	Total women	Prospectively ascertained MIP status				Evidence for Confirmed or Suspected MIP			
		Confirmed MIP	Suspected MIP	No Evidence of MIP	No MIP	NAAT	Sero-conversion	IgM/ IgG3	PRNT
1									
2									
…									
etc									

Ig, Immunoglobulin; NAAT, Nucleic Acid Amplification Test; PRNT, Plaque Reduction Neutralisation Test.

Supplementary Table S4. Summary of evidence on laboratory and clinical markers of congenital infection (CI) and evidence on which it is based. NB: Data from the same fetus/newborn can appear in more than one column

		Markers of congenital infection						Other evidence of congenital infection			
Centre	Total number fetus/newborn	Laboratory or clinical markers of congenital infection	Laboratory markers of congenital infection only	Signs and symptoms compatible with CZS	NAAT	IgM	No markers of congenital infection	Microcephaly	Neurological abnormalities	Ocular abnormalities	Other, to be defined
1											
2											
…											
etc											

CZS, Symptoms compatible with Congenital Zika Syndrome; IgM/G, Immunoglobulin M/G; NAAT, Nucleic Acid Amplification Test; OPZRO, Other potentially Zika-related symptoms; PRNT, Plaque Reduction Neutralisation Test.

For peer review only - http://bmjopen.bmj.com/site/about/guidelines.xhtml

Supplementary Table S5. Distributions of signs and symptoms by laboratory markers of congenital infection and prospectively ascertained maternal infection in pregnancy (MIP) status**.** The breakdown shown is an example. Other examples might be "Termination of pregnancy, Pregnancy loss, Stillbirth, Livebirth"; or "Sensorineural hearing loss (SNHL), No SNHL "

**CENTRE**			
**Sign or symptom in fetus, newborn, or infant**	**MIP with laboratory markers of congenital infection**	**MIP with no laboratory markers of congenital infection**	**No MIP**
Signs and symptoms compatible with Congenital Zika Syndrome (CZS)			
Other potentially Zika-related outcomes (OPZRO)			
Asymptomatic			
**TOTAL = 100%**			

For peer review only

For peer review only - http://bmjopen.bmj.com/site/about/guidelines.xhtml

BMJ Open

Zika virus infection in pregnancy: A protocol for the joint analysis of the prospective cohort studies of the ZIKAlliance, ZikaPLAN and ZIKAction consortia

Journal:	*BMJ Open*
Manuscript ID	bmjopen-2019-035307.R2
Article Type:	Protocol
Date Submitted by the Author:	01-Oct-2020
Complete List of Authors:	Ades, A E; University of Bristol<br>Brickley, Elizabeth B.; London School of Hygiene and Tropical Medicine, Department of Infectious Disease Epidemiology<br>Alexander, Neal; London School of Hygiene and Tropical Medicine Faculty of Epidemiology and Population Health<br>Brown, David; 4. Fundacão Oswaldo Cruz, Flavivirus Reference Laboratory<br>Jaenisch, Thomas ; UniversitatsKlinikum Heidelberg, Department of Infectious Diseases, Section Clinical Tropical Medicine;<br>Miranda-Filho, Demócrito de Barros; University of Pernambuco, Faculty of Medical Sciences<br>Pohl, Moritz; 7. Institute of Medical Biometry and Informatics, University of Heidelberg<br>Rosenberger, Kerstin Daniela; UniversitatsKlinikum Heidelberg, Department of Infectious Diseases, Section Clinical Tropical Medicine<br>Soriano-Arandes, Antoni; University Hospital Vall d'Hebron, Paediatric Infectious Diseases and Immunodeficiencies Unit, Department of Pediatrics<br>Thorne, Claire; University College London, Great Ormond Street Institute of Child Health<br>Arraes de Alencar Ximenes, Ricardo; Federal University of Pernambuco, Department of Tropical Medicine<br>Velho Barreto de Araújo, Thalia; Universidade Federal de Pernambuco, Department of Social Medicine<br>Avelino-Silva, Vivian ; Faculdade de Medicina da Universidade de Sao Paulo, Department of Infectious and Parasitic Diseases<br>Bethencourt, Sarah ; Universidad de Carabobo, Facultad de Ciencias de la Salud<br>Borja-Aburto, Victor; Mexican Institute of Social Security<br>Brasil, Patrícia ; Fundacao Oswaldo Cruz, Instituto de pesquisa Clínica Evandro Chagas<br>Christie, C. D. C.; University of the West Indies at Mona, Department of Child and Adolescent Health<br>DE SOUZA, Wayner; Instituto Aggeu Magalhães, Fundação Oswaldo Cruz<br>GOTUZZO H., Jose Eduardo ; Universidad Peruana Cayetano Heredia Instituto de Medicina Tropical Alexander von Humboldt<br>Hoen, Bruno; INSERM Centre d'Investigation Clinique 1424, Centre Hospitalier Universitaire de Pointe-à-Pitre; Centre Hospitalier Universitaire de Pointe-à-Pitre

Koopmans, M; Erasmus Universiteit Rotterdam, Department of Viroscience
Turchi Martelli, Celina Maria; Oswaldo Cruz Foundation , Department of Collective Health, Institute Aggeu Magalhães (CPqAM)
Teixeira, Mauro; Universidade Federal de Minas Gerais
Marques, Ernesto; Instituto Aggeu Magalhães, Fundação Oswaldo Cruz; University of Pittsburgh, Department of Infectious Diseases and Microbiology
Miranda Montoya, Maria Consuelo ; Universidad Industrial de Santander, Facultad de Salud ; Universidad Industrial de Santander, Centro de >Investigaciones Epidemiológicas
Ramos Montarroyos, Ulisses; 26. Instituto de Ciências Biológicas, Universidade de Pernambuco
Moreira, Maria ; Figueira National Institute for Women’s, Children’s and Adolescents Health, Oswaldo Cruz Foundation
Morris, J; University of Florida, Emerging Pathogens Institute
Rockx, Barry; Erasmus Universiteit Rotterdam, Department of Viroscience
SABA VILLARROEL, Paola Mariela ; Centro Nacional de Enfermedades Tropicales, Santa Cruz de la Sierra
SORIA SEGARRA, Carmen ; Universidad Católica de Santiago de Guayaquil; SOSECALI C., Ltda
Tami, Adriana; University Medical Center Groningen, Department of Medical Microbiology and Infection Prevention
Turchi, Marília Dalva; Federal University of Goias, Institute of Tropical Pathology and Public Health
Giaquinto, Carlo; University of Padova Faculty of Medicine and Surgery, Department of Woman’s and Child’s Health
de-Lamballerie, Xavier; Aix-Marseille Université Institut Universitaire de Technologie d'Aix-en-Provence
Wilder-Smith, Annelies; Umeå University

**Primary Subject Heading**:	Global health
Secondary Subject Heading:	Epidemiology, Infectious diseases
Keywords:	EPIDEMIOLOGY, Public health < INFECTIOUS DISEASES, Paediatric infectious disease & immunisation < PAEDIATRICS

SCHOLARONE™
Manuscripts

I, the Submitting Author has the right to grant and does grant on behalf of all authors of the Work (as defined in the below author licence), an exclusive licence and/or a non-exclusive licence for contributions from authors who are: i) UK Crown employees; ii) where BMJ has agreed a CC-BY licence shall apply, and/or iii) in accordance with the terms applicable for US Federal Government officers or employees acting as part of their official duties; on a worldwide, perpetual, irrevocable, royalty-free basis to BMJ Publishing Group Ltd ("BMJ") its licensees and where the relevant Journal is co-owned by BMJ to the co-owners of the Journal, to publish the Work in this journal and any other BMJ products and to exploit all rights, as set out in our licence.

The Submitting Author accepts and understands that any supply made under these terms is made by BMJ to the Submitting Author unless you are acting as an employee on behalf of your employer or a postgraduate student of an affiliated institution which is paying any applicable article publishing charge ("APC") for Open Access articles. Where the Submitting Author wishes to make the Work available on an Open Access basis (and intends to pay the relevant APC), the terms of reuse of such Open Access shall be governed by a Creative Commons licence – details of these licences and which Creative Commons licence will apply to this Work are set out in our licence referred to above.

Other than as permitted in any relevant BMJ Author's Self Archiving Policies, I confirm this Work has not been accepted for publication elsewhere, is not being considered for publication elsewhere and does not duplicate material already published. I confirm all authors consent to publication of this Work and authorise the granting of this licence.

For peer review only

Zika virus infection in pregnancy: A protocol for the joint analysis of the prospective cohort studies of the ZIKAlliance, ZikaPLAN and ZIKAction consortia

A. E. ADES[1], Elizabeth B. BRICKLEY[2], Neal ALEXANDER[3], David BROWN[4], Thomas JAENISCH[5], Demócrito de Barros MIRANDA-FILHO[6], Moritz POHL[7], Kerstin D. ROSENBERGER[5], Antoni SORIANO-ARANDES[8], Claire THORNE[9], Ricardo Arraes de Alencar XIMENES[6,10], Thália Velho Barreto de ARAÚJO[11], Vivian I. AVELINO-SILVA[12], Sarah Esperanza BETHENCOURT CASTILLO[13], Victor Hugo BORJA ABURTO[14], Patricia BRASIL[15], Celia D. C. CHRISTIE[16], Wayner Vieira DE SOUZA[17], Jose Eduardo GOTUZZO H.[18], Bruno HOEN[19,20,21], Marion KOOPMANS[22], Celina Maria Turchi MARTELLI[17], Mauro MARTINS TEIXEIRA[23], Ernesto T. A. MARQUES[17,24], Maria Consuelo MIRANDA[25], Ulisses Ramos MONTARROYOS[26], Maria Elisabeth MOREIRA[27], J Glenn MORRIS[28], Barry ROCKX[22], Paola Mariela SABA VILLARROEL[29], Carmen SORIA SEGARRA[30,31], Adriana TAMI[13,32], Marília Dalva TURCHI[33], Carlo GIAQUINTO[34] Xavier de LAMBALLERIE[35], Annelies WILDER-SMITH[36], on behalf of the EC Zika Consortia Vertical Transmission Study Group

1. Department of Population Health Sciences, Bristol Medical School, University of Bristol, Bristol, United Kingdom
2. Department of Infectious Disease Epidemiology, Faculty of Epidemiology and Population Health, London School of Hygiene & Tropical Medicine, London, United Kingdom
3. MRC Tropical Epidemiology Group, Faculty of Epidemiology and Population Health, London School of Hygiene & Tropical Medicine, London, United Kingdom
4. Fundacão Oswaldo Cruz, Flavivirus Reference Laboratory, Rio de Janeiro, Rio de Janeiro, Brazil
5. Section Clinical Tropical Medicine, Department of Infectious Diseases, Heidelberg University Hospital, Heidelberg, Germany
6. Departamento de Medicina Interna, Universidade de Pernambuco, Recife, Pernambuco, Brazil
7. Institute of Medical Biometry and Informatics, University of Heidelberg, Heidelberg, Germany
8. Pediatric Infectious Diseases and Immunodeficiencies Unit, Hospital Universitari Vall d'Hebron, Vall d'Hebron Research Institute, Barcelona, Spain
9. Great Ormond Street Institute of Child Health, University College London, United Kingdom
10. Departamento de Medicina Tropical, Universidade Federal de Pernambuco, Recife, Pernambuco, Brazil
11. Departamento de Medicina Social, Universidade Federal de Pernambuco, Recife, Pernambuco, Brazil
12. Department of Infectious and Parasitic Diseases and Instituto de Medicina Tropical, Faculdade de Medicina da Universidade de Sao Paulo, Sao Paulo, Brazil
13. Facultad de Ciencias de la Salud, Universidad de Carabobo, Valencia, Venezuela
14. Mexican Institute of Social Security, Mexico City, Mexico
15. Evandro Chagas National Institute of Infectious Diseases, Fundação Oswaldo Cruz, Rio de Janeiro, Rio de Janeiro, Brazil
16. Department of Child and Adolescent Health, University of the West Indies, Mona Campus, Kingston, Jamaica
17. Instituto Aggeu Magalhães, Fundação Oswaldo Cruz, Recife, Pernambuco, Brazil
18. Instituto de Medicina Tropical Alexander von Humboldt, Universidad Peruana Cayetano Heredia, Lima, Peru
19. INSERM Centre d'Investigation Clinique 1424, Centre Hospitalier Universitaire de Pointe-à-Pitre, Guadeloupe, France

For peer review only - http://bmjopen.bmj.com/site/about/guidelines.xhtml

20. Service de Maladies Infectieuses et Tropicales, Dermatologie, Médecine Interne, Centre Hospitalier Universitaire de Pointe-à-Pitre, Guadeloupe, France
21. Université des Antilles et de la Guyane, Faculté de Médecine Hyacinthe Bastaraud, EA 4537, Pointe-à-Pitre, Guadeloupe, France.
22. Department of Viroscience, Erasmus Medical Center, Rotterdam, The Netherlands
23. Universidade Federal de Minas Gerais, Belo Horizonte, Minas Gerais, Brazil
24. Department of Infectious Diseases and Microbiology, University of Pittsburgh, Pittsburgh, Pennsylvania, United States of America
25. Universidad Industrial de Santander, Bucaramanga, Colombia
26. Instituto de Ciências Biológicas, Universidade de Pernambuco, Recife, Pernambuco, Brazil
27. Instituto Fernandes Figueira – Fundação Oswaldo Cruz, Rio de Janeiro, Rio de Janeiro, Brazil
28. Emerging Pathogens Institute, University of Florida, Gainesville, Florida, United States of America
29. Centro Nacional de Enfermedades Tropicales, Santa Cruz de la Sierra, Bolivia
30. Universidad Católica de Santiago de Guayaquil, Ecuador
31. SOSECALI C., Ltda, Guayaquil, Ecuador
32. University of Groningen, University Medical Center Groningen, Department of Medical Microbiology and Infection Prevention, The Netherlands
33. Instituto de Patologia Tropical e Saúde Publica, Universidade Federal de Goiás, Goiânia, Goiás, Brazil.
34. Dipartimento di Salute della Donna e del Bambino, Università degli Studi di Padova, Italy
35. Université *d'*Aix-Marseille, Marseille, France
36. Department of Epidemiology and Global Health, Umeå Universite, Umeå, Sweden

Corresponding author:

Claire Thorne, Population Policy and Practice Research and Teaching Department, University College London Great Ormond Street Institute of Child Health, 30 Guilford Street, London WC1N 1EH, United Kingdom

claire.thorne@ucl.ac.uk

For peer review only

ABSTRACT

Introduction

Zika virus (ZIKV) infection in pregnancy has been associated with microcephaly and severe neurological damage to the fetus. Our aim is to document the risks of adverse pregnancy and birth outcomes and the prevalence of laboratory markers of congenital infection in deliveries to women experiencing ZIKV infection during pregnancy, using data from European Commission-funded prospective cohort studies in 20 centres in 11 countries across Latin America and the Caribbean.

Methods and analysis

We will carry out a centre-by-centre analysis of the risks of adverse pregnancy and birth outcomes, comparing women with confirmed and suspected ZIKV infection in pregnancy to those with no evidence of infection in pregnancy. We will document the proportion of deliveries in which laboratory markers of congenital infection were present. Finally, we will investigate the associations of trimester of maternal infection in pregnancy, presence or absence of maternal symptoms of acute ZIKV infection, and previous flavivirus infections with adverse outcomes and with markers of congenital infection. Centre-specific estimates will be pooled using a two-stage approach.

Ethics and dissemination

Ethical approval was obtained at each centre. Findings will be presented at international conferences and published in peer-reviewed open access journals, and discussed with local public health officials and representatives of the national Ministries of Health, Pan American Health Organization, and World Health Organization involved with ZIKV prevention and control activities.

Keywords

Zika virus; pregnancy; vertical transmission; Congenital Zika Syndrome; microcephaly; meta-analysis

For peer review only - http://bmjopen.bmj.com/site/about/guidelines.xhtml

Strengths and limitations of this study

- This will be a pooled analysis of data from 3 international consortia conducting prospective cohort studies of outcomes following ZIKV infection in pregnancy in 20 centres in 11 countries.
- Standardised definitions of outcomes will provide clarity about the absolute risks of adverse outcomes, which have not been reported consistently in prospective studies so far.
- These studies include a control group of women with no evidence of ZIKV infection in pregnancy, allowing improved estimation of the proportion of adverse events attributable to ZIKV in pregnancy.
- Inferences will be limited by: difficulties in distinguishing between women who did and who did not experience a ZIKV infection in pregnancy, due to the high frequency of mild and asymptomatic infections and the low sensitivity and specificity of diagnostic tools; low diagnostic sensitivity of markers of congenital infection; and outcome data that are not missing at random.

For peer review only

INTRODUCTION

Following the emergence of Zika virus (ZIKV) in Asia and the Pacific in 2013 (1) and the subsequent introduction to Brazil (2), clusters of neonates with severe neurological complications and microcephaly were observed across Latin America. Following recent experiences with the H1N1 influenza pandemic and Ebola outbreak in Western Africa, the need for coordinated international research on ZIKV was quickly recognized. In January 2016, before the World Health Organization (WHO) declared a Public Health Emergency of International Concern (3), the European Commission (EC) issued a funding call to set up a network in Latin America and the Caribbean with the aim of implementing and coordinating urgently required research, while simultaneously contributing to research capacity and preparedness for other emerging infectious diseases. Three consortia were funded: ZIKAlliance (https://zikalliance.tghn.org/) (4,5), ZikaPLAN (6) (https://zikaplan.tghn.org/), and ZIKAction (http://zikaction.org/). All are multidisciplinary international collaborations with active investigations in epidemiology, virology, immunology, diagnostics, mathematical modelling, social science, and animal studies. Each consortium includes its own prospective cohort study of ZIKV in pregnancy and a shared work package that aims to ensure the harmonization of protocols and data sets in order to facilitate a pooled analysis of cohort data. The primary aim of the pooled analysis is to investigate the incidence of adverse outcomes of ZIKV infection in pregnancy, including "congenital infection, microcephaly, Zika congenital syndrome, and other sequelae of ZIKV infection".

The aim of this paper is to present a protocol for this pooled analysis. Data has been or is still being collected in multiple sites in 20 regional coordinating centres spread over 11 countries and regions across Latin America and the Caribbean. There are 15 ZIKAlliance centres: Sao Paulo, Rio de Janeiro, Recife and Belo Horizonte (Brazil); Valencia (Venezuela); Bucaramanga (Colombia); Guayaquil (Ecuador); Lima (Peru); Jalisco, Nayarit, Veracruz, Yucatan (Mexico); Santa Cruz de la Sierra (Bolivia); Havana (Cuba); Guadeloupe (French Territory of the Americas); 3 ZikaPLAN centres: Goiânia, Rio de Janeiro, Recife (Brazil); and 2 ZIKAction centres: Kingston (Jamaica) and Port-au-Prince (Haiti). Recruitment to ZIKAlliance began May 2017, December 2015 for ZikaPLAN , and September 2017 for ZIKAction. Over 700 women with confirmed infection had been recruited by April 2020; recruitment continues in some centres.

Several studies of ZIKV in pregnancy have recently been published. In registry-based studies (7-9), fetuses and newborns of women with confirmed infection in pregnancy have been reported to have "potentially Zika-related" adverse outcomes at rates of up to 15%, with higher risk of Zika-associated adverse outcomes in the first trimester. Registry-based studies are likely to over-estimate the risk of severe clinical

For peer review only - http://bmjopen.bmj.com/site/about/guidelines.xhtml

manifestations and underestimate the risk of more mild clinical presentations because they recruit both prospectively ascertained ZIKV-infected pregnant women and women whose infection was recognized retrospectively following the birth of an infant with congenital abnormalities. Prospective studies of congenital infection have variously reported 25% “severe” and 21% “mild to moderate” outcomes in French Guiana (10), and 27% adverse outcomes in Brazil (11). The specificity of these outcome definitions for ZIKV in pregnancy is not known as these studies did not include a control group of women with no ZIKV infection in pregnancy. In another Brazilian study, the risk of adverse outcomes was reported to be 46% in births to women with NAAT (Nucleic Acid Amplification Test)-confirmed ZIKV infection in pregnancy compared to 11.5% in NAAT-negative women (12). In a large prospective study based in the French Territories of the Americas, among infants born to women with NAAT-confirmed ZIKV infection, 7.0% presented with neurologic or ocular birth defects and 3.1% met the study’s criteria for Congenital Zika Syndrome (CZS) (13), which is characterized by several unique features (14). An important limitation in the comparison of the results of these different studies is the lack of a standard definition of CZS and of the clinical and diagnostic procedures used to evaluate these children, leading to possible misclassification of the outcomes studied.

The vertical transmission rate is the probability of congenital infection in births to women with infection in pregnancy. The rates reported so far, 26% (11) and 35% (10), are based on laboratory markers of congenital infection such as NAAT or IgM in the fetus or newborn. However, a prospective cohort retrospectively reconstructed from a register study estimated the vertical transmission rate to be only 9% (15). Comparison of these rates is difficult as different markers and different biological samples were used. In addition, although these tests are analytically sensitive and specific they have poor diagnostic sensitivity as markers of congenital infection. These markers were absent from serum in a high proportion of CZS cases (16) (16,17) and in newborns with other potentially ZIKV-related adverse outcomes born to women with confirmed ZIKV during pregnancy (10,11). Clearance of virus from amniotic fluid and fetal blood has been reported in cases of CZS, even when ZIKV is found in brain tissue post-mortem (18,19). It therefore appears that fetal infection may occur, causing profound damage, but clearing before delivery and leaving no discernable immunological trace in serum. Consequently, in this study we will document the prevalence of markers of congenital infection using uniform criteria, recognizing that this is an underestimate of the true vertical transmission rate.

Regarding effect modifiers, a number of studies have reported a higher incidence of congenital abnormalities following maternal infections in the first trimester (7,10–11,14). Maternal symptoms during

For peer review only - http://bmjopen.bmj.com/site/about/guidelines.xhtml

acute ZIKV infection do not appear to be a risk factor for adverse outcomes (20). There is evidence of antibody dependent enhancement of ZIKV by dengue virus (DENV) antibody in animal models (21), but it is unclear whether previous DENV infection or exposure to other flaviviruses has a protective, risk-enhancing, or null effect, in maternal or congenital infection in humans (22). It also remains to be established whether a previous ZIKV infection confers protective immunity. Little is currently known about risk factors for trans-placental transmission of ZIKV.

The analysis plan described here complements the recently published protocol of the Zika Virus Individual Participant Data (IPD) Consortium (23), which will eventually include data from the three EC consortia as well as data from many other sources. Although the objectives of the protocols are similar, different methods are proposed in relation to design of included studies, definition of congenital infection, and approach to imperfect diagnosis of maternal infection.

In light of the unexplained heterogeneity in reported rates of adverse outcomes, and the variation in prevalence of markers of congenital infection, a pooled analysis of data from 20 centers following similar protocols with harmonized definitions of clinical and laboratory outcomes will provide important new information on outcomes of ZIKV in pregnancy.

OBJECTIVES OF THE JOINT ANALYSIS

1. To estimate the risk of adverse outcomes in the fetus, newborn, and child following maternal ZIKV infection in pregnancy, compared to outcomes in controls with no evidence of maternal infection in pregnancy.
2. To estimate the prevalence of markers of congenital infection among fetuses and liveborn infants following maternal ZIKV infection during pregnancy.
3. To assess the associations between trimester of maternal infection, presence or absence of maternal symptoms, and previous flavivirus infections with adverse outcomes and markers of congenital infection.

METHODS

Participants

For peer review only - http://bmjopen.bmj.com/site/about/guidelines.xhtml

Pregnant women were eligible only if their infection status during pregnancy (infected or not infected) was ascertained prior to the detection of adverse outcomes, or was not influenced by fetal examination or outcome on delivery. This definition is compatible with retrospective testing of previously collected maternal samples, after delivery. Although the unit of recruitment is the mother, the unit of analysis is the fetus, newborn, and infant; multiple births are sufficiently rare to be treated as independent observations (24).

Study design

Consenting women were screened in pregnancy for markers of ZIKV infection. Those in whom maternal infection in pregnancy (MIP) was suspected were followed with enhanced investigations. In ZIKAction and ZIKAlliance, all deliveries to these women, including fetal losses, stillbirths, and newborns were examined clinically and tested for markers of congenital infection. This testing was not routinely performed in ZikaPLAN. In all three cohorts, newborns were prospectively followed to identify any adverse outcomes that may develop later. In all three consortia, a sample of newborns delivered to women with no evidence of infection in pregnancy served as an unexposed control group.

There were some differences between the protocols adopted by the three consortia in terms of how women were recruited into the study, and the choice and scheduling of tests and investigations (Supplementary Table S1). In ZIKAction and ZIKAlliance, women were recruited regardless of symptoms during pregnancy, although report of symptoms was recorded. In ZikaPLAN, only women with rash, a common sign of ZIKV infection, were recruited. Statistical analyses will therefore be stratified by whether the mother reported symptoms in pregnancy. .

When the studies were designed, there was little information on the risk of adverse outcomes of ZIKV in pregnancy, on vertical transmission rates, nor on what infection rates among pregnant women might be expected. Formal sample size calculations were not undertaken.

Target parameters and terminology of vertical transmission studies

Six categories of joint congenital infection status and maternal infection status (A-F) are defined in Table 1, which illustrates the logic of an idealised prospective study. The usual target parameters are the *vertical transmission rate*, which is the probability of congenital infection following MIP, (A+B) / (A+B+C+D); and the *rate of adverse outcomes in those with congenital infection,* A/(A+B). The definitions of “adverse

For peer review only - http://bmjopen.bmj.com/site/about/guidelines.xhtml

outcomes", congenital infection, and MIP will be determined by a Joint Diagnostics Group and a Joint Endpoint Review Group, after the data have been assembled. Estimates of these parameters are standard in the classic studies of vertical transmission of human immune-deficiency virus (HIV) (25,26), toxoplasmosis (27–29) and cytomegalovirus (CMV) (30). In studies of less specific outcomes, the event rate C/(C+D) in fetuses and newborns of women with MIP but in whom no congenital infection occurred (Paediatric Control Group 1 in Table 1) forms a comparison group (31,32) representing the adverse event rate that is due to MIP in the absence of congenital infection. The present analysis plan is modelled closely on these earlier studies, but includes adaptations to take account of the difficulties in diagnosing maternal and congenital ZIKV infection.

For example, because cases of congenital infection cannot be reliably identified by diagnostic tests, we can only estimate the prevalence of laboratory *markers of vertical infection* (Objective 2). Similarly, the "overall" (unconditional) adverse event rate is taken as the primary outcome for Objective 1; this includes all births to women with MIP, (A+C)/(A+B+C+D) (Table 1). All three consortia included a further control group of births to unexposed women, those with no infection in pregnancy (Maternal Control Group in Table 1). The adverse event rates in this group, E/(E+F), represents a baseline for comparison with the overall event rates in exposed women (33).

Although estimates of the vertical transmission rate are compromised, it may still be of interest to compare adverse outcome rates in the MIP with congenital infection group (i.e., A/(A+B)), the MIP without congenital infection group (i.e., C/(C+D)), and the No MIP group (i.e., E/(E+F)), as this may provide insight into whether adverse fetal outcomes are associated with MIP in the absence of demonstrable fetal infection.

In addition to the challenges associated with the laboratory definition of congenital infection, it is also difficult to discriminate between pregnancies with MIP and with No MIP as required in analyses based on Table 1. A positive NAAT result or seroconversion during pregnancy are sufficient to confirm MIP. However, even if tested *per protocol,* MIP may be missed due to the narrow window of detection of NAAT tests, perhaps as low as 14 days (34). Tests of recent infection, including IgM, IgG3 or avidity assays, may reflect infection during pregnancy, but may also be the result of infection prior to pregnancy (35), and immunologic cross-reactivity to DENV antibody may need to be ruled out (36). These tests therefore indicate suspected but not confirmed MIP. An IgG negative response in the woman or newborn at or tests, perhaps as low as 14 days (34). Tests of recent infection, including IgM, IgG3 or avidity assays, may reflect infection during pregnancy, but may also be the result of infection prior to pregnancy (35), and

For peer review only - http://bmjopen.bmj.com/site/about/guidelines.xhtml

immunologic cross-reactivity to DENV antibody may need to be ruled out (36). These tests therefore indicate suspected but not confirmed MIP. An IgG negative response in the woman or newborn at or shortly after delivery can be taken as suggestive of No MIP, although the dynamics of ZIKV IgG are not well documented. While a negative IgG is likely to be uncommon in some sites, as some women will have experienced a ZIKV infection prior to pregnancy, it lowers the probability that a ZIKV infection would have occurred in pregnancy. Taking all this together, MIP status will be characterised as “Confirmed”, “Suspected, “No Evidence of MIP" (i.e., all NAAT and IgM tests negative), and "No MIP" (i.e., all NAAT and IgM tests negative AND IgG negative at or shortly after delivery). To maintain the principle of prospective ascertainment, confirmation of congenital infection will have no impact on the mother’s assigned infection status.

Definitions of variables

To support the pooled analyses, a Joint Diagnostics Group consisting of immunologists and virologists and a Joint Endpoint Review Group consisting of paediatricians with experience of congenital ZIKV will be convened to agree on standardized case definitions. The names and qualifications of members of both groups will be published at the time of the joint data analysis, together with rationale and process for their recruitment. Supplementary Table S2 provides some provisional definitions for: MIP (Confirmed, Suspected, No Evidence of MIP, No MIP), laboratory markers of congenital infection (present or absent), signs and symptoms compatible with Congenital Zika Syndrome (CZS), other potentially-Zika-related outcomes (OPZRO), and trimester of MIP. The Diagnostics Group will also have responsibility for definitions of covariates, such as previous flavivirus infection. Definitions of both diagnostic categories and clinical endpoints will be based on the best information available at the time of analysis, and will to the greatest extent possible, be harmonized across consortia and across centres within consortia. As the expert groups will be assembled from representatives of each consortium, they will already have examined the data prior to a joint analysis, so that blinding will not be possible; however, they will devise diagnostic and clinical criteria that can be applied objectively across the three consortia.

An essential aspect of the definitions for MIP status used in the statistical analyses below is that they must be based exclusively on the *prospective* diagnostic testing. For example, although CZS and/or laboratory evidence of congenital infection in the newborn provides compelling evidence of MIP, this would not affect the MIP status as ascertained prospectively. Thus, we expect to observe some newborns with CZS and/or with laboratory markers of congenital infection delivered to women with Suspected MIP, or even those categorized as having No Evidence of MIP.

For peer review only - http://bmjopen.bmj.com/site/about/guidelines.xhtml

STATISTICAL ANALYSIS

Analysis of data from individual centres

Descriptive tables

We will produce descriptive tables providing a breakdown of the numbers in each centre with Confirmed MIP, Suspected MIP, No Evidence of MIP, and No MIP, and the type of evidence on which this is based (e.g., NAAT, seroconversion, tests of recent infection) (Supplementary Table S3). The similar Supplementary Table S4 will document numbers with signs or symptoms compatible with CZS and with markers of congenital infection, and the type of evidence on which this is based, for example NAAT, IgM, or clinical markers (CZS).

Objective 1: Adverse Outcomes

Prospectively ascertained MIP status will be tabulated against overall (i.e., unconditional on congenital infection status) adverse outcomes (Table 2). Various risks can be estimated within each MIP category, including: the risk of signs and symptoms compatible with CZS, the risk of OPZRO, the risk of both combined, of individual signs and symptoms, or of signs and symptoms grouped in clinically (e.g., ophthalmologic defects) or embryologically meaningful ways. Outcomes may be binary (e.g., microcephaly) or continuous (e.g., head circumference), or multi-category (CZS-related outcomes, OPZRO, asymptomatic). As well as congenital anomalies, rates of standard outcomes, in the absence of congenital anomalies, will be documented, including: fetal loss, stillbirth, low birthweight, intra-uterine growth retardation (IUGR), and premature delivery.

The rate of adverse outcomes in the No MIP group represents the study-specific background rate of adverse outcomes (i.e., in the absence of MIP, Control Group 2), to be compared to rates in women with Confirmed MIP. Absolute risks will be estimated as well as risk ratios and risk differences.

Parallel sets of estimates will be calculated in the Suspected MIP and No Evidence of MIP groups, as particular adverse events indicate lack of diagnostic specificity and sensitivity in the testing protocol

For peer review only - http://bmjopen.bmj.com/site/about/guidelines.xhtml

during pregnancy and are therefore informative regarding the effectiveness of the maternal testing protocol in each centre; for example, microcephaly in the No Evidence of MIP group would indicate a lack of sensitivity. These sets of estimates may be pooled, respectively, with adverse outcome risks in Confirmed MIP and No MIP groups in sensitivity analyses.

Objective 2: Laboratory and clinical markers of vertical transmission

Estimates of the prevalence of markers of congenital infection will be produced in all centres with available data for each MIP group except the No MIP group, as the definition of No MIP is not compatible with laboratory markers of congenital infection. For this purpose, the numerator will be the number with markers of congenital infection, and the denominator will be the sum of the numbers with and without markers of congenital infection. Separate estimates will be obtained for: laboratory markers, clinical markers (namely CZS) , and combined laboratory and clinical markers (Table 3). Rates in the No Evidence of MIP group are of interest as they carry information about the diagnostic accuracy of the maternal testing protocol.

Objective 3: Effect of covariates

Separate estimates of the prevalence of adverse pregnancy outcomes, and the probability of laboratory and clinical markers of congenital infection, will be produced for each trimester of maternal infection, and by presence or absence of maternal symptoms.

Analyses of adverse event frequencies and of markers of congenital infection can be extended to include multiple covariates, using logistic regression. These might include potential *effect modifiers,* such as previous arbovirus infection or co-infection, or *confounding factors* such as socio-economic indicators likely to be associated with both arbovirus exposure and adverse outcomes. However, at the time of writing it is not known whether sufficient data will be available for regression analyses.

Secondary objectives

ZIKV infection in pregnancy could lead to adverse pregnancy and birth outcomes either following a congenital infection (cell A in Table 1) or in the absence of congenital infection (equally in cells A and C).

For peer review only - http://bmjopen.bmj.com/site/about/guidelines.xhtml

Although absence of markers of congenital infection does not rule out congenital infection, we might still expect to observe more adverse outcomes in fetuses and newborns with laboratory markers of congenital infection if those outcomes are caused by congenital infection. By contrast, adverse outcomes that are the result of MIP in the absence of congenital infection (Cell E in Table 1) should occur equally with or without laboratory markers of congenital infection. Based on literature on other infections in pregnancy, including dengue virus (37), adverse outcomes associated with MIP in the absence of congenital infection potentially include: fetal loss, stillbirth, prematurity, IUGR, and low birthweight for gestational age (38). The analysis would be based on a tabulation of presence or absence of neonatal symptoms, or sets of symptoms (Supplementary Table S5), and would be stratified by trimester of maternal infection, as this is likely to be associated with the presence of markers of congenital infection and with adverse outcomes.

Missing Covariates

All analyses will be conducted on a “complete case” basis, in the first instance. Methods for handling missing covariates, such as imputation (39), will be considered after the extent and patterns of missing data have been explored.

Combining data across centres

The above analyses will generate a series of centre-specific estimates of proportions, relative risks comparing MIP and No MIP groups, risk-differences, and means of continuous variables, stratified by trimester and maternal clinical presentation. If logistic regression is used to examine effect-modifiers and confounders, further estimates of interaction terms or adjusted estimates can also be produced.

All these estimates can be combined across centres using fixed or random effects models in a “two-stage” meta-analysis. Bayesian Markov chain Monte Carlo methods will be used as this will facilitate the use of exact binomial and multinomial likelihoods, which have a better performance with low and zero cells counts. Vague priors will be employed. Centre-specific random effect estimates will be sampled from beta distributions for binomial outcomes data, Dirichlet distributions for multinomial data, and normal distributions for continuous data. We will report ranges, between-centre standard deviations, mean effects and predictive effects with 95% credible intervals for each estimate.

For peer review only - http://bmjopen.bmj.com/site/about/guidelines.xhtml

In combining estimates from different centres, we will take account of the fact that in ZikaPLAN only women with rash were recruited, so that women with no ZIKV infection may have experienced other exanthematic infections (12), including arbovirus infections such as dengue and chikungunya, which may themselves be associated with adverse outcomes (40,41).

Depending on the results of two-stage analyses, and the completeness of covariate data, an individual patient data one-stage meta-analyses will be considered for each objective, as a secondary or sensitivity analysis, with centre as an additional fixed “intercept” term.

For peer review only

Sensitivity analyses

Definitions of MIP status, ZIKV-related outcomes, and laboratory markers of congenital infection

We will report differences in adverse event rates between Confirmed MIP and Suspected MIP, and between No Evidence of MIP and No MIP. If the differences are small, we will produce results pooling these categories as a sensitivity analysis.

Further, the No Evidence of MIP category can be subdivided into women who were tested *per protocol* and those who may have been tested less completely. The impact of compliance with protocols will be explored, as it is expected to impact on the proportion of women with MIP who are classified as “No evidence of MIP” and, hence, on the probability of observing adverse outcomes in this group. Similarly, we will conduct sensitivity analyses around the definitions of Confirmed and Suspected MIP on advice from the Joint Diagnostics Group.

Alternative sets of estimates will be generated using alternative criteria for CZS-related outcomes and OPZRO, that are more, or less, specific for ZIKV in pregnancy. Similarly, we will explore the impact of varying the laboratory criteria for congenital infection on the advice of the Joint Diagnostics Group.

Independent ascertainment of outcomes

A critical requirement of all these analyses is that the ascertainment of markers of congenital infection status and clinical outcomes in the fetuses and newborns, and developing infant are all independent of

For peer review only - http://bmjopen.bmj.com/site/about/guidelines.xhtml

each other, and also independent of MIP status. For example, ideally the same laboratory testing for congenital infection is carried out regardless of whether the pregnancy outcome is a fetal loss, termination of pregnancy, stillbirth, a case of CZS, or an apparently healthy asymptomatic infant.

These assumptions are difficult, and in certain respects (e.g., first trimester fetal loss and terminations) not possible, to fully implement in practice. To address these inevitable limitations in the analysis, which are expected to impact more on objective 2 than on objective 1, we will carry out exploratory analyses aimed at detecting potential deviations from protocol. For example, the distribution of trimester of MIP should not be associated with prospectively ascertained MIP status. Guided by the results, we will carry out sensitivity analyses that make a range of assumptions about the distribution of missing data, especially data on markers of congenital infection. A series of scenarios will be examined to assess robustness of results to inherent and/or unplanned deviations from the ideal protocols required for unbiased estimation of the target parameters.

DISCUSSION

Risks of CZS and other adverse birth outcomes of ZIKV infection in pregnancy can only be assessed through studies that recruit women whose infection status is prospectively ascertained, or, if retrospective, ascertainment is independent of outcomes. However, reported risks of adverse outcomes even from prospective studies have been highly variable (10–13), as have vertical transmission rates based on laboratory markers of congenital ZIKV infection (10,11,15). An important role for joint analyses of multiple studies is to explore whether this heterogeneity in outcomes can be explained by individual or study-level covariates. To do this, it is essential that incidental sources of variation, such as those arising from differences in outcome reporting or diagnostic testing, are controlled or eliminated as much as possible. One of the most difficult sources of variation between consortia, and between sites within consortia, lies in diagnosis of maternal infection. Our approach is to have an Expert Diagnostics Group produce a harmonised classification of Confirmed MIP, Suspected MIP, No Evidence of MIP, and No MIP, and to compute a range of estimates of the relative effect of maternal infection on outcomes, grouping these in different ways. An analysis based on the Confirmed MIP and No MIP groups alone would be expected to generate the largest estimates of relative effect, because both poor sensitivity and poor specificity will tend to bias effect estimates towards the null.

An alternative proposal (23) in relation to maternal infection status is to treat test sensitivity and specificity as study-level covariates in a meta-regression, but the risk of false positive diagnosis depends

For peer review only - http://bmjopen.bmj.com/site/about/guidelines.xhtml

more on the incidence of ZIKV and cross-reacting antibodies to other arbovirus infections such as dengue than on test specificity. The Zika Virus IPD Consortium protocol differs in two other ways. First it proposes to include surveillance studies, which may result in over-estimating the risk of adverse outcomes due to retrospective ascertainment of infected women following adverse newborn outcomes (7,42). Second, congenital infection is to be defined by clinical and radiological criteria alone.

Other statistical methods may have been developed by the time the data becomes available for these analyses. Whatever form of analysis is adopted, a standardised pooled analysis from three large consortia comprising 20 centres will provide valuable information about these parameters, which will assist in framing a public health response and advice to women who might be exposed in future. It may also throw light on pathologic mechanisms leading to adverse outcomes, which could help in the development of therapeutic or prophylactic interventions.

Patient and Public Involvement

No patients were involved in the development of this protocol

Acknowledgements The authors express their thanks to Tom Byrne for organising the references.

Collaborators Other members of the EC Zika Consortia Vertical Transmission Study Group are: Joshua ANZINGER (Department of Microbiology, Virology Section, University of the West Indies, Mona Campus, Kingston, Jamaica; Global Virus Network, Baltimore, MD, USA), Heather BAILEY (Institute for Global Health, University College London, UK) Valery E M BEAU DE ROCHARS (Emerging Pathogens Institute, University of Florida, Gainesville, Florida, United States of America), Luana DAMASCENO (Evandro Chagas National Institute of Infectious Diseases, Fundação Oswaldo Cruz, Rio de Janeiro, Brazil), Maria de Fatima Pessoa Militão DE ALBUQUERQUE (Instituto Aggeu Magalhães, Fundação Oswaldo Cruz, Recife, Pernambuco, Brazil), Rafael F. O. FRANÇA (Instituto Aggeu Magalhães, Fundação Oswaldo Cruz, Recife, Pernambuco, Brazil), Hugo Lopez GATELL RAMIREZ (Centro de Investigación sobre Enfermedades Infecciosas, Instituto Nacional de Salud Pública, Cuernavaca, Mexico), Adriana GOMEZ (Universidad Industrial de Santander, Bucaramanga, Colombia), Fabiana GORDON (Population Health Sciences, Bristol Medical School, University of Bristol, Bristol, United Kingdom), Maria G GUZMAN (Instituto de Medicina Tropical Pedro Kourí, La Havana, Cuba), Daniel LANG (Fondazione Penta Onlus, São Paulo, São Paulo, Brazil), Rigan LOUIS (Emerging Pathogens Institute, University of Florida, Gainesville, Florida, United States of America), Anyela Lozano (Universidad Industrial de Santander, Bucaramanga, Colombia), Eric

For peer review only - http://bmjopen.bmj.com/site/about/guidelines.xhtml

MARTINEZ (Instituto de Medicina Tropical Pedro Kourí, La Havana, Cuba), Philippe MAYAUD (Department of Clinical Research, London School of Hygiene and Tropical Medicine, London, UK), Ricard ORTIZ SERRANO (Universidad Autónoma de Bucaramanga, Bucaramanga, Colombia), Silvia P. SALGADO (Instituto Nacional de Salud Pública e Investigación, Guyaquil, Ecuador), Aluisio Augusto Cotrim SEGURADO (Department of Infectious and Parasitic Diseases and Instituto de Medicina Tropical, Faculdade de Medicina da Universidade de Sao Paulo, Sao Paulo, Brazil), Zilton F. M. VASCONCELOS (Instituto Fernandes Figueira – Fundação Oswaldo Cruz, Rio de Janeiro, Rio de Janeiro, Brazil), Isabelle Freire Tabosa VIANA (Instituto Aggeu Magalhães, Fundação Oswaldo Cruz, Recife, Pernambuco, Brazil), Luis Angel VILLAR (Universidad Industrial de Santander, Bucaramanga, Colombia)

Contributors A steering group (CT, TJ, KDR, RAAX, AEA, EBB, NA) was formed to prepare a Joint Statistical Analysis Plan pursuant to the terms of the three EC Zika Consortia funding, at the direction of the consortia PIs (CG, XdL and AW-S). After initial discussions the steering group delegated the task to AEA, EBB and NA. The SAP was conceptualised and drafted by AEA with EBB and NA. After several iterations, the draft was revised based on comments from CT, DB, ASA, TJ, KDR, MP, DBM, RAAX, MCM, AT, CG, XdL and AW-S. Comments were then solicited from TVBA, VIAS, SEBC, VHBA, PB, CDCC, WVS, JEGH, BH, MK, CMTM, MMT, ETAM, URM, MEM, JGM, BR, PMSV, CSS and MDT and a revision was drafted by AEA, EBB and NA. The final draft was agreed by all authors.

Funding European Union Directorate-General for Research grant numbers 734548 (ZIKAlliance), 734584 (ZikaPLAN), and 734857 (ZIKAction) Horizon 2020 programme of the European Commission. EBB was also supported by Wellcome Trust & the UK's Department for International Development (205377/Z/16/Z to LCR; https://wellcome.ac.uk/).

Patient consent for publication Not required

Competing interests All authors have completed the ICMJE uniform disclosure form. The following authors report grants from the European Commission (AET, EB, NA, DB, TJ, KDR, CT, CDC, ASA, JGM, CG, XL, AWS); EBB reports funding from by Wellcome Trust & the UK's Department for International Development; MMT reports grants from Conselho Nacional de Desenvolvimento Cientifico e Tecnologico, Brazil and from Fundacao de Amparo a Pesquisa do EStado de MInas Gerais (FAPEMIG, Brazil), during the conduct of the study; MK has a patent on zika diagnostics pending.

For peer review only - http://bmjopen.bmj.com/site/about/guidelines.xhtml

For peer review only

For peer review only - http://bmjopen.bmj.com/site/about/guidelines.xhtml

TABLES

Table 1. Design of an idealized prospective vertical transmission study. The vertical transmission rate is estimated by (A+B)/(A+B+C+D). The rate of adverse outcomes conditional on congenital infection is A/(A+B). This can be compared with the rate of adverse outcomes in newborns with no congenital infection, C/(C+D), who form a control group (Pediatric Control Group) to account for potential confounders associated with maternal infection.

An overall, non-conditional estimate of the adverse event rate is (A+C)/(A+B+C+D).

Follow-up of births to women with no MIP creates a second control group (Maternal Control Group), in which the rate of adverse outcomes, E/(E+F), can be compared to the rate in births with no congenital infection to women with MIP. Estimated effects of MIP based on the Maternal Control Group are vulnerable to confounding by factors associated with MIP. Some cells are set to zero as there can be no congenital infection without MIP.

			**Maternal Infection Status**	
			MIP	No MIP
**Congenital Infection Status**	**congenital infection**	Adverse outcomes	A	0
		No Adverse outcomes	B	0
	**No congenital infection**	Adverse outcomes	C	E
		No Adverse outcomes	D	F

For peer review only

Table 2. Scheme for a generic analysis of risks of adverse outcomes by prospectively ascertained Maternal Infection in Pregnancy (MIP) status. Presence or absence of fetal and neonatal signs or symptoms (e.g., microcephaly, brain calcifications, arthrogryposis) and other Potentially Zika-related outcomes (e.g., fetal loss) in the different MIP groups will be compared.

**CENTRE**	**Maternal Infection in Pregnancy status**			
	Confirmed	Suspected	No Evidence of MIP	No MIP
**Symptom 1**				
**Symptom 2**				
**Symptom 3**				
**:**				
**No Symptoms**				
**TOTAL**				

Table 3. Scheme for generic analysis of markers of congenital infection by prospectively ascertained Maternal Infection in Pregnancy (MIP) status. Markers of congenital infection may include laboratory markers, clinical markers, such as microcephaly, or both combined.

**CENTRE**	**Maternal Infection in Pregnancy status**			
	Confirmed	Suspected	No Evidence of MIP	No MIP
**Both laboratory and clinical markers of congenital infection**				
**Only laboratory markers of congenital infection**				
**Only clinical markers of congenital infection**				

For peer review only - http://bmjopen.bmj.com/site/about/guidelines.xhtml

**No markers of congenital infection**				
**Not tested**				
**TOTAL**				

For peer review only

For peer review only - http://bmjopen.bmj.com/site/about/guidelines.xhtml

References

1. Roth A, Mercier A, Lepers C, Hoy D, Duituturaga S, Benyon E, et al. Concurrent outbreaks of dengue, chikungunya and zika virus infections null An unprecedented epidemic wave of mosquito-borne viruses in the Pacific 2012null2014. Eurosurveillance. 2014;19(41):1–8.
2. Campos GS, Bandeira AC, Sardi SI. Zika Virus Outbreak, Bahia, Brazil. Emerg Infect Dis. 2015;21(10):1885–6.
3. Gulland A. Zika virus is a global public health emergency, declares WHO. BMJ. 2016;352(February):i657.
4. Avelino-Silva VI, Mayaud P, Tami A, Miranda MC, Rosenberger KD, Alexander N, et al. Study protocol for the multicentre cohorts of Zika virus infection in pregnant women, infants, and acute clinical cases in Latin America and the Caribbean: The ZIKAlliance consortium. BMC Infect Dis. 2019;19(1):1–14.
5. De Alencar Ximenes RA, De Barros Miranda-Filho D, Brickley EB, Montarroyos UR, Martelli CMT, de Araújo TVB, et al. Zika virus infection in pregnancy: Establishing a case definition for clinical research on pregnant women with rash in an active transmission setting. PLoS Negl Trop Dis. 2019;13(10):1–17.
6. Wilder-Smith A, Preet R, Renhorn KE, Ximenes RA, Rodrigues LC, Solomon T, et al. ZikaPLAN: Zika Preparedness Latin American Network. Glob Health Action. 2017;10(1):1398485.
7. Reynolds M, Jones A, Petersen E, Lee E, Rice M, Bingham A. Vital Signs: Update on Zika Virus–Associated Birth Defects and Evaluation of All U.S. Infants with Congenital Zika Virus Exposure — U.S. Zika Pregnancy Registry, 2016. Morb Mortal Wkly Rep. 2017;66(13):366–73.
8. Shapiro-Mendoza CK, Rice ME, Galang RR, Fulton AC, VanMaldeghem K, Prado MV, et al. Pregnancy Outcomes After Maternal Zika Virus Infection During Pregnancy — U.S. Territories, January 1, 2016–April 25, 2017. MMWR Morb Mortal Wkly Rep. 2017;66(23):615–21.
9. Honein MA, Dawson AL, Petersen EE, Jones AM, Lee EH, Yazdy MM, et al. Birth defects among fetuses and infants of US women with evidence of possible zika virus infection during pregnancy. JAMA - J Am Med Assoc. 2017;317(1):59–68.
10. Pomar L, Vouga M, Lambert V, Pomar C, Hcini N, Jolivet A, et al. Maternal-fetal transmission and adverse perinatal outcomes in pregnant women infected with Zika virus: prospective cohort study in French Guiana. Bmj. 2018;k4431.
11. Nogueira ML, Nery Júnior NRR, Estofolete CF, Bernardes Terzian AC, Guimarães GF, Zini N, et al. Adverse birth outcomes associated with Zika virus exposure during pregnancy in São José do Rio Preto, Brazil. Clin Microbiol Infect. 2018 Jun;24(6):646–52.
12. Brasil P, Pereira J, Moreira E, Riberio Nogueira RM, Damasceno L, Wakimoto M, et al. Zika Virus Infection in Pregnant Women in Rio de Janeiro. N Engl J Med. 2016;56(11):1024–5.
13. Hoen B, Schaub B, Funk AL, Ardillon V, Boullard M, Cabié A, et al. Pregnancy Outcomes after ZIKV Infection in French Territories in the Americas. N Engl J Med. 2018;378(11):985–94.
14. Moore CA, Staples JE, Dobyns WB, Pessoa A, Ventura C V., Da Fonseca EB, et al. Characterizing the pattern of anomalies in congenital zika syndrome for pediatric clinicians. JAMA Pediatr. 2017;171(3):288–95.
15. Conners EE, Lee EH, Thompson CN, Mcgibbon E, Rakeman JL, Iwamoto M, et al. Zika Virus Infection Among PregnantWomen and Their Neonates in NewYork City, January 2016–June 2017. Obs Gynecol. 2018;132:487–95.
16. van der Linden V, Pessoa A, Dobyns W, Barkovich AJ, Júnior H van der L, Filho ELR, et al. Description of 13 Infants Born During October 2015–January 2016 With Congenital Zika

For peer review only - http://bmjopen.bmj.com/site/about/guidelines.xhtml

Virus Infection Without Microcephaly at Birth — Brazil. Morb Mortal Wkly Rep. 2016;65(47):1343–8.

17. de Araújo TVB, Ximenes RA de A, Miranda-Filho D de B, Souza WV, Montarroyos UR, de Melo APL, et al. Association between microcephaly, Zika virus infection, and other risk factors in Brazil: final report of a case-control study. Lancet Infect Dis. 2018 Mar;18(3):328–36.
18. Schaub B, Vouga M, Najioullah F, Gueneret M, Monthieux A, Harte C, et al. Analysis of blood from Zika virus-infected fetuses: a prospective case series. Lancet Infect Dis. 2017;17(5):520–7.
19. Rodó C, Suy A, Sulleiro E, Soriano-Arandes A, Antón A, García-Ruiz I, et al. In utero negativization of Zika virus in a foetus with serious central nervous system abnormalities. Clin Microbiol Infect. 2018;24(5):549.e1-549.e3.
20. Paixao ES, Leong WY, Rodrigues LC, Wilder-Smith A. Asymptomatic prenatal Zika virus infection and congenital Zika syndrome. Open Forum Infect Dis. 2018;5(4):1–5.
21. Bardina S V., Bunduc P, Tripathi S, Duehr J, Frere JJ, Brown JA, et al. Enhancement of Zika virus pathogenesis by preexisting antiflavivirus immunity. Science (80-). 2017;356(6334):175–80.
22. Masel J, McCracken MK, Gleeson T, Morrison B, Rutherford G, Imrie A, et al. Does prior dengue virus exposure worsen clinical outcomes of Zika virus infection? A systematic review, pooled analysis and lessons learned. PLoS Negl Trop Dis. 2019;13(1):1–14.
23. Wilder-Smith A, Wei Y, Araújo TVB de, VanKerkhove M, Turchi Martelli CM, Turchi MD, et al. Understanding the relation between Zika virus infection during pregnancy and adverse fetal, infant and child outcomes: a protocol for a systematic review and individual participant data meta-analysis of longitudinal studies of pregnant women and their inf. BMJ Open. 2019;9(6):e026092.
24. Thorne C, Malyuta R, Semenenko I, Pilipenko T, Patel D, Bunders M, et al. The mother-to-child HIV transmission epidemic in Europe: Evolving in the East and established in the West. Aids. 2006;20(10):1419–27.
25. Mandelbrot, Mayaux, Bongain, Berrebi, Moudoub-Jeanpetit, Bénifla, et al. Obstetric factors and mother-to-child transmission of human immunodeficiency virus type 1: The French perinatal cohorts. Am J Obstet Gynecol. 1996;175(3):661–7.
26. Dunn DT, Ades AE. Estimating the HIV Vertical Transmission Rate and the Pediatric AIDS Incubation Period from Prospective Data AU. J Am Stat Assoc. 1996 Sep 1;91(435):935–43.
27. Gras L, Gilbert RE, Ades AE, Dunn DT. Effect of prenatal treatment on the risk of intracranial and ocular lesions in children with congenital toxoplasmosis. Int J Epidemiol. 2001;30(6):1309–13.
28. Gilbert RE, Gras L, Wallon M, Peyron F, Ades AE, Dunn DT. Effect of prenatal treatment on mother to child transmission of Toxoplasma gondii: Retrospective cohort study of 554 mother-child pairs in Lyon, France. Int J Epidemiol. 2001;30(6):1303–8.
29. The SYROCOT (Systematic Review on Congenital Toxoplasmosis) study group. Eff ectiveness of prenatal treatment for congenital toxoplasmosis: a meta-analysis of individual patients' data. Lancet. 2007;369(9556):115–22.
30. Stagno S, Pass RF, Dworsky ME, Henderson RE, Moore EG, Walton PD, et al. Congenital Cytomegalovirus Infection: The relative importance of primary and recurrent maternal infection. N Engl J Med. 1982 Apr;306(16):945–9.
31. Townsend CL, Forsgren M, Ahlfors K, Ivarsson SA, Tookey PA, Peckham CS. Long-term outcomes of congenital cytomegalovirus infection in Sweden and the United Kingdom. Clin Infect Dis. 2013;56(9):1232–9.

For peer review only - http://bmjopen.bmj.com/site/about/guidelines.xhtml

32. Preece PM, Blount JM, Glover J, Fletcher GM, Peckham CS, Griffiths PD. The consequences of primary cytomegalovirus infection in pregnancy. Arch Dis Child. 1983;58(12):970–5.
33. Li XL, Wei HX, Zhang H, Peng HJ, Lindsay DS. A meta analysis on risks of adverse pregnancy outcomes in Toxoplasma gondii infection. PLoS One. 2014;9(5):1–12.
34. Paz-Bailey G, Rosenberg ES, Doyle K, Munoz-Jordan J, Santiago GA, Klein L, et al. Persistence of Zika Virus in Body Fluids — Final Report. N Engl J Med. 2018;NEJMoa1613108.
35. Centers for Disease Control and Prevention. Prolonged IgM Antibody Response in People Infected with Zika Virus : Implications for Interpreting Serologic Testing Results for Pregnant. 2017;5–7.
36. Rabe IB, Staples JE, Villanueva J, Hummel KB, Johnson JA, Rose L, et al. Interim Guidance for Interpretation of Zika Virus Antibody Test Results. MMWR Morb Mortal Wkly Rep. 2016;65(21):543–6.
37. Paixão ES, Teixeira MG, Costa M da CN, Barreto ML, Rodrigues LC. Symptomatic Dengue during Pregnancy and Congenital Neurologic Malformations. Emerg Infect Dis. 2018 Sep;24(9):1748–50.
38. Cardenas I, Means RE, Aldo P, Koga K, Lang SM, Booth C, et al. Viral Infection of the Placenta Leads to Fetal Inflammation and Sensitization to Bacterial Products Predisposing to Preterm Labor. J Immunol. 2010;185(2):1248–57.
39. JL. S. Multiple imputation: a primer. Stat Methods Med Res. 1999;8(1):3–15.
40. Basurko C, Matheus S, Hildéral H, Everhard S, Restrepo M, Cuadro-Alvarez E, et al. Estimating the risk of vertical transmission of dengue: A prospective study. Am J Trop Med Hyg. 2018;98(6):1826–32.
41. Contopoulos-Ioannidis D, Newman-Lindsay S, Chow C, LaBeaud AD. Mother-to-child transmission of Chikungunya virus: A systematic review and meta-analysis. PLoS Negl Trop Dis. 2018;12(6):1–20.
42. Honein MA, Dawson AL, Petersen EE, Jones AM, Lee EH, Yazdy MM, et al. Birth defects among fetuses and infants of US women with evidence of possible zika virus infection during pregnancy. JAMA - J Am Med Assoc. 2017 Jan 3;317(1):59–68.

For peer review only

SUPPLEMENTARY MATERIALS

Supplementary Table S1. Schedule of tests and examinations in pregnancy, at delivery, and during paediatric follow-up, in each consortium.

Consortium	Mother	Fetus	Newborn and neonate	Pediatric follow-up
ZIKAlliance	ZIKV serology IgG & IgM, NAAT blood and urine, at booking, every 4w, at birth, and at paediatric follow-up.<br><br>NAAT placenta and amniotic fluid at birth; NAAT breast milk and saliva at birth, if evidence of infection.<br><br>TORCH at enrolment / birth, HIV at birth according to local / national guidelines.	Laboratory, pathology examination of fetal loss and stillbirth.	Clinical examination of newborn.<br><br>Newborn testing: NAAT, IgM blood, cord blood; TORCH according to local / national guidelines.	Clinical examination, anthropometry, NAAT blood and urine; IgM, IgG blood at 4w, 4m, 12m, 24m.<br><br>Transcranial U/S at 4w. Hearing, eye, neurodevelopmental assessments.
ZIKAction	IgM, IgG serology booking, 20, 28, Delivery, and at paediatric follow-up<br><br>NAAT testing, and additional U/S investigations if evidence of infection	Laboratory, pathology examination of fetal loss and stillbirth.	Clinical examination of newborn<br><br>Newborn testing: IgM, IgG (serum; CSF only if clinically indicated), urine, saliva. TORCH testing if clinically indicated<br><br>NAAT, U/S and ophthalmology investigations if evidence of infection	Paediatric testing:<br><br>IgG, IgM (serum, urine, saliva) and clinical examination at: 4w, 4m, 9m, 12m, 18m and 24m. NAAT if evidence on infection<br><br>Neurodevelopmental assessments

For peer review only - http://bmjopen.bmj.com/site/about/guidelines.xhtml

ZikaPLAN	NAAT and serologic testing (PRNT, IgM, IgG3) when symptoms reported and at up to three follow-up visits.<br><br>TORCH, DENV, and CHIKV testing	Not routinely tested.	Clinical examination in early infancy.<br><br>NAAT testing in the neonatal period in some centres. TORCH testing if clinically indicated	Clinical examination of the child by specialists at 3, 6, 12, 18, 24, 36, and 48m<br><br>Neurodevelopmental assessments

CSF, Cerebro-spinal fluid; DENV, Dengue virus; CHIKV, Chikungunya virus; HIV, Human Immunodeficiency virus; Ig, Immunoglobulin; NAAT, Nucleic Acid Amplification Test; PRNT, Plaque Reduction Neutralisation Test; TORCH, Toxoplasmosis, Other, Rubella, Cytomegalovirus, Herpes; U/S, Ultrasound; ZIKV, Zika virus

For peer review only

Supplementary Table S2. Illustrative and approximate definitions of key explanatory and outcome variables. The final definitions will be determined by the Joint Diagnostics Group and Joint End-point Review Group.

Maternal Symptoms	For example: rash, fever, headache, joint pain
Maternal Infection in Pregnancy (MIP) status, established only on the basis of maternal testing	Confirmed: NAAT, Seroconversion<br>Suspected: Serological tests of recent infection, including: IgM, IgG3, avidity<br>No Evidence of MIP: Maternal testing protocol was followed, but none of the above were positive.<br>No MIP: As above but with IgG negative at or near time of delivery
Laboratory markers of Congenital Infection (CI)	Present: NAAT or IgM any time in first 7 days;<br>Absent: all other findings
Most likely trimester of onset of maternal infection	The highest available from the following hierarchy:<br>• Date of first NAAT positive test minus average duration of viremia<br>• Seroconversion: Midpoint between last serological negative and last positive<br>• Serological tests of recent infection: Date of first positive
Signs and symptoms compatible with Congenital Zika Syndrome (CZS)	A definition will be prepared by the Joint End-point Review Group, based on best evidence available. *The intention will be to produce a definition that is virtually 100% specific for congenital ZIKV infection in mothers exposed to a ZIKV outbreak, especially if other TORCH infections can be ruled out.*
Other Potentially Zika-related Outcomes (OPZRO)	A definition will be prepared by the Joint End-point Review Group, based on best evidence available.

Ig, Immunoglobulin; NAAT, Nucleic Acid Amplification Test.

For peer review only - http://bmjopen.bmj.com/site/about/guidelines.xhtml

Supplementary Table S3. Prospectively ascertained Maternal Infection in Pregnancy (MIP) status and evidence on which it is based. NB: Data from the same woman can appear in more than one of the last four columns

Centre	Total women	Prospectively ascertained MIP status				Evidence for Confirmed or Suspected MIP			
		Confirmed MIP	Suspected MIP	No Evidence of MIP	No MIP	NAAT	Sero-conversion	IgM/ IgG3	PRNT
1									
2									
…									
etc									

Ig, Immunoglobulin; NAAT, Nucleic Acid Amplification Test; PRNT, Plaque Reduction Neutralisation Test.

Supplementary Table S4. Summary of evidence on laboratory and clinical markers of congenital infection (CI) and evidence on which it is based. NB: Data from the same fetus/newborn can appear in more than one column

		Markers of congenital infection						Other evidence of congenital infection			
Centre	Total number fetus/newborn	Laboratory or clinical markers of congenital infection	Laboratory markers of congenital infection only	Signs and symptoms compatible with CZS	NAAT	IgM	No markers of congenital infection	Microcephaly	Neurological abnormalities	Ocular abnormalities	Other, to be defined
1											
2											
…											
etc											

CZS, Symptoms compatible with Congenital Zika Syndrome; IgM/G, Immunoglobulin M/G; NAAT, Nucleic Acid Amplification Test; OPZRO, Other potentially Zika-related symptoms; PRNT, Plaque Reduction Neutralisation Test.

For peer review only - http://bmjopen.bmj.com/site/about/guidelines.xhtml

Supplementary Table S5. Distributions of signs and symptoms by laboratory markers of congenital infection and prospectively ascertained maternal infection in pregnancy (MIP) status**.** The breakdown shown is an example. Other examples might be "Termination of pregnancy, Pregnancy loss, Stillbirth, Livebirth"; or "Sensorineural hearing loss (SNHL), No SNHL "

**CENTRE**			
**Sign or symptom in fetus, newborn, or infant**	**MIP with laboratory markers of congenital infection**	**MIP with no laboratory markers of congenital infection**	**No MIP**
Signs and symptoms compatible with Congenital Zika Syndrome (CZS)			
Other potentially Zika-related outcomes (OPZRO)			
Asymptomatic			
**TOTAL = 100%**			

For peer review only

BMJ Open

Zika virus infection in pregnancy: A protocol for the joint analysis of the prospective cohort studies of the ZIKAlliance, ZikaPLAN and ZIKAction consortia

Journal:	*BMJ Open*
Manuscript ID	bmjopen-2019-035307.R3
Article Type:	Protocol
Date Submitted by the Author:	10-Nov-2020
Complete List of Authors:	Ades, A E; University of Bristol<br>Brickley, Elizabeth B.; London School of Hygiene and Tropical Medicine, Department of Infectious Disease Epidemiology<br>Alexander, Neal; London School of Hygiene and Tropical Medicine Faculty of Epidemiology and Population Health<br>Brown, David; 4. Fundacão Oswaldo Cruz, Flavivirus Reference Laboratory<br>Jaenisch, Thomas ; UniversitatsKlinikum Heidelberg, Department of Infectious Diseases, Section Clinical Tropical Medicine;<br>Miranda-Filho, Demócrito de Barros; University of Pernambuco, Faculty of Medical Sciences<br>Pohl, Moritz; 7. Institute of Medical Biometry and Informatics, University of Heidelberg<br>Rosenberger, Kerstin Daniela; UniversitatsKlinikum Heidelberg, Department of Infectious Diseases, Section Clinical Tropical Medicine<br>Soriano-Arandes, Antoni; University Hospital Vall d'Hebron, Paediatric Infectious Diseases and Immunodeficiencies Unit, Department of Pediatrics<br>Thorne, Claire; University College London, Great Ormond Street Institute of Child Health<br>Arraes de Alencar Ximenes, Ricardo; Federal University of Pernambuco, Department of Tropical Medicine<br>Velho Barreto de Araújo, Thalia; Universidade Federal de Pernambuco, Department of Social Medicine<br>Avelino-Silva, Vivian ; Faculdade de Medicina da Universidade de Sao Paulo, Department of Infectious and Parasitic Diseases<br>Bethencourt, Sarah ; Universidad de Carabobo, Facultad de Ciencias de la Salud<br>Borja-Aburto, Victor; Mexican Institute of Social Security<br>Brasil, Patrícia ; Fundacao Oswaldo Cruz, Instituto de pesquisa Clínica Evandro Chagas<br>Christie, C. D. C.; University of the West Indies at Mona, Department of Child and Adolescent Health<br>DE SOUZA, Wayner; Instituto Aggeu Magalhães, Fundação Oswaldo Cruz<br>GOTUZZO H., Jose Eduardo ; Universidad Peruana Cayetano Heredia Instituto de Medicina Tropical Alexander von Humboldt<br>Hoen, Bruno; INSERM Centre d'Investigation Clinique 1424, Centre Hospitalier Universitaire de Pointe-à-Pitre; Centre Hospitalier Universitaire de Pointe-à-Pitre

Koopmans, M; Erasmus Universiteit Rotterdam, Department of Viroscience
Turchi Martelli, Celina Maria; Oswaldo Cruz Foundation , Department of Collective Health, Institute Aggeu Magalhães (CPqAM)
Teixeira, Mauro; Universidade Federal de Minas Gerais
Marques, Ernesto; Instituto Aggeu Magalhães, Fundação Oswaldo Cruz; University of Pittsburgh, Department of Infectious Diseases and Microbiology
Miranda Montoya, Maria Consuelo ; Universidad Industrial de Santander, Facultad de Salud ; Universidad Industrial de Santander, Centro de >Investigaciones Epidemiológicas
Ramos Montarroyos, Ulisses; 26. Instituto de Ciências Biológicas, Universidade de Pernambuco
Moreira, Maria ; Figueira National Institute for Women’s, Children’s and Adolescents Health, Oswaldo Cruz Foundation
Morris, J; University of Florida, Emerging Pathogens Institute
Rockx, Barry; Erasmus Universiteit Rotterdam, Department of Viroscience
SABA VILLARROEL, Paola Mariela ; Centro Nacional de Enfermedades Tropicales, Santa Cruz de la Sierra
SORIA SEGARRA, Carmen ; Universidad Católica de Santiago de Guayaquil; SOSECALI C., Ltda
Tami, Adriana; University Medical Center Groningen, Department of Medical Microbiology and Infection Prevention
Turchi, Marília Dalva; Federal University of Goias, Institute of Tropical Pathology and Public Health
Giaquinto, Carlo; University of Padova Faculty of Medicine and Surgery, Department of Woman’s and Child’s Health
de-Lamballerie, Xavier; Aix-Marseille Université Institut Universitaire de Technologie d'Aix-en-Provence
Wilder-Smith, Annelies; Umeå University

**Primary Subject Heading**:	Global health
Secondary Subject Heading:	Epidemiology, Infectious diseases
Keywords:	EPIDEMIOLOGY, Public health < INFECTIOUS DISEASES, Paediatric infectious disease & immunisation < PAEDIATRICS

SCHOLARONE™
Manuscripts

I, the Submitting Author has the right to grant and does grant on behalf of all authors of the Work (as defined in the below author licence), an exclusive licence and/or a non-exclusive licence for contributions from authors who are: i) UK Crown employees; ii) where BMJ has agreed a CC-BY licence shall apply, and/or iii) in accordance with the terms applicable for US Federal Government officers or employees acting as part of their official duties; on a worldwide, perpetual, irrevocable, royalty-free basis to BMJ Publishing Group Ltd ("BMJ") its licensees and where the relevant Journal is co-owned by BMJ to the co-owners of the Journal, to publish the Work in this journal and any other BMJ products and to exploit all rights, as set out in our licence.

The Submitting Author accepts and understands that any supply made under these terms is made by BMJ to the Submitting Author unless you are acting as an employee on behalf of your employer or a postgraduate student of an affiliated institution which is paying any applicable article publishing charge ("APC") for Open Access articles. Where the Submitting Author wishes to make the Work available on an Open Access basis (and intends to pay the relevant APC), the terms of reuse of such Open Access shall be governed by a Creative Commons licence – details of these licences and which Creative Commons licence will apply to this Work are set out in our licence referred to above.

Other than as permitted in any relevant BMJ Author's Self Archiving Policies, I confirm this Work has not been accepted for publication elsewhere, is not being considered for publication elsewhere and does not duplicate material already published. I confirm all authors consent to publication of this Work and authorise the granting of this licence.

For peer review only

Zika virus infection in pregnancy: A protocol for the joint analysis of the prospective cohort studies of the ZIKAlliance, ZikaPLAN and ZIKAction consortia

A. E. ADES[1], Elizabeth B. BRICKLEY[2], Neal ALEXANDER[3], David BROWN[4], Thomas JAENISCH[5], Demócrito de Barros MIRANDA-FILHO[6], Moritz POHL[7], Kerstin D. ROSENBERGER[5], Antoni SORIANO-ARANDES[8], Claire THORNE[9], Ricardo Arraes de Alencar XIMENES[6,10], Thália Velho Barreto de ARAÚJO[11], Vivian I. AVELINO-SILVA[12], Sarah Esperanza BETHENCOURT CASTILLO[13], Victor Hugo BORJA ABURTO[14], Patricia BRASIL[15], Celia D. C. CHRISTIE[16], Wayner Vieira DE SOUZA[17], Jose Eduardo GOTUZZO H.[18], Bruno HOEN[19,20,21], Marion KOOPMANS[22], Celina Maria Turchi MARTELLI[17], Mauro MARTINS TEIXEIRA[23], Ernesto T. A. MARQUES[17,24], Maria Consuelo MIRANDA[25], Ulisses Ramos MONTARROYOS[26], Maria Elisabeth MOREIRA[27], J Glenn MORRIS[28], Barry ROCKX[22], Paola Mariela SABA VILLARROEL[29], Carmen SORIA SEGARRA[30,31], Adriana TAMI[13,32], Marília Dalva TURCHI[33], Carlo GIAQUINTO[34] Xavier de LAMBALLERIE[35], Annelies WILDER-SMITH[36], on behalf of the EC Zika Consortia Vertical Transmission Study Group

1. Department of Population Health Sciences, Bristol Medical School, University of Bristol, Bristol, United Kingdom
2. Department of Infectious Disease Epidemiology, Faculty of Epidemiology and Population Health, London School of Hygiene & Tropical Medicine, London, United Kingdom
3. MRC Tropical Epidemiology Group, Faculty of Epidemiology and Population Health, London School of Hygiene & Tropical Medicine, London, United Kingdom
4. Fundacão Oswaldo Cruz, Flavivirus Reference Laboratory, Rio de Janeiro, Rio de Janeiro, Brazil
5. Section Clinical Tropical Medicine, Department of Infectious Diseases, Heidelberg University Hospital, Heidelberg, Germany
6. Departamento de Medicina Interna, Universidade de Pernambuco, Recife, Pernambuco, Brazil
7. Institute of Medical Biometry and Informatics, University of Heidelberg, Heidelberg, Germany
8. Pediatric Infectious Diseases and Immunodeficiencies Unit, Hospital Universitari Vall d'Hebron, Vall d'Hebron Research Institute, Barcelona, Spain
9. Great Ormond Street Institute of Child Health, University College London, United Kingdom
10. Departamento de Medicina Tropical, Universidade Federal de Pernambuco, Recife, Pernambuco, Brazil
11. Departamento de Medicina Social, Universidade Federal de Pernambuco, Recife, Pernambuco, Brazil
12. Department of Infectious and Parasitic Diseases and Instituto de Medicina Tropical, Faculdade de Medicina da Universidade de Sao Paulo, Sao Paulo, Brazil
13. Facultad de Ciencias de la Salud, Universidad de Carabobo, Valencia, Venezuela
14. Mexican Institute of Social Security, Mexico City, Mexico
15. Evandro Chagas National Institute of Infectious Diseases, Fundação Oswaldo Cruz, Rio de Janeiro, Rio de Janeiro, Brazil
16. Department of Child and Adolescent Health, University of the West Indies, Mona Campus, Kingston, Jamaica
17. Instituto Aggeu Magalhães, Fundação Oswaldo Cruz, Recife, Pernambuco, Brazil
18. Instituto de Medicina Tropical Alexander von Humboldt, Universidad Peruana Cayetano Heredia, Lima, Peru
19. INSERM Centre d'Investigation Clinique 1424, Centre Hospitalier Universitaire de Pointe-à-Pitre, Guadeloupe, France

For peer review only - http://bmjopen.bmj.com/site/about/guidelines.xhtml

20. Service de Maladies Infectieuses et Tropicales, Dermatologie, Médecine Interne, Centre Hospitalier Universitaire de Pointe-à-Pitre, Guadeloupe, France
21. Université des Antilles et de la Guyane, Faculté de Médecine Hyacinthe Bastaraud, EA 4537, Pointe-à-Pitre, Guadeloupe, France.
22. Department of Viroscience, Erasmus Medical Center, Rotterdam, The Netherlands
23. Universidade Federal de Minas Gerais, Belo Horizonte, Minas Gerais, Brazil
24. Department of Infectious Diseases and Microbiology, University of Pittsburgh, Pittsburgh, Pennsylvania, United States of America
25. Universidad Industrial de Santander, Bucaramanga, Colombia
26. Instituto de Ciências Biológicas, Universidade de Pernambuco, Recife, Pernambuco, Brazil
27. Instituto Fernandes Figueira – Fundação Oswaldo Cruz, Rio de Janeiro, Rio de Janeiro, Brazil
28. Emerging Pathogens Institute, University of Florida, Gainesville, Florida, United States of America
29. Centro Nacional de Enfermedades Tropicales, Santa Cruz de la Sierra, Bolivia
30. Universidad Católica de Santiago de Guayaquil, Ecuador
31. SOSECALI C., Ltda, Guayaquil, Ecuador
32. University of Groningen, University Medical Center Groningen, Department of Medical Microbiology and Infection Prevention, The Netherlands
33. Instituto de Patologia Tropical e Saúde Publica, Universidade Federal de Goiás, Goiânia, Goiás, Brazil.
34. Dipartimento di Salute della Donna e del Bambino, Università degli Studi di Padova, Italy
35. Université *d'*Aix-Marseille, Marseille, France
36. Department of Epidemiology and Global Health, Umeå Universite, Umeå, Sweden

Corresponding author:

Claire Thorne, Population Policy and Practice Research and Teaching Department, University College London Great Ormond Street Institute of Child Health, 30 Guilford Street, London WC1N 1EH, United Kingdom

claire.thorne@ucl.ac.uk

For peer review only

For peer review only - http://bmjopen.bmj.com/site/about/guidelines.xhtml

ABSTRACT

Introduction

Zika virus (ZIKV) infection in pregnancy has been associated with microcephaly and severe neurological damage to the fetus. Our aim is to document the risks of adverse pregnancy and birth outcomes and the prevalence of laboratory markers of congenital infection in deliveries to women experiencing ZIKV infection during pregnancy, using data from European Commission-funded prospective cohort studies in 20 centres in 11 countries across Latin America and the Caribbean.

Methods and analysis

We will carry out a centre-by-centre analysis of the risks of adverse pregnancy and birth outcomes, comparing women with confirmed and suspected ZIKV infection in pregnancy to those with no evidence of infection in pregnancy. We will document the proportion of deliveries in which laboratory markers of congenital infection were present. Finally, we will investigate the associations of trimester of maternal infection in pregnancy, presence or absence of maternal symptoms of acute ZIKV infection, and previous flavivirus infections with adverse outcomes and with markers of congenital infection. Centre-specific estimates will be pooled using a two-stage approach.

Ethics and dissemination

Ethical approval was obtained at each centre. Findings will be presented at international conferences and published in peer-reviewed open access journals, and discussed with local public health officials and representatives of the national Ministries of Health, Pan American Health Organization, and World Health Organization involved with ZIKV prevention and control activities.

Keywords

Zika virus; pregnancy; vertical transmission; Congenital Zika Syndrome; microcephaly; meta-analysis

For peer review only

Strengths and limitations of this study

- This will be a pooled analysis of data from 3 international consortia conducting prospective cohort studies of outcomes following ZIKV infection in pregnancy in 20 centres in 11 countries.
- Standardised definitions of outcomes will provide clarity about the absolute risks of adverse outcomes, which have not been reported consistently in prospective studies so far.
- These studies include a control group of women with no evidence of ZIKV infection in pregnancy, allowing improved estimation of the proportion of adverse events attributable to ZIKV in pregnancy.
- Inferences will be limited by: difficulties in distinguishing between women who did and who did not experience a ZIKV infection in pregnancy, due to the high frequency of mild and asymptomatic infections and the low sensitivity and specificity of diagnostic tools; low diagnostic sensitivity of markers of congenital infection; and outcome data that are not missing at random.

For peer review only

For peer review only - http://bmjopen.bmj.com/site/about/guidelines.xhtml

INTRODUCTION

Following the emergence of Zika virus (ZIKV) in Asia and the Pacific in 2013 (1) and the subsequent introduction to Brazil (2), clusters of neonates with severe neurological complications and microcephaly were observed across Latin America. Following recent experiences with the H1N1 influenza pandemic and Ebola outbreak in Western Africa, the need for coordinated international research on ZIKV was quickly recognized. In January 2016, before the World Health Organization (WHO) declared a Public Health Emergency of International Concern (3), the European Commission (EC) issued a funding call to set up a network in Latin America and the Caribbean with the aim of implementing and coordinating urgently required research, while simultaneously contributing to research capacity and preparedness for other emerging infectious diseases. Three consortia were funded: ZIKAlliance (https://zikalliance.tghn.org/) (4,5), ZikaPLAN (6) (https://zikaplan.tghn.org/), and ZIKAction (http://zikaction.org/). All are multidisciplinary international collaborations with active investigations in epidemiology, virology, immunology, diagnostics, mathematical modelling, social science, and animal studies. Each consortium includes its own prospective cohort study of ZIKV in pregnancy and a shared work package that aims to ensure the harmonization of protocols and data sets in order to facilitate a pooled analysis of cohort data. The primary aim of the pooled analysis is to investigate the incidence of adverse outcomes of ZIKV infection in pregnancy, including "congenital infection, microcephaly, Zika congenital syndrome, and other sequelae of ZIKV infection".

The aim of this paper is to present a protocol for this pooled analysis. Data has been or is still being collected in multiple sites in 20 regional coordinating centres spread over 11 countries and regions across Latin America and the Caribbean. There are 15 ZIKAlliance centres: Sao Paulo, Rio de Janeiro, Recife and Belo Horizonte (Brazil); Valencia (Venezuela); Bucaramanga (Colombia); Guayaquil (Ecuador); Lima (Peru); Jalisco, Nayarit, Veracruz, Yucatan (Mexico); Santa Cruz de la Sierra (Bolivia); Havana (Cuba); Guadeloupe (French Territory of the Americas); 3 ZikaPLAN centres: Goiânia, Rio de Janeiro, Recife (Brazil); and 2 ZIKAction centres: Kingston (Jamaica) and Port-au-Prince (Haiti). Recruitment to ZIKAlliance began May 2017, December 2015 for ZikaPLAN , and September 2017 for ZIKAction. Over 700 women with confirmed infection had been recruited by April 2020.

Several studies of ZIKV in pregnancy have recently been published. In registry-based studies (7-9), fetuses and newborns of women with confirmed infection in pregnancy have been reported to have "potentially Zika-related" adverse outcomes at rates of up to 15%, with higher risk of Zika-associated adverse outcomes in the first trimester. Registry-based studies are likely to over-estimate the risk of severe clinical

For peer review only - http://bmjopen.bmj.com/site/about/guidelines.xhtml

manifestations and underestimate the risk of more mild clinical presentations because they recruit both prospectively ascertained ZIKV-infected pregnant women and women whose infection was recognized retrospectively following the birth of an infant with congenital abnormalities. Prospective studies of congenital infection have variously reported 25% "severe" and 21% "mild to moderate" outcomes in French Guiana (10), and 27% adverse outcomes in Brazil (11). The specificity of these outcome definitions for ZIKV in pregnancy is not known as these studies did not include a control group of women with no ZIKV infection in pregnancy. In another Brazilian study, the risk of adverse outcomes was reported to be 46% in births to women with NAAT (Nucleic Acid Amplification Test)-confirmed ZIKV infection in pregnancy compared to 11.5% in NAAT-negative women (12). In a large prospective study based in the French Territories of the Americas, among infants born to women with NAAT-confirmed ZIKV infection, 7.0% presented with neurologic or ocular birth defects and 3.1% met the study's criteria for Congenital Zika Syndrome (CZS) (13), which is characterized by several unique features (14). An important limitation in the comparison of the results of these different studies is the lack of a standard definition of CZS and of the clinical and diagnostic procedures used to evaluate these children, leading to possible misclassification of the outcomes studied.

The vertical transmission rate is the probability of congenital infection in births to women with infection in pregnancy. The rates reported so far, 26% (11) and 35% (10), are based on laboratory markers of congenital infection such as NAAT or IgM in the fetus or newborn. However, a prospective cohort retrospectively reconstructed from a register study estimated the vertical transmission rate to be only 9% (15). Comparison of these rates is difficult as different markers and different biological samples were used. In addition, although these tests (NAAT and IgM) are analytically sensitive and specific they have poor diagnostic sensitivity as markers of congenital infection. These markers were absent from serum in a high proportion of CZS cases (16) (16,17) and in newborns with other potentially ZIKV-related adverse outcomes born to women with confirmed ZIKV during pregnancy (10,11). Clearance of virus from amniotic fluid and fetal blood has been reported in cases of CZS, even when ZIKV is found in brain tissue post-mortem (18,19). It therefore appears that fetal infection may occur, causing profound damage, but clearing before delivery and leaving no discernable immunological trace in serum. Consequently, in this study we will document the prevalence of markers of congenital infection using uniform criteria, recognizing that this is an underestimate of the true vertical transmission rate.

Regarding effect modifiers, a number of studies have reported a higher incidence of congenital abnormalities following maternal infections in the first trimester (7,10–11,14). Maternal symptoms during

For peer review only - http://bmjopen.bmj.com/site/about/guidelines.xhtml

acute ZIKV infection do not appear to be a risk factor for adverse outcomes (20). There is evidence of antibody dependent enhancement of ZIKV by dengue virus (DENV) antibody in animal models (21), but it is unclear whether previous DENV infection or exposure to other flaviviruses has a protective, risk-enhancing, or null effect, in maternal or congenital infection in humans (22). It also remains to be established whether a previous ZIKV infection confers protective immunity. Little is currently known about risk factors for trans-placental transmission of ZIKV.

The analysis plan described here complements the recently published protocol of the Zika Virus Individual Participant Data (IPD) Consortium (23), which will eventually include data from the three EC consortia as well as data from many other sources. Although the objectives of the protocols are similar, different methods are proposed in relation to design of included studies, definition of congenital infection, and approach to imperfect diagnosis of maternal infection.

In light of the unexplained heterogeneity in reported rates of adverse outcomes, and the variation in prevalence of markers of congenital infection, a pooled analysis of data from 20 centers following similar protocols with harmonized definitions of clinical and laboratory outcomes will provide important new information on outcomes of ZIKV in pregnancy.

OBJECTIVES OF THE JOINT ANALYSIS

1. To estimate the risk of adverse outcomes in the fetus, newborn, and child following maternal ZIKV infection in pregnancy, compared to outcomes in controls with no evidence of maternal infection in pregnancy.
2. To estimate the prevalence of markers of congenital infection among fetuses and liveborn infants following maternal ZIKV infection during pregnancy.
3. To assess the associations between trimester of maternal infection, presence or absence of maternal symptoms, and previous flavivirus infections with adverse outcomes and markers of congenital infection.

METHODS

Participants

For peer review only - http://bmjopen.bmj.com/site/about/guidelines.xhtml

Pregnant women were eligible only if their infection status during pregnancy (infected or not infected) was ascertained prior to the detection of adverse outcomes, or was not influenced by fetal examination or outcome on delivery. This definition is compatible with retrospective testing of previously collected maternal samples, after delivery. Although the unit of recruitment is the mother, the unit of analysis is the fetus, newborn, and infant; multiple births are sufficiently rare to be treated as independent observations (24).

Study design

Consenting women were screened in pregnancy for markers of ZIKV infection. Those in whom maternal infection in pregnancy (MIP) was suspected were followed with enhanced investigations. In ZIKAction and ZIKAlliance, all deliveries to these women, including fetal losses, stillbirths, and newborns were examined clinically and tested for markers of congenital infection. This testing was not routinely performed in ZikaPLAN. In all three cohorts, newborns were prospectively followed to identify any adverse outcomes that may develop later. In all three consortia, a sample of newborns delivered to women with no evidence of infection in pregnancy served as an unexposed control group.

There were some differences between the protocols adopted by the three consortia in terms of how women were recruited into the study, and the choice and scheduling of tests and investigations (Supplementary Table S1). In ZIKAction and ZIKAlliance, women were recruited regardless of symptoms during pregnancy, although report of symptoms was recorded. In ZikaPLAN, only women with rash, a common sign of ZIKV infection, were recruited. Statistical analyses will therefore be stratified by whether the mother reported symptoms in pregnancy. .

When the studies were designed, there was little information on the risk of adverse outcomes of ZIKV in pregnancy, on vertical transmission rates, nor on what infection rates among pregnant women might be expected. Formal sample size calculations were not undertaken.

Patient and public involvement statement: There was no patient or public involvement in this study

Target parameters and terminology of vertical transmission studies

Six categories of joint congenital infection status and maternal infection status (A-F) are defined in Table 1, which illustrates the logic of an idealised prospective study. The usual target parameters are the *vertical transmission rate*, which is the probability of congenital infection following MIP, (A+B) / (A+B+C+D); and

For peer review only - http://bmjopen.bmj.com/site/about/guidelines.xhtml

the *rate of adverse outcomes in those with congenital infection,* A/(A+B). The definitions of "adverse outcomes", congenital infection, and MIP will be determined by a Joint Diagnostics Group and a Joint Endpoint Review Group, after the data have been assembled. Estimates of these parameters are standard in the classic studies of vertical transmission of human immune-deficiency virus (HIV) (25,26), toxoplasmosis (27–29) and cytomegalovirus (CMV) (30). In studies of less specific outcomes, the event rate C/(C+D) in fetuses and newborns of women with MIP but in whom no congenital infection occurred (Paediatric Control Group 1 in Table 1) forms a comparison group (31,32) representing the adverse event rate that is due to MIP in the absence of congenital infection. The present analysis plan is modelled closely on these earlier studies, but includes adaptations to take account of the difficulties in diagnosing maternal and congenital ZIKV infection.

For example, because cases of congenital infection cannot be reliably identified by diagnostic tests, we can only estimate the prevalence of laboratory *markers of vertical infection* (i.e. NAAT or IgM) (Objective 2). Similarly, the "overall" (unconditional) adverse event rate is taken as the primary outcome for Objective 1; this includes all births to women with MIP, (A+C)/(A+B+C+D) (Table 1). All three consortia included a further control group of births to unexposed women, those with no infection in pregnancy (Maternal Control Group in Table 1). The adverse event rates in this group, E/(E+F), represents a baseline for comparison with the overall event rates in exposed women (33).

Although estimates of the vertical transmission rate are compromised, it may still be of interest to compare adverse outcome rates in the MIP with congenital infection group (i.e., A/(A+B)), the MIP without congenital infection group (i.e., C/(C+D)), and the No MIP group (i.e., E/(E+F)), as this may provide insight into whether adverse fetal outcomes are associated with MIP in the absence of demonstrable fetal infection.

In addition to the challenges associated with the laboratory definition of congenital infection, it is also difficult to discriminate between pregnancies with MIP and with No MIP as required in analyses based on Table 1. A positive NAAT result or seroconversion during pregnancy are sufficient to confirm MIP. However, even if tested *per protocol,* MIP may be missed due to the narrow window of detection of NAAT tests, perhaps as low as 14 days (34). Tests of recent infection, including IgM, IgG3 or avidity assays, may reflect infection during pregnancy, but may also be the result of infection prior to pregnancy (35), and immunologic cross-reactivity to DENV antibody may need to be ruled out (36). These tests therefore indicate suspected but not confirmed MIP. An IgG negative response in the woman or newborn at or tests, perhaps as low as 14 days (34). An IgG negative response in the woman or newborn at or shortly after

For peer review only - http://bmjopen.bmj.com/site/about/guidelines.xhtml

delivery can be taken as suggestive of No MIP, although the dynamics of ZIKV IgG are not well documented. While a negative IgG is likely to be uncommon in some sites, as some women will have experienced a ZIKV infection prior to pregnancy, it lowers the probability that a ZIKV infection would have occurred in pregnancy. Taking all this together, MIP status will be characterised as “Confirmed”, “Suspected, “No Evidence of MIP" (i.e., all NAAT and IgM tests negative), and "No MIP" (i.e., all NAAT and IgM tests negative AND IgG negative at or shortly after delivery). To maintain the principle of prospective ascertainment, confirmation of congenital infection will have no impact on the mother’s assigned infection status.

Definitions of variables

To support the pooled analyses, a Joint Diagnostics Group consisting of immunologists and virologists and a Joint Endpoint Review Group consisting of paediatricians with experience of congenital ZIKV will be convened to agree on standardized case definitions. The names and qualifications of members of both groups will be published at the time of the joint data analysis, together with rationale and process for their recruitment. Supplementary Table S2 provides some provisional definitions for: MIP (Confirmed, Suspected, No Evidence of MIP, No MIP), laboratory markers of congenital infection (present or absent), signs and symptoms compatible with Congenital Zika Syndrome (CZS), other potentially-Zika-related outcomes (OPZRO), and trimester of MIP. The Diagnostics Group will also have responsibility for definitions of covariates, such as previous flavivirus infection. Definitions of both diagnostic categories and clinical endpoints will be based on the best information available at the time of analysis, and will to the greatest extent possible, be harmonized across consortia and across centres within consortia. As the expert groups will be assembled from representatives of each consortium, they will already have examined the data prior to a joint analysis, so that blinding will not be possible; however, they will devise diagnostic and clinical criteria that can be applied objectively across the three consortia.

An essential aspect of the definitions for MIP status used in the statistical analyses below is that they must be based exclusively on the *prospective* diagnostic testing. For example, although CZS and/or laboratory evidence of congenital infection in the newborn provides compelling evidence of MIP, this would not affect the MIP status as ascertained prospectively. Thus, we expect to observe some newborns with CZS and/or with laboratory markers of congenital infection delivered to women with Suspected MIP, or even those categorized as having No Evidence of MIP.

For peer review only - http://bmjopen.bmj.com/site/about/guidelines.xhtml

STATISTICAL ANALYSIS

Analysis of data from individual centres

Descriptive tables

We will produce descriptive tables providing a breakdown of the numbers in each centre with Confirmed MIP, Suspected MIP, No Evidence of MIP, and No MIP, and the type of evidence on which this is based (e.g., NAAT, seroconversion, tests of recent infection) (Supplementary Table S3). The similar Supplementary Table S4 will document numbers with signs or symptoms compatible with CZS and with markers of congenital infection, and the type of evidence on which this is based, for example NAAT, IgM, or clinical markers (CZS).

Objective 1: Adverse Outcomes

Prospectively ascertained MIP status will be tabulated against overall (i.e., unconditional on congenital infection status) adverse outcomes (Table 2). Various risks can be estimated within each MIP category, including: the risk of signs and symptoms compatible with CZS, the risk of OPZRO, the risk of both combined, of individual signs and symptoms, or of signs and symptoms grouped in clinically (e.g., ophthalmologic defects) or embryologically meaningful ways. Outcomes may be binary (e.g., microcephaly) or continuous (e.g., head circumference), or multi-category (CZS-related outcomes, OPZRO, asymptomatic). As well as congenital anomalies, rates of standard outcomes, in the absence of congenital anomalies, will be documented, including: fetal loss, stillbirth, low birthweight, intra-uterine growth retardation (IUGR), and premature delivery.

The rate of adverse outcomes in the No MIP group represents the study-specific background rate of adverse outcomes (i.e., in the absence of MIP, Control Group 2), to be compared to rates in women with Confirmed MIP. Absolute risks will be estimated as well as risk ratios and risk differences.

Parallel sets of estimates will be calculated in the Suspected MIP and No Evidence of MIP groups, as particular adverse events indicate lack of diagnostic specificity and sensitivity in the testing protocol during pregnancy and are therefore informative regarding the effectiveness of the maternal testing

For peer review only - http://bmjopen.bmj.com/site/about/guidelines.xhtml

protocol in each centre; for example, microcephaly in the No Evidence of MIP group would indicate a lack of sensitivity. These sets of estimates may be pooled, respectively, with adverse outcome risks in Confirmed MIP and No MIP groups in sensitivity analyses.

Objective 2: Laboratory and clinical markers of vertical transmission

Estimates of the prevalence of markers of congenital infection will be produced in all centres with available data for each MIP group except the No MIP group, as the definition of No MIP is not compatible with laboratory markers of congenital infection. For this purpose, the numerator will be the number with markers of congenital infection, and the denominator will be the sum of the numbers with and without markers of congenital infection. Separate estimates will be obtained for: laboratory markers, clinical markers (namely CZS) , and combined laboratory and clinical markers (Table 3). Rates in the No Evidence of MIP group are of interest as they carry information about the diagnostic accuracy of the maternal testing protocol.

Objective 3: Effect of covariates

Separate estimates of the prevalence of adverse pregnancy outcomes, and the probability of laboratory and clinical markers of congenital infection, will be produced for each trimester of maternal infection, and by presence or absence of maternal symptoms.

Analyses of adverse event frequencies and of markers of congenital infection can be extended to include multiple covariates, using logistic regression. These might include potential *effect modifiers,* such as previous arbovirus infection or co-infection, or *confounding factors* such as socio-economic indicators likely to be associated with both arbovirus exposure and adverse outcomes. However, at the time of writing it is not known whether sufficient data will be available for regression analyses.

Secondary objectives

ZIKV infection in pregnancy could lead to adverse pregnancy and birth outcomes either following a congenital infection (cell A in Table 1) or in the absence of congenital infection (equally in cells A and C). Although absence of markers of congenital infection does not rule out congenital infection, we might still

For peer review only - http://bmjopen.bmj.com/site/about/guidelines.xhtml

expect to observe more adverse outcomes in fetuses and newborns with laboratory markers of congenital infection if those outcomes are caused by congenital infection. By contrast, adverse outcomes that are the result of MIP in the absence of congenital infection (Cell E in Table 1) should occur equally with or without laboratory markers of congenital infection. Based on literature on other infections in pregnancy, including dengue virus (37), adverse outcomes associated with MIP in the absence of congenital infection potentially include: fetal loss, stillbirth, prematurity, IUGR, and low birthweight for gestational age (38). The analysis would be based on a tabulation of presence or absence of neonatal symptoms, or sets of symptoms (Supplementary Table S5), and would be stratified by trimester of maternal infection, as this is likely to be associated with the presence of markers of congenital infection and with adverse outcomes.

Missing Covariates

All analyses will be conducted on a "complete case" basis, in the first instance. Methods for handling missing covariates, such as imputation (39), will be considered after the extent and patterns of missing data have been explored.

Combining data across centres

The above analyses will generate a series of centre-specific estimates of proportions, relative risks comparing MIP and No MIP groups, risk-differences, and means of continuous variables, stratified by trimester and maternal clinical presentation. If logistic regression is used to examine effect-modifiers and confounders, further estimates of interaction terms or adjusted estimates can also be produced.

All these estimates can be combined across centres using fixed or random effects models in a "two-stage" meta-analysis. Bayesian Markov chain Monte Carlo methods will be used as this will facilitate the use of exact binomial and multinomial likelihoods, which have a better performance with low and zero cells counts. Vague priors will be employed. Centre-specific random effect estimates will be sampled from beta distributions for binomial outcomes data, Dirichlet distributions for multinomial data, and normal distributions for continuous data. We will report ranges, between-centre standard deviations, mean effects and predictive effects with 95% credible intervals for each estimate.

For peer review only - http://bmjopen.bmj.com/site/about/guidelines.xhtml

For peer review only

In combining estimates from different centres, we will take account of the fact that in ZikaPLAN only women with rash were recruited, so that women with no ZIKV infection may have experienced other exanthematic infections (12), including arbovirus infections such as dengue and chikungunya, which may themselves be associated with adverse outcomes (40,41).

Depending on the results of two-stage analyses, and the completeness of covariate data, an individual patient data one-stage meta-analyses will be considered for each objective, as a secondary or sensitivity analysis, with centre as an additional fixed “intercept” term.

For peer review only

Sensitivity analyses

Definitions of MIP status, ZIKV-related outcomes, and laboratory markers of congenital infection

We will report differences in adverse event rates between Confirmed MIP and Suspected MIP, and between No Evidence of MIP and No MIP. If the differences are small, we will produce results pooling these categories as a sensitivity analysis.

Further, the No Evidence of MIP category can be subdivided into women who were tested *per protocol* and those who may have been tested less completely. The impact of compliance with protocols will be explored, as it is expected to impact on the proportion of women with MIP who are classified as “No evidence of MIP” and, hence, on the probability of observing adverse outcomes in this group. Similarly, we will conduct sensitivity analyses around the definitions of Confirmed and Suspected MIP on advice from the Joint Diagnostics Group.

Alternative sets of estimates will be generated using alternative criteria for CZS-related outcomes and OPZRO, that are more, or less, specific for ZIKV in pregnancy. Similarly, we will explore the impact of varying the laboratory criteria for congenital infection on the advice of the Joint Diagnostics Group.

Independent ascertainment of outcomes

A critical requirement of all these analyses is that the ascertainment of markers of congenital infection status and clinical outcomes in the fetuses and newborns, and developing infant are all independent of

For peer review only - http://bmjopen.bmj.com/site/about/guidelines.xhtml

each other, and also independent of MIP status. For example, ideally the same laboratory testing for congenital infection is carried out regardless of whether the pregnancy outcome is a fetal loss, termination of pregnancy, stillbirth, a case of CZS, or an apparently healthy asymptomatic infant.

These assumptions are difficult, and in certain respects (e.g., first trimester fetal loss and terminations) not possible, to fully implement in practice. To address these inevitable limitations in the analysis, which are expected to impact more on objective 2 than on objective 1, we will carry out exploratory analyses aimed at detecting potential deviations from protocol. For example, the distribution of trimester of MIP should not be associated with prospectively ascertained MIP status. Guided by the results, we will carry out sensitivity analyses that make a range of assumptions about the distribution of missing data, especially data on markers of congenital infection. A series of scenarios will be examined to assess robustness of results to inherent and/or unplanned deviations from the ideal protocols required for unbiased estimation of the target parameters.

DISCUSSION

Risks of CZS and other adverse birth outcomes of ZIKV infection in pregnancy can only be assessed through studies that recruit women whose infection status is prospectively ascertained, or, if retrospective, ascertainment is independent of outcomes. However, reported risks of adverse outcomes even from prospective studies have been highly variable (10–13), as have vertical transmission rates based on laboratory markers of congenital ZIKV infection (10,11,15). An important role for joint analyses of multiple studies is to explore whether this heterogeneity in outcomes can be explained by individual or study-level covariates. To do this, it is essential that incidental sources of variation, such as those arising from differences in outcome reporting or diagnostic testing, are controlled or eliminated as much as possible. One of the most difficult sources of variation between consortia, and between sites within consortia, lies in diagnosis of maternal infection. Our approach is to have an Expert Diagnostics Group produce a harmonised classification of Confirmed MIP, Suspected MIP, No Evidence of MIP, and No MIP, and to compute a range of estimates of the relative effect of maternal infection on outcomes, grouping these in different ways. An analysis based on the Confirmed MIP and No MIP groups alone would be expected to generate the largest estimates of relative effect, because both poor sensitivity and poor specificity will tend to bias effect estimates towards the null.

An alternative proposal (23) in relation to maternal infection status is to treat test sensitivity and specificity as study-level covariates in a meta-regression, but the risk of false positive diagnosis depends

For peer review only - http://bmjopen.bmj.com/site/about/guidelines.xhtml

more on the incidence of ZIKV and cross-reacting antibodies to other arbovirus infections such as dengue than on test specificity. The Zika Virus IPD Consortium protocol differs in two other ways. First it proposes to include surveillance studies, which may result in over-estimating the risk of adverse outcomes due to retrospective ascertainment of infected women following adverse newborn outcomes (7,42). Second, congenital infection is to be defined by clinical and radiological criteria alone.

Other statistical methods may have been developed by the time the data becomes available for these analyses. Whatever form of analysis is adopted, a standardised pooled analysis from three large consortia comprising 20 centres will provide valuable information about these parameters, which will assist in framing a public health response and advice to women who might be exposed in future. It may also throw light on pathologic mechanisms leading to adverse outcomes, which could help in the development of therapeutic or prophylactic interventions.

Acknowledgements The authors express their thanks to Tom Byrne for organising the references.

Collaborators Other members of the EC Zika Consortia Vertical Transmission Study Group are: Joshua ANZINGER (Department of Microbiology, Virology Section, University of the West Indies, Mona Campus, Kingston, Jamaica; Global Virus Network, Baltimore, MD, USA), Heather BAILEY (Institute for Global Health, University College London, UK) Valery E M BEAU DE ROCHARS (Emerging Pathogens Institute, University of Florida, Gainesville, Florida, United States of America), Luana DAMASCENO (Evandro Chagas National Institute of Infectious Diseases, Fundação Oswaldo Cruz, Rio de Janeiro, Brazil), Maria de Fatima Pessoa Militão DE ALBUQUERQUE (Instituto Aggeu Magalhães, Fundação Oswaldo Cruz, Recife, Pernambuco, Brazil), Rafael F. O. FRANÇA (Instituto Aggeu Magalhães, Fundação Oswaldo Cruz, Recife, Pernambuco, Brazil), Hugo Lopez GATELL RAMIREZ (Centro de Investigación sobre Enfermedades Infecciosas, Instituto Nacional de Salud Pública, Cuernavaca, Mexico), Adriana GOMEZ (Universidad Industrial de Santander, Bucaramanga, Colombia), Fabiana GORDON (Population Health Sciences, Bristol Medical School, University of Bristol, Bristol, United Kingdom), Maria G GUZMAN (Instituto de Medicina Tropical Pedro Kourí, La Havana, Cuba), Daniel LANG (Fondazione Penta Onlus, São Paulo, São Paulo, Brazil), Rigan LOUIS (Emerging Pathogens Institute, University of Florida, Gainesville, Florida, United States of America), Anyela Lozano (Universidad Industrial de Santander, Bucaramanga, Colombia), Eric MARTINEZ (Instituto de Medicina Tropical Pedro Kourí, La Havana, Cuba), Philippe MAYAUD (Department of Clinical Research, London School of Hygiene and Tropical Medicine, London, UK), Ricard ORTIZ SERRANO (Universidad Autónoma de Bucaramanga, Bucaramanga, Colombia), Silvia P. SALGADO (Instituto Nacional de Salud Pública e Investigación, Guyaquil, Ecuador), Aluisio Augusto Cotrim

For peer review only - http://bmjopen.bmj.com/site/about/guidelines.xhtml

SEGURADO (Department of Infectious and Parasitic Diseases and Instituto de Medicina Tropical, Faculdade de Medicina da Universidade de Sao Paulo, Sao Paulo, Brazil), Zilton F. M. VASCONCELOS (Instituto Fernandes Figueira – Fundação Oswaldo Cruz, Rio de Janeiro, Rio de Janeiro, Brazil), Isabelle Freire Tabosa VIANA (Instituto Aggeu Magalhães, Fundação Oswaldo Cruz, Recife, Pernambuco, Brazil), Luis Angel VILLAR (Universidad Industrial de Santander, Bucaramanga, Colombia)

Contributors A steering group (CT, TJ, KDR, RAAX, AEA, EBB, NA) was formed to prepare a Joint Statistical Analysis Plan pursuant to the terms of the three EC Zika Consortia funding, at the direction of the consortia PIs (CG, XdL and AW-S). After initial discussions the steering group delegated the task to AEA, EBB and NA. The SAP was conceptualised and drafted by AEA with EBB and NA. After several iterations, the draft was revised based on comments from CT, DB, ASA, TJ, KDR, MP, DBM, RAAX, MCM, AT, CG, XdL and AW-S. Comments were then solicited from TVBA, VIAS, SEBC, VHBA, PB, CDCC, WVS, JEGH, BH, MK, CMTM, MMT, ETAM, URM, MEM, JGM, BR, PMSV, CSS and MDT and a revision was drafted by AEA, EBB and NA. The final draft was agreed by all authors.

For peer review only

Funding European Union Directorate-General for Research grant numbers 734548 (ZIKAlliance), 734584 (ZikaPLAN), and 734857 (ZIKAction) Horizon 2020 programme of the European Commission. EBB was also supported by Wellcome Trust & the UK's Department for International Development (205377/Z/16/Z to LCR; https://wellcome.ac.uk/).

Patient consent for publication Not required

Competing interests All authors have completed the ICMJE uniform disclosure form. The following authors report grants from the European Commission (AET, EB, NA, DB, TJ, KDR, CT, CDC, ASA, JGM, CG, XL, AWS); EBB reports funding from by Wellcome Trust & the UK's Department for International Development; MMT reports grants from Conselho Nacional de Desenvolvimento Cientifico e Tecnologico, Brazil and from Fundacao de Amparo a Pesquisa do EStado de MInas Gerais (FAPEMIG, Brazil), during the conduct of the study; MK has a patent on zika diagnostics pending.

Ethics and dissemination

The following ethical committees / institutional review boards have approved the participating studies:

For peer review only - http://bmjopen.bmj.com/site/about/guidelines.xhtml

Bolivia: El Tribunal de Etica del Colegio Médico de Santa Cruz -Tribunal Departamental de Etica y Deontología Médica (TEDM) (018/2017; 019/2017).

Brazil: Comissão Nacional de Ética em Pesquisa (CONEP) (2.493.696/CAAE 67410117.3.1001.5262); Comitê de Ética em Pesquisa do Hospital Sofia Feldman (CEP/HSF) (2.549.675/CAAE 67410117.3.3001.5132); Comitê de Ética em Pesquisa da UFMG (COEPE-UFMG), Belo Horizonte (2.877.160/CAAE 67410117.3.2002.5149); Comitê de Ética em Pesquisa do Instituto Nacional de Infectologia Evandro Chagas (INI), Manguinhos - Rio de Janeiro; Comitê de Ética em Pesquisa da Faculdade de Medicina da Universidade de São Paulo (CEP-FMUSP) (2.195.948 /CAAE 63567917.6.0000.0065; 2.262.420/CAAE 74853517.0.0000.0065; 1.938.335/CAAE 54699416.0.0000.0065); Comitê de Ética em Pesquisa (CEP) do Instituto Aggeu Magalhães, Recife (2.777.436/CAAE 80243817.5.0000.5190; 2.737.404/CAAE 67410117.3.2001.5190); CEP Universidade Federal de Goiás, Goiânia (64534017.7.0000.5083); CEP Instituto Fernandes Figueira, Rio de Janeiro (52675616.0.0000.5269); CEP Instituto Aggeu Magalhães, Recife, Brazil (53240816.4.0000.5190)

Colombia: Comité de Ética en Investigación Científica de la Universidad Industrial de Santander (CEINCI-UIS) (4110)

Cuba: Comité de Ética de la Investigación del Instituto de Medicina Tropical “Pedro Kourí” (25–18; 59–16; 40–16)

Ecuador: Comité de Ética del Hospital Clínica Kennedy, Guayaquil (HCK-CEISH-17-0026)

Haiti: Ministère de la Santé Publique et de la Population, Comité National de Bioéthique (1718-7)

Jamaica: University of the West Indies (Mona Campus) Research Ethics Committee (104-16/17)

Mexico: Comité de Ética en Investigación y Comité de Investigación de la Comisión Nacional de Investigación Científica del IMSS (R-2016-785-076; R-2018-785-42); Comité de Ética en Investigación del Instituto Nacional de Salud Pública and Comité de Bioseguridad del Instituto Nacional de Salud Pública (17CEI00420160708/13CEI1700736; 18CI17007029/CI-620-2018; CB18–133; CI-369-2017; CB17–143).

Peru: Comité Institucional de Ética en Investigación de la Universidad Peruana Cayetano Heredia (101582/766–22-17; 101582/R-234-25-18)

UK: University College London Research Ethics Committee (3715/001)

US: Institutional Review Board, University of Florida (IRB201601229)

Venezuela: Comisión de Bioética y Bioseguridad, Universidad de Carabobo (CPBB-UC) (CPBBUC-020-2017-DIC-AS; CPBBUC-008-2018-DIC-SB.)

For peer review only - http://bmjopen.bmj.com/site/about/guidelines.xhtml

Findings will be presented at international conferences and published in peer-reviewed open access journals, and discussed with local public health officials and representatives of the national Ministries of Health, Pan American Health Organization, and World Health Organization involved with ZIKV prevention and control activities.

For peer review only

For peer review only - http://bmjopen.bmj.com/site/about/guidelines.xhtml

TABLES

Table 1. Design of an idealized prospective vertical transmission study. The vertical transmission rate is estimated by (A+B)/(A+B+C+D). The rate of adverse outcomes conditional on congenital infection is A/(A+B). This can be compared with the rate of adverse outcomes in newborns with no congenital infection, C/(C+D), who form a control group (Pediatric Control Group) to account for potential confounders associated with maternal infection.

An overall, non-conditional estimate of the adverse event rate is (A+C)/(A+B+C+D).

Follow-up of births to women with no MIP creates a second control group (Maternal Control Group), in which the rate of adverse outcomes, E/(E+F), can be compared to the rate in births with no congenital infection to women with MIP. Estimated effects of MIP based on the Maternal Control Group are vulnerable to confounding by factors associated with MIP. Some cells are set to zero as there can be no congenital infection without MIP.

			**Maternal Infection Status**	
			MIP	No MIP
**Congenital Infection Status**	**congenital infection**	Adverse outcomes	A	0
		No Adverse outcomes	B	0
	**No congenital infection**	Adverse outcomes	C	E
		No Adverse outcomes	D	F

For peer review only

Table 2. Scheme for a generic analysis of risks of adverse outcomes by prospectively ascertained Maternal Infection in Pregnancy (MIP) status. Presence or absence of fetal and neonatal signs or symptoms (e.g., microcephaly, brain calcifications, arthrogryposis) and other Potentially Zika-related outcomes (e.g., fetal loss) in the different MIP groups will be compared.

**CENTRE**	**Maternal Infection in Pregnancy status**			
	Confirmed	Suspected	No Evidence of MIP	No MIP
**Symptom 1**				
**Symptom 2**				
**Symptom 3**				
**:**				
**No Symptoms**				
**TOTAL**				

Table 3. Scheme for generic analysis of markers of congenital infection by prospectively ascertained Maternal Infection in Pregnancy (MIP) status. Markers of congenital infection may include laboratory markers, clinical markers, such as microcephaly, or both combined.

**CENTRE**	**Maternal Infection in Pregnancy status**			
	Confirmed	Suspected	No Evidence of MIP	No MIP
**Both laboratory and clinical markers of congenital infection**				
**Only laboratory markers of congenital infection**				
**Only clinical markers of congenital infection**				

For peer review only - http://bmjopen.bmj.com/site/about/guidelines.xhtml

**No markers of congenital infection**				
**Not tested**				
**TOTAL**				

For peer review only

For peer review only - http://bmjopen.bmj.com/site/about/guidelines.xhtml

References

1. Roth A, Mercier A, Lepers C, Hoy D, Duituturaga S, Benyon E, et al. Concurrent outbreaks of dengue, chikungunya and zika virus infections null An unprecedented epidemic wave of mosquito-borne viruses in the Pacific 2012null2014. Eurosurveillance. 2014;19(41):1–8.
2. Campos GS, Bandeira AC, Sardi SI. Zika Virus Outbreak, Bahia, Brazil. Emerg Infect Dis. 2015;21(10):1885–6.
3. Gulland A. Zika virus is a global public health emergency, declares WHO. BMJ. 2016;352(February):i657.
4. Avelino-Silva VI, Mayaud P, Tami A, Miranda MC, Rosenberger KD, Alexander N, et al. Study protocol for the multicentre cohorts of Zika virus infection in pregnant women, infants, and acute clinical cases in Latin America and the Caribbean: The ZIKAlliance consortium. BMC Infect Dis. 2019;19(1):1–14.
5. De Alencar Ximenes RA, De Barros Miranda-Filho D, Brickley EB, Montarroyos UR, Martelli CMT, de Araújo TVB, et al. Zika virus infection in pregnancy: Establishing a case definition for clinical research on pregnant women with rash in an active transmission setting. PLoS Negl Trop Dis. 2019;13(10):1–17.
6. Wilder-Smith A, Preet R, Renhorn KE, Ximenes RA, Rodrigues LC, Solomon T, et al. ZikaPLAN: Zika Preparedness Latin American Network. Glob Health Action. 2017;10(1):1398485.
7. Reynolds M, Jones A, Petersen E, Lee E, Rice M, Bingham A. Vital Signs: Update on Zika Virus–Associated Birth Defects and Evaluation of All U.S. Infants with Congenital Zika Virus Exposure — U.S. Zika Pregnancy Registry, 2016. Morb Mortal Wkly Rep. 2017;66(13):366–73.
8. Shapiro-Mendoza CK, Rice ME, Galang RR, Fulton AC, VanMaldeghem K, Prado MV, et al. Pregnancy Outcomes After Maternal Zika Virus Infection During Pregnancy — U.S. Territories, January 1, 2016–April 25, 2017. MMWR Morb Mortal Wkly Rep. 2017;66(23):615–21.
9. Honein MA, Dawson AL, Petersen EE, Jones AM, Lee EH, Yazdy MM, et al. Birth defects among fetuses and infants of US women with evidence of possible zika virus infection during pregnancy. JAMA - J Am Med Assoc. 2017;317(1):59–68.
10. Pomar L, Vouga M, Lambert V, Pomar C, Hcini N, Jolivet A, et al. Maternal-fetal transmission and adverse perinatal outcomes in pregnant women infected with Zika virus: prospective cohort study in French Guiana. Bmj. 2018;k4431.
11. Nogueira ML, Nery Júnior NRR, Estofolete CF, Bernardes Terzian AC, Guimarães GF, Zini N, et al. Adverse birth outcomes associated with Zika virus exposure during pregnancy in São José do Rio Preto, Brazil. Clin Microbiol Infect. 2018 Jun;24(6):646–52.
12. Brasil P, Pereira J, Moreira E, Riberio Nogueira RM, Damasceno L, Wakimoto M, et al. Zika Virus Infection in Pregnant Women in Rio de Janeiro. N Engl J Med. 2016;56(11):1024–5.
13. Hoen B, Schaub B, Funk AL, Ardillon V, Boullard M, Cabié A, et al. Pregnancy Outcomes after ZIKV Infection in French Territories in the Americas. N Engl J Med. 2018;378(11):985–94.
14. Moore CA, Staples JE, Dobyns WB, Pessoa A, Ventura C V., Da Fonseca EB, et al. Characterizing the pattern of anomalies in congenital zika syndrome for pediatric clinicians. JAMA Pediatr. 2017;171(3):288–95.
15. Conners EE, Lee EH, Thompson CN, Mcgibbon E, Rakeman JL, Iwamoto M, et al. Zika Virus Infection Among PregnantWomen and Their Neonates in NewYork City, January 2016–June 2017. Obs Gynecol. 2018;132:487–95.
16. van der Linden V, Pessoa A, Dobyns W, Barkovich AJ, Júnior H van der L, Filho ELR, et al. Description of 13 Infants Born During October 2015–January 2016 With Congenital Zika

For peer review only - http://bmjopen.bmj.com/site/about/guidelines.xhtml

Virus Infection Without Microcephaly at Birth — Brazil. Morb Mortal Wkly Rep. 2016;65(47):1343–8.
17. de Araújo TVB, Ximenes RA de A, Miranda-Filho D de B, Souza WV, Montarroyos UR, de Melo APL, et al. Association between microcephaly, Zika virus infection, and other risk factors in Brazil: final report of a case-control study. Lancet Infect Dis. 2018 Mar;18(3):328–36.
18. Schaub B, Vouga M, Najioullah F, Gueneret M, Monthieux A, Harte C, et al. Analysis of blood from Zika virus-infected fetuses: a prospective case series. Lancet Infect Dis. 2017;17(5):520–7.
19. Rodó C, Suy A, Sulleiro E, Soriano-Arandes A, Antón A, García-Ruiz I, et al. In utero negativization of Zika virus in a foetus with serious central nervous system abnormalities. Clin Microbiol Infect. 2018;24(5):549.e1-549.e3.
20. Paixao ES, Leong WY, Rodrigues LC, Wilder-Smith A. Asymptomatic prenatal Zika virus infection and congenital Zika syndrome. Open Forum Infect Dis. 2018;5(4):1–5.
21. Bardina S V., Bunduc P, Tripathi S, Duehr J, Frere JJ, Brown JA, et al. Enhancement of Zika virus pathogenesis by preexisting antiflavivirus immunity. Science (80-). 2017;356(6334):175–80.
22. Masel J, McCracken MK, Gleeson T, Morrison B, Rutherford G, Imrie A, et al. Does prior dengue virus exposure worsen clinical outcomes of Zika virus infection? A systematic review, pooled analysis and lessons learned. PLoS Negl Trop Dis. 2019;13(1):1–14.
23. Wilder-Smith A, Wei Y, Araújo TVB de, VanKerkhove M, Turchi Martelli CM, Turchi MD, et al. Understanding the relation between Zika virus infection during pregnancy and adverse fetal, infant and child outcomes: a protocol for a systematic review and individual participant data meta-analysis of longitudinal studies of pregnant women and their inf. BMJ Open. 2019;9(6):e026092.
24. Thorne C, Malyuta R, Semenenko I, Pilipenko T, Patel D, Bunders M, et al. The mother-to-child HIV transmission epidemic in Europe: Evolving in the East and established in the West. Aids. 2006;20(10):1419–27.
25. Mandelbrot, Mayaux, Bongain, Berrebi, Moudoub-Jeanpetit, Bénifla, et al. Obstetric factors and mother-to-child transmission of human immunodeficiency virus type 1: The French perinatal cohorts. Am J Obstet Gynecol. 1996;175(3):661–7.
26. Dunn DT, Ades AE. Estimating the HIV Vertical Transmission Rate and the Pediatric AIDS Incubation Period from Prospective Data AU. J Am Stat Assoc. 1996 Sep 1;91(435):935–43.
27. Gras L, Gilbert RE, Ades AE, Dunn DT. Effect of prenatal treatment on the risk of intracranial and ocular lesions in children with congenital toxoplasmosis. Int J Epidemiol. 2001;30(6):1309–13.
28. Gilbert RE, Gras L, Wallon M, Peyron F, Ades AE, Dunn DT. Effect of prenatal treatment on mother to child transmission of Toxoplasma gondii: Retrospective cohort study of 554 mother-child pairs in Lyon, France. Int J Epidemiol. 2001;30(6):1303–8.
29. The SYROCOT (Systematic Review on Congenital Toxoplasmosis) study group. Eff ectiveness of prenatal treatment for congenital toxoplasmosis: a meta-analysis of individual patients' data. Lancet. 2007;369(9556):115–22.
30. Stagno S, Pass RF, Dworsky ME, Henderson RE, Moore EG, Walton PD, et al. Congenital Cytomegalovirus Infection: The relative importance of primary and recurrent maternal infection. N Engl J Med. 1982 Apr;306(16):945–9.
31. Townsend CL, Forsgren M, Ahlfors K, Ivarsson SA, Tookey PA, Peckham CS. Long-term outcomes of congenital cytomegalovirus infection in Sweden and the United Kingdom. Clin Infect Dis. 2013;56(9):1232–9.

For peer review only - http://bmjopen.bmj.com/site/about/guidelines.xhtml

32. Preece PM, Blount JM, Glover J, Fletcher GM, Peckham CS, Griffiths PD. The consequences of primary cytomegalovirus infection in pregnancy. Arch Dis Child. 1983;58(12):970–5.
33. Li XL, Wei HX, Zhang H, Peng HJ, Lindsay DS. A meta analysis on risks of adverse pregnancy outcomes in Toxoplasma gondii infection. PLoS One. 2014;9(5):1–12.
34. Paz-Bailey G, Rosenberg ES, Doyle K, Munoz-Jordan J, Santiago GA, Klein L, et al. Persistence of Zika Virus in Body Fluids — Final Report. N Engl J Med. 2018;NEJMoa1613108.
35. Centers for Disease Control and Prevention. Prolonged IgM Antibody Response in People Infected with Zika Virus : Implications for Interpreting Serologic Testing Results for Pregnant. 2017;5–7.
36. Rabe IB, Staples JE, Villanueva J, Hummel KB, Johnson JA, Rose L, et al. Interim Guidance for Interpretation of Zika Virus Antibody Test Results. MMWR Morb Mortal Wkly Rep. 2016;65(21):543–6.
37. Paixão ES, Teixeira MG, Costa M da CN, Barreto ML, Rodrigues LC. Symptomatic Dengue during Pregnancy and Congenital Neurologic Malformations. Emerg Infect Dis. 2018 Sep;24(9):1748–50.
38. Cardenas I, Means RE, Aldo P, Koga K, Lang SM, Booth C, et al. Viral Infection of the Placenta Leads to Fetal Inflammation and Sensitization to Bacterial Products Predisposing to Preterm Labor. J Immunol. 2010;185(2):1248–57.
39. JL. S. Multiple imputation: a primer. Stat Methods Med Res. 1999;8(1):3–15.
40. Basurko C, Matheus S, Hildéral H, Everhard S, Restrepo M, Cuadro-Alvarez E, et al. Estimating the risk of vertical transmission of dengue: A prospective study. Am J Trop Med Hyg. 2018;98(6):1826–32.
41. Contopoulos-Ioannidis D, Newman-Lindsay S, Chow C, LaBeaud AD. Mother-to-child transmission of Chikungunya virus: A systematic review and meta-analysis. PLoS Negl Trop Dis. 2018;12(6):1–20.
42. Honein MA, Dawson AL, Petersen EE, Jones AM, Lee EH, Yazdy MM, et al. Birth defects among fetuses and infants of US women with evidence of possible zika virus infection during pregnancy. JAMA - J Am Med Assoc. 2017 Jan 3;317(1):59–68.

For peer review only

For peer review only - http://bmjopen.bmj.com/site/about/guidelines.xhtml

SUPPLEMENTARY MATERIALS

Supplementary Table S1. Schedule of tests and examinations in pregnancy, at delivery, and during paediatric follow-up, in each consortium.

Consortium	Mother	Fetus	Newborn and neonate	Pediatric follow-up
ZIKAlliance	ZIKV serology IgG & IgM, NAAT blood and urine, at booking, every 4w, at birth, and at paediatric follow-up.<br><br>NAAT placenta and amniotic fluid at birth; NAAT breast milk and saliva at birth, if evidence of infection.<br><br>TORCH at enrolment / birth, HIV at birth according to local / national guidelines.	Laboratory, pathology examination of fetal loss and stillbirth.	Clinical examination of newborn.<br><br>Newborn testing: NAAT, IgM blood, cord blood; TORCH according to local / national guidelines.	Clinical examination, anthropometry, NAAT blood and urine; IgM, IgG blood at 4w, 4m, 12m, 24m.<br><br>Transcranial U/S at 4w. Hearing, eye, neurodevelopmental assessments.
ZIKAction	IgM, IgG serology booking, 20, 28, Delivery, and at paediatric follow-up<br><br>NAAT testing, and additional U/S investigations if evidence of infection.<br><br>PRNT for sub-set of samples.	Laboratory, pathology examination of fetal loss and stillbirth.	Clinical examination of newborn<br><br>Newborn testing: IgM, IgG (serum; CSF only if clinically indicated), urine, saliva. TORCH testing if clinically indicated<br><br>NAAT, U/S and ophthalmology investigations if evidence of infection	Paediatric testing:<br><br>IgG, IgM (serum, urine, saliva) and clinical examination at: 4w, 4m, 9m, 12m, 18m and 24m. NAAT if evidence on infection<br><br>Neurodevelopmental assessments

For peer review only - http://bmjopen.bmj.com/site/about/guidelines.xhtml

ZikaPLAN	NAAT and serologic testing (PRNT, IgM, IgG3) when symptoms reported and at up to three follow-up visits.<br>TORCH, DENV, and CHIKV testing	Not routinely tested.	Clinical examination in early infancy.<br>NAAT testing in the neonatal period in some centres. TORCH testing if clinically indicated	Clinical examination of the child by specialists at 3, 6, 12, 18, 24, 36, and 48m<br>Neurodevelopmental assessments

CSF, Cerebro-spinal fluid; DENV, Dengue virus; CHIKV, Chikungunya virus; HIV, Human Immunodeficiency virus; Ig, Immunoglobulin; NAAT, Nucleic Acid Amplification Test; PRNT, Plaque Reduction Neutralisation Test; TORCH, Toxoplasmosis, Other, Rubella, Cytomegalovirus, Herpes; U/S, Ultrasound; ZIKV, Zika virus

For peer review only

For peer review only - http://bmjopen.bmj.com/site/about/guidelines.xhtml

Supplementary Table S2. Illustrative and approximate definitions of key explanatory and outcome variables. The final definitions will be determined by the Joint Diagnostics Group and Joint End-point Review Group.

Maternal Symptoms	For example: rash, fever, headache, joint pain
Maternal Infection in Pregnancy (MIP) status, established only on the basis of maternal testing	Confirmed: NAAT, Seroconversion<br>Suspected: Serological tests of recent infection, including: IgM, IgG3, avidity<br>No Evidence of MIP: Maternal testing protocol was followed, but none of the above were positive.<br>No MIP: As above but with IgG negative at or near time of delivery
Laboratory markers of Congenital Infection (CI)	Present: NAAT or IgM any time in first 7 days;<br>Absent: all other findings
Most likely trimester of onset of maternal infection	The highest available from the following hierarchy:<br>• Date of first NAAT positive test minus average duration of viremia<br>• Seroconversion: Midpoint between last serological negative and last positive<br>• Serological tests of recent infection: Date of first positive
Signs and symptoms compatible with Congenital Zika Syndrome (CZS)	A definition will be prepared by the Joint End-point Review Group, based on best evidence available. *The intention will be to produce a definition that is virtually 100% specific for congenital ZIKV infection in mothers exposed to a ZIKV outbreak, especially if other TORCH infections can be ruled out.*
Other Potentially Zika-related Outcomes (OPZRO)	A definition will be prepared by the Joint End-point Review Group, based on best evidence available.

Ig, Immunoglobulin; NAAT, Nucleic Acid Amplification Test.

For peer review only - http://bmjopen.bmj.com/site/about/guidelines.xhtml

Supplementary Table S3. Prospectively ascertained Maternal Infection in Pregnancy (MIP) status and evidence on which it is based. NB: Data from the same woman can appear in more than one of the last four columns

Centre	Total women	Prospectively ascertained MIP status				Evidence for Confirmed or Suspected MIP			
		Confirmed MIP	Suspected MIP	No Evidence of MIP	No MIP	NAAT	Sero-conversion	IgM/ IgG3	PRNT
1									
2									
…									
etc									

Ig, Immunoglobulin; NAAT, Nucleic Acid Amplification Test; PRNT, Plaque Reduction Neutralisation Test.

Supplementary Table S4. Summary of evidence on laboratory and clinical markers of congenital infection (CI) and evidence on which it is based. NB: Data from the same fetus/newborn can appear in more than one column

		Markers of congenital infection						Other evidence of congenital infection			
Centre	Total number fetus/newborn	Laboratory or clinical markers of congenital infection	Laboratory markers of congenital infection only	Signs and symptoms compatible with CZS	NAAT	IgM	No markers of congenital infection	Microcephaly	Neurological abnormalities	Ocular abnormalities	Other, to be defined
1											
2											
…											
etc											

CZS, Symptoms compatible with Congenital Zika Syndrome; IgM/G, Immunoglobulin M/G; NAAT, Nucleic Acid Amplification Test; OPZRO, Other potentially Zika-related symptoms; PRNT, Plaque Reduction Neutralisation Test.

For peer review only - http://bmjopen.bmj.com/site/about/guidelines.xhtml

Supplementary Table S5. Distributions of signs and symptoms by laboratory markers of congenital infection and prospectively ascertained maternal infection in pregnancy (MIP) status**.** The breakdown shown is an example. Other examples might be "Termination of pregnancy, Pregnancy loss, Stillbirth, Livebirth"; or "Sensorineural hearing loss (SNHL), No SNHL "

**CENTRE**			
**Sign or symptom in fetus, newborn, or infant**	**MIP with laboratory markers of congenital infection**	**MIP with no laboratory markers of congenital infection**	**No MIP**
Signs and symptoms compatible with Congenital Zika Syndrome (CZS)			
Other potentially Zika-related outcomes (OPZRO)			
Asymptomatic			
**TOTAL = 100%**			

For peer review only
